# Synthesis and Pharmacological In Vitro Investigations of Novel Shikonin Derivatives with a Special Focus on Cyclopropane Bearing Derivatives

**DOI:** 10.3390/ijms22052774

**Published:** 2021-03-09

**Authors:** Nadine Kretschmer, Antje Hufner, Christin Durchschein, Katrin Popodi, Beate Rinner, Birgit Lohberger, Rudolf Bauer

**Affiliations:** 1 Department of Pharmacognosy, Institute of Pharmaceutical Sciences, University of Graz, Beethovenstr. 8, 8010 Graz, Austria; nadine.kretschmer@uni-graz.at (N.K.); christin.durchschein@hotmail.com (C.D.); katrin.popodi@gmx.at (K.P.); 2 Department of Pharmaceutical Chemistry, Institute of Pharmaceutical Sciences, University of Graz, Universitaetsplatz 1, 8010 Graz, Austria; antje.huefner@uni-graz.at; 3 Division of Biomedical Research, Medical University of Graz, Roseggerweg 48, 8036 Graz, Austria; beate.rinner@medunigraz.at; 4 Department of Orthopedics and Trauma, Medical University Graz, Auenbruggerplatz 5, 8036 Graz, Austria; birgit.lohberger@medunigraz.at

**Keywords:** shikonin derivatives, synthesis, apoptosis, cell cycle, melanoma

## Abstract

Melanoma is the deadliest form of skin cancer and accounts for about three quarters of all skin cancer deaths. Especially at an advanced stage, its treatment is challenging, and survival rates are very low. In previous studies, we showed that the constituents of the roots of *Onosma paniculata* as well as a synthetic derivative of the most active constituent showed promising results in metastatic melanoma cell lines. In the current study, we address the question whether we can generate further derivatives with optimized activity by synthesis. Therefore, we prepared 31, mainly novel shikonin derivatives and screened them in different melanoma cell lines (WM9, WM164, and MUG-Mel2 cells) using the XTT viability assay. We identified (*R*)-1-(1,4-dihydro-5,8-dihydroxy-1,4-dioxonaphthalen-2-yl)-4-methylpent-3-enyl 2-cyclopropyl-2-oxoacetate as a novel derivative with even higher activity. Furthermore, pharmacological investigations including the ApoToxGlo^TM^ Triplex assay, LDH assay, and cell cycle measurements revealed that this compound induced apoptosis and reduced cells in the G1 phase accompanied by an increase of cells in the G2/M phase. Moreover, it showed hardly any effects on the cell membrane integrity. However, it also exhibited cytotoxicity against non-tumorigenic cells. Nevertheless, in summary, we could show that shikonin derivatives might be promising drug leads in the treatment of melanoma.

## 1. Introduction

Malignant melanoma belongs to the most dangerous type of skin cancer and arises from melanocytes [1]. Only 2–3% of all diagnosed skin cancers are melanomas, however, they account for approx. 75% of all skin cancer deaths [2]. Melanomas are divided into four different stages: stages I and II display local primary tumors, stage III represents tumors with locoregional metastases, and stage IV tumors with distant metastases. In addition, tumors are typically categorized by thickness and ulceration [1,3]. When melanomas are detected at an early clinical stage and treated appropriately, the 10-year survival rate is around 98% [4]. However, when the tumor metastasizes, the survival rate drops dramatically and therapeutic success is limited due to tumor recurrence, severe side effects, and/or the development of resistances [2]. According to the Melanoma Research Alliance, the five-year survival rate for metastatic melanoma is currently only 22.5%. Therefore, the search for novel drug leads remains an important task.

In the discovery of novel therapeutics, natural products have always played a central role. In the case of anticancer drugs, the FDA approved in total 247 new chemical entities between 1981 and 2019, including 185 (75%) small molecules. Only 15.7% of the small molecules were totally synthetic compounds. All others were natural products or inspired by them [5]. Some of them, such as vinblastine, campthothecin, or taxol are most important in cancer chemotherapy today [6]. Shikonin (**1**) and its derivatives are naturally occurring, biologically active naphthoquinones and can be found in several members of the Boraginaceae family such as *Arnebia euchroma* (Royle ex Benth.) I.M. Johnst., *Lithospermum erythrorhizon* Siebold et Zucc., and *Onosma paniculata* Bureau & Franchet Their pharmacological spectrum comprises anti-oxidative, anti-inflammatory, anti-virus, and anti-cancer activities [7,8,9,10]. Plants containing these constituents are traditionally used for the treatment of eruptive exanthema, eczema, skin infections, burns, constipation, scalds, and cancer [11]. Concerning the anti-cancer activity, it has been reported that shikonin and derivatives led to induction of apoptosis, cell cycle arrest, and autophagy and inhibited cell growth and metastasis [7,8,9,10]. Shikonin was also shown to kill cancer cells synergistically in combination with established cancer therapeutics such as erlotinib [12].

In previous studies, we could show that β,β-dimethylacrylshikonin (**2**) was the most cytotoxic compound isolated from the roots of *O. paniculata* and exhibited the strongest cytotoxicity toward several melanoma cell lines [13,14]. Ongoing studies revealed that **2** favored catabolic processes and caused generation of reactive oxygen species, loss of mitochondrial membrane potential, and the upregulation of NOXA expression [15,16]. Finally, this led to apoptosis, autophagy, and cell cycle arrest. Using a mouse xenograft model, we could show that **2** exhibited also promising in vivo effects [16]. However, we were wondering whether the structure of **2** can be modified to further improve and optimize the pharmacological effects. In a first attempt, we ended up with cyclopropylacetylshikonin (**3**), which exhibited lower IC_50_ values than **2** in metastatic melanoma cell lines and induced apoptosis as well [17]. In the current study, we followed a two-fold strategy: (a) optimizing the cyclopropylacetyl group of our last hit **3** and (b) mounting a broad spectrum of structural features within the acyl residue of shikonin. To cover a broader spectrum of compounds and for comparison reasons, we also included some already known synthetic shikonin derivatives. However, most of the compounds are novel or not yet investigated in this context.

## 2. Results and Discussion

### 2.1. Syntheses of Shikonin Derivatives

**Figure 1:**
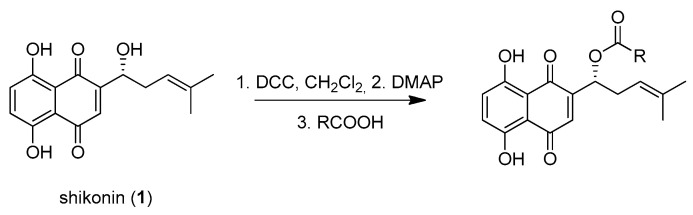
Acylation of shikonin (**1**).

Shikonin derivatives have been shown to exhibit potent anti-cancer activities [7,8,9,10]. It was reported that the naphthoquinone scaffold with its hydroxyl groups is necessary for the pharmacological activity [18,19,20,21] and that the side chain modifies the activity. Similar features in the well-known anthracycline antitumor antibiotics and mitoxantrone seem to be important for DNA binding and bioavailability [22]. Therefore, we decided to focus on modifying the side chain. Shikonin (**1**, 100% R-isomer [17]) was chosen as starting material. Acylation of **1** was accomplished via Steglich esterification in dichloromethane with the corresponding carboxylic acid, as well as dicyclohexylcarbodiimide (DCC) as a coupling reagent and 4-dimethylaminopyridine (DMAP) as a catalyst (Figure 1) [17,20,23,24,25,26,27]. NMR spectra of all synthesized derivatives can be found in supplementary material (chapter 2).

The first strategy was the optimization of the cyclopropylacetate in **3** [17]. In the bicyclus **4**, 1′ and 2′ positions of cyclopropylacetate are connected as part of a cyclohexane. Replacement of the methylene spacer of **3** with a CO group, a C,C double bond, and an ethylene group resulted in **5**, **6**, and **7**, respectively.

**Figure 2:**
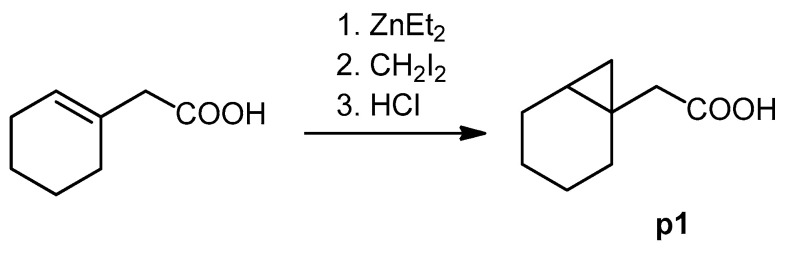
Synthesis of the bicyclic acetic acid **p1**.

**Figure 3:**
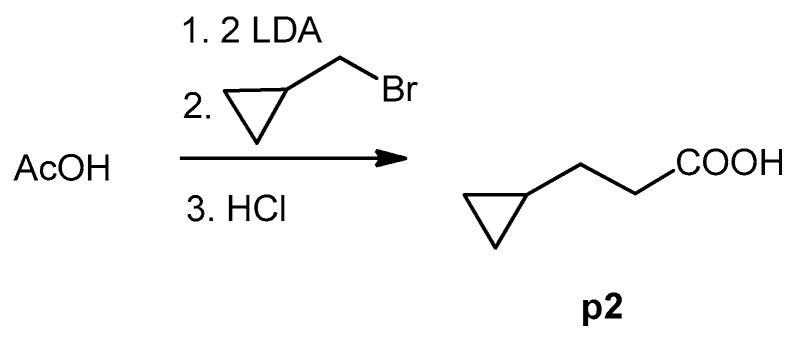
Synthesis of 3-cyclopropylpropanoic acid (**p2**).

**Figure 4:**
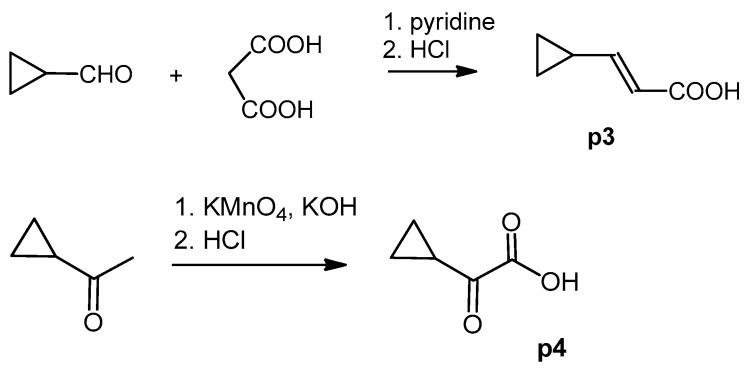
Synthesis of precursor acids **p3** and **p4**.

The cyclopropane precursor acids were synthesized in our laboratory. As outlined in Figure 2, the bicyclic acetic acid **p1** was prepared from the corresponding (1-cyclohexenyl)-2-acetic via Furukawa modification of the Simmons–Smith reaction analogously to a procedure of Renaud and Fox [28]. 3-Cyclopropylpropanoic acid (**p2**) was prepared by α-alkylation of acetic acid in analogy to a procedure described by Barczak and Jarvo [29] (Figure 3). The Knoevenagel condensation of cyclopropanecarbaldehyde with malonic acid [30] resulted in β-cyclopropylacrylic acid **p3**. 2-Cyclopropyl-2-oxoacetic acid (**p4**) was obtained by oxidation of acetylcyclopropane with KMnO_4_ in a combination of the procedures given by Prokopenko et al. [31] and Xu et al. [32] (Figure 4). Details of the syntheses of the precursor acids are described in material and methods. Their NMR spectra can be found in the supplementary material (chapter 3). In all cases (precursor acids and shikonin derivatives), the structure and purity of each compound were analyzed using 1D and 2D NMR, LC-MS, and IR experiments.

The second goal was to produce a broad spectrum of structural features within the acyl residue of shikonin to find potential novel drug leads. For better comparison and a more complete picture, we also included some already known derivatives. Phenylacetate **8** and cinnamate **9** showed, for example, cytotoxicity in previous studies and were synthesized for comparison reasons [26,33]. As there have been no reports about any shikonin alkynylacyl esters yet, we synthesized tetrolate **10**, 2-butynoate **11**, and 3-phenylpropiolate **12** to investigate the influence of a triple bond. 

Except for three recently published studies about benzoylacrylates [34], succinamides, and maleinamides [35,36], all carbonyl groups in the acyl chain of shikonin or alkannin esters were part of acetoxy groups mainly derived from β-hydroxy acylates. However, the substances of Sun et al. [34] are of unknown chirality, among them the *p*-methylbenzoylacrylate **13**. We also had a look on derivatives with the carbonyl carbon as a part of the carbon chain—either as a ketone or as part of a diester. Pyruvate **14** and 2-oxo-2-phenylacetate **15** represent α-ketoacylates. γ-Carbonyls are found in the keto esters **13** and **16** as well as in the diesters derived from monomethyl succinate **17** and monoethyl fumarate **18**.

The known short chain alkyl and alkenyl esters **19** (isobutyrate) [37], **20** (isovalerate) [38], **21** (crotonate) [39], and **22** (sorbate) [38] were used to examine the influence of the saturation and branching on the activity. Compounds **19, 20**, and **22** are already known to inhibit the growth of other types of cancer cells [23,37]. However, there have been no reports about their effects in melanoma cells. Derivative **21** was one of the very first non-natural 1′-*O*-acyl shikonines and shialkines [39], but no data about the biological activity have been reported so far.

Other features, which we investigated, were carbocyclic and heterocyclic aryl groups. The most basic structures, i.e., phenylacetyl, benzoyl, and diphenylacetyl (**8**, **23**, **24**), were among the early synthetic 1′-O-acyl shikonines and shialkines. They were reported to bind to tubuline [38] and were cytotoxic against several cancer cell lines [23,28,40], however, no data were published about their effects in melanoma cells. Shikonin cinnamate **9** was intensively explored for its anticancer properties, too [26,41,42]. We used these derivatives to investigate the influence of the α,β double bond with the help of 3-phenylpropionate **25** [36].

Additionally, as a *p*-dimethylamino group improved the activity of phenylacetate **8 [40]**, we prepared the *p*-dimethylaminocinnamate **26**. Nicotinate **27** was reported as quite inactive [27,43], but more active than benzoate **23** [44]. Therefore, we tested nicotinate **27** and, additionally, isonicotinate **28**. According to literature data, 3-(3-indolyl)propionate **29** showed up as the most active compound in another study [21]. As shikonin 2-furylcarboxylate was reported to be active [45] and we found that cinnamate was more active than benzoate, we prepared 3-(2-furyl)acrylate **30**. The cytotoxicity of bromoacetate **31** was reported among other halogenated acetates [46]. Therefore, we prepared **31** together with novel ω-bromoalkylacylates **32**, **33**, and **34** to study the influence of chain length.

### 2.2. Results of the XTT Screening

**Figure 5:**
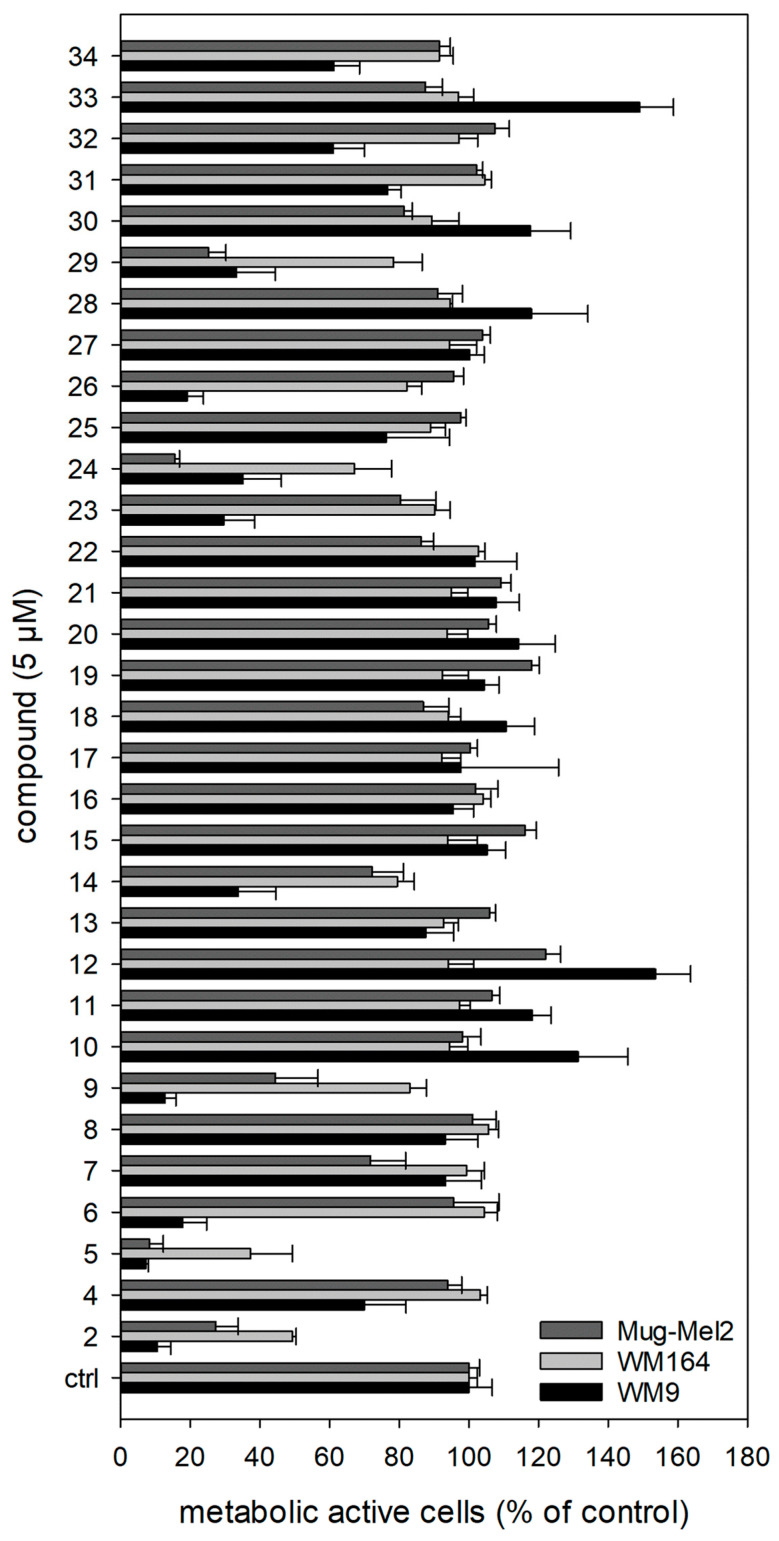
Results of the XTT assay. For clarity reasons, only the results of the treatment with 5.0 µM for 72 h are shown. The complete results can be found in the Supplementary Material (Figures S1 and S2). **2** was tested as a reference at 5.0 µM. The strongest cytotoxicity was found for **5** (*n* = 6, mean ± sem). Vinblastine was used as positive control. At a concentration of 0.01 nM, it reduced the cell viability compared to control cells to: WM9 cells: 23.8 ± 1.5%, WM164 cells: 59.4 ± 4.4 %, and MUG-Mel2 cells: 65.0 ± 6.9 % (*n* = 6, mean ± sem, 72 h of treatment).

All prepared shikonin derivatives (see Table 1, compounds **4** to **34**) were subjected to a cytotoxicity screening using the XTT viability assay. This assay is based on the activity of mitochondrial dehydrogenases. These enzymes cleave the yellow tetrazolium salt XTT leading to an orange formazan. This conversion only occurs in viable cells and can be directly quantified by measuring the absorbance [47]. Melanoma cells were treated with 1.0 µM, 5.0 µM, and 10.0 µM of each derivative for 72 h (Figure 5; Figures S1 and S2 in the Supplementary Material). In brief, the most active derivatives were **5**, **6**, **9**, **14**, **24**, and **29**, with **5** being the most cytotoxic. Its IC_50_ values are listed in Table 2. Moreover, the derivatives **11** and **28** showed no cytotoxicity at 10 µM, the derivatives **4**, **16**, **20**, and **27** exhibited only a very weak cytotoxicity at 10 µM (Supplementary Figures S1 and S2).

Discussed in more detail, the results showed that the sensitivity of the three cell lines used was different. In general, WM164 cells reacted least to the treatment. Only a few compounds showed moderate activity, for example, the known derivatives **9**, **24**, and **29**, as well as the novel derivative **14**. WM9 cells were most affected by the derivatives. Next to **5** (novel), compounds **6** (novel), **9** (known), and **14** (novel) were the most cytotoxic derivatives in this cell line. MUG-Mel2 cells reacted in the case of some compounds (**18**, **19**, **21**, **22**, **30**, **33**, and **34**) more sensitive to the treatment than the WM9 and WM164 cells. This is of special interest, because MUG-Mel2 cells are NRAS mutated, while WM9 cells and WM164 cells are BRAF mutated. The mutational status is another category in melanoma diagnosis because some mutations lead to a poorer prognosis than others do. Around 50% of all melanomas exhibit a mutation in the BRAF gene and another 25% a mutation in the NRAS gene [48]. Tumors carrying a BRAF mutation are currently typically treated with a combination of MEK and BRAF inhibitors, however, tumor resistances often develop [49]. In the case of NRAS mutated melanoma, the therapeutic success is even lower because they are more difficult to treat [50]. This means that the most active derivatives of our study, can also display lead compounds for the development of further novel shikonin derivatives with a special focus on NRAS mutated melanoma cells.

Returning to the present results, we firstly modified the structure of our previous hit **3**. Further substituents at the cyclopropane reduced the activity against all cell lines significantly. The modification of the spacer showed unequal effects: replacement of the methylene group with carbonyl (**5**) or ethylene (**7**) showed a similar activity on the cell lines with **5** being more cytotoxic towards WM9 cells. An α,β unsaturation resulted in less activity towards MUG-Mel2 cells but in an increased effect in WM9 cells.

When analyzing the other investigated structural features, it became obvious that hydrogenation of the acyl side chain of **2** reduced the cytotoxicity to a very low level in all cell lines. Shortening the side chain and removal of the branching β-methyl are among the few modifications, which had a bigger influence on the activity in WM9 cells than in MUG-Mel2 cells. The additional conjugated double bond in the sorbate **22** had no significant effect. However, the attachment of a phenyl group restored the properties to the activity level of **2**. The *p*-dimethylamino group had no effect on the activity and, again, hydrogenation of the exocyclic double bond of the cinnamic residue lowered activity significantly. The removal or shortening of the spacer slightly improved the activity (compare **8** and **23**). The replacement of the phenyl ring of **9** with a furan moiety (**30**) abolished the activity in WM9 cells but kept some effects in MUG-Mel2 cells. The diphenylacetate **24** and indolylpropionate **29** appeared as active as the cinnamate **9** and as **2**. The replacement of the phenyl ring in benzoate **23** by a 3-pyridine or 4-pyridine abolished the cytotoxicity.

Moreover, the activity of the γ oxo esters **13**, **16**, **17**, and **18** was generally low. While WM9 cells were less affected by saturated and unsaturated compounds, MUG-Mel2 cells showed a slight sensitivity towards unsaturated compounds. The introduction of an α carbonyl in 1′-*O*-acetylshikonin resulted in pyruvate **14** which showed an improved activity in WM9 cells, but a reduced one in MUG-Mel2 cells. Also, α-carbonylation of the benzoate **23**, resulting in 2-oxo-2-phenylacetate **15**, affected the cell lines differently. All the alkynylacyl esters (**10**, **11**, and **12**) showed no or only marginal effects on all cell lines. Even though ω-bromo compounds **31**, **32, 33**, and **34** appear more active, their potency is inferior to **2** and display no regularity concerning chain length and activity.

In summary, **5** appeared as the most cytotoxic compound in this series and, therefore, was investigated pharmacologically in more detail. First, **5** was also tested in non-tumorigenic HEK293 cells. As shown in Table 2, the IC_50_ value after 72 h was 3.4 µM, which is 2.3-fold higher than in WM9 cells, but also 1.3-fold lower than in WM164 cells. The cytotoxicity of chemotherapeutics against non-tumorigenic cells is a known problem in cancer therapy and one reason of undesirable side effects. For example, vinblastine and doxorubicin are two well-known and often clinically administered chemotherapeutics. In in vitro experiments, both also show cytotoxicity towards non-tumor cells to a similar or even greater extent [14,51,52]. However, it is difficult to extrapolate from in vitro to in vivo effects. Nevertheless, there is a certain risk that **5** will also cause side effects as they are already known for clinically used chemotherapeutics. However, it has already been shown for shikonin that there are ways to overcome this problem. Fayez et al. [53] reported recently that a combination of shikonin and silver nanoparticles synergistically inhibited the growth of lung cancer cells. Wang et al. [54] have shown that shikonin and JQ1, a bromodomain and extra-terminal motif (BET) inhibitor, encapsulated in lactoferrin nanoparticles changed the tumor immune microenvironment, activated immunogenic cell death, repolarized protumor phenotype, tumor-associated macrophages, and repressed glucose metabolism. They concluded that their system can be developed as a novel cancer immunotherapy due to several synergistic advantages. Another strategy could be a further development of our compounds to oxime derivatives. Huang et al. [55] demonstrated that such derivatives exhibited strong cytotoxicity towards cancer cells, but only a low cytotoxicity towards human skin fibroblasts.

### 2.3. Pharmacological Effects of Cyclopropyloxoacetate 5

**Figure 6:**
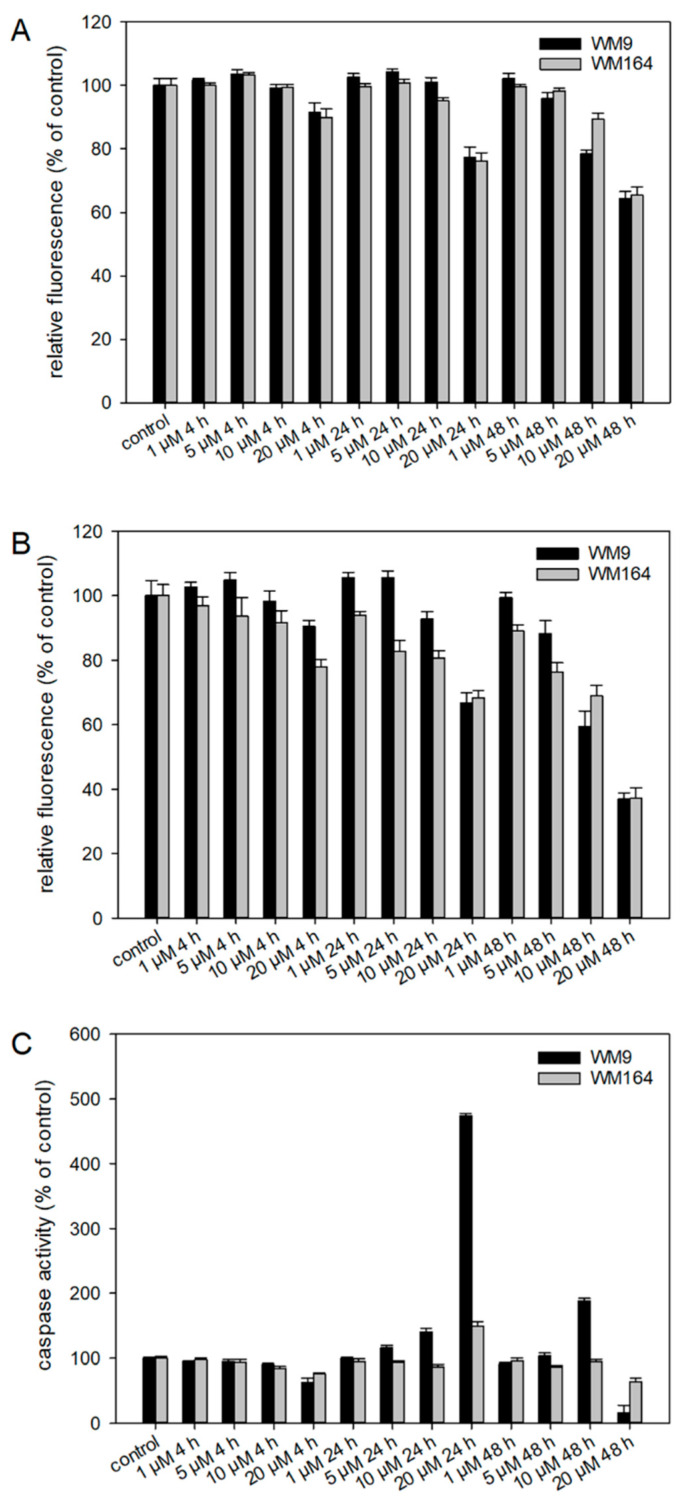
Results of the ApoToxGlo™ Triplex Assay. WM9 and WM164 cells were treated with 1 µM, 5 µM, 10 µM, and 20 µM of **5** for 4 h, 24 h, or 48 h (*n* = 6, mean ± sem). (**A**) Viability of the cells measured as relative fluorescence of control cells. (**B**) Cytotoxicity of **5** towards the cells measured as relative fluorescence of control cells. (**C**) Activity of caspases 3 and 7 indicative for apoptosis induction. Staurosporine (25 µM) served as positive control (apoptosis increase in WM9 cells after 24 h: 1193.9 % and after 48 h: 297.4%; in WM164 cells after 24 h: 989.1% and after 48 h: 362.6%).

To investigate the effects of **5** in more detail, several pharmacological assays were performed. Since **5** was most active in the metastatic cell line WM9 (BRAF mutated), we decided to use this cell line and, in addition, another BRAF mutated cell line (WM164) for comparison. Using the ApoToxGlo™ Triplex Assay, we investigated the effects regarding cell viability, cytotoxicity, and apoptosis induction in more detail (Figure 6). The advantage of this assay is the combination of three assays in one single assay well. It has already been shown by our [14,16] and other groups [8,9] that several shikonin derivatives induced apoptosis in a variety of cancer types. We treated the cells with up to 20 µM of **5** for up to 48 h. Regarding cell viability, we found no statistically significant changes up 10 µM after 4 h and 24 h and up to 5 µM after 48 h. In the case of cytotoxicity, the fluorescence intensity decreased time- and dose-dependently, which can be an indicator for primary necrosis in combination with a reduced viability. Concerning apoptosis, we found a clear increase in caspase 3/7 activity after 24 h and 48 h in WM9 cells and a slight increase after 24 h in WM164. This agrees with the effects of our previous hit **3** [17] even if the activation of caspase 3/7 was weaker in case of **5**. In summary, our results indicate that caspases are activated during the treatment with **5** further indicating that the cells undergo apoptotic cell death.

**Figure 7:**
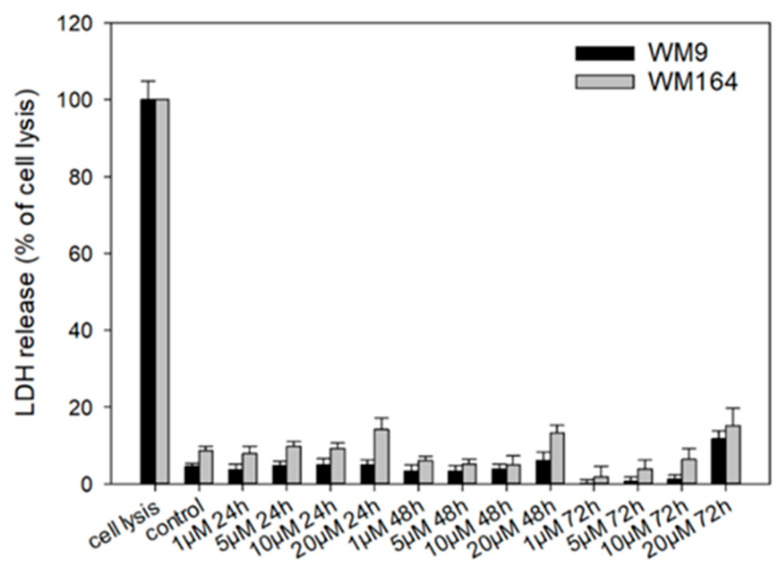
Results of the LDH assay. WM9 and WM164 cells were treated with 1 µM, 5 µM, 10 µM, and 20 µM of **5** for 24 h, 48 h, or 72 h (*n* = 9, mean ± sem). Results are displayed as percentage of cell lysis. Control = vehicle treated cells (0.5% EtOH). Only at 20 µM, slight increases in LDH release were found.

To investigate necrosis induction, we performed the CytoTox 96^®^ Non-Radioactive Cytotoxicity Assay (LDH assay) (Figure 7). Lactate dehydrogenase (LDH) is released into the cell culture medium when the cell membrane is damaged. Therefore, this enzyme can be used as a marker for measuring necrotic cell death [56]. Cells were treated with **5** with up to 20 µM for up to 72 h. We found no LDH release up to 10 µM, which is 6.7-fold higher than the IC_50_ in WM9 cells and 2.2-fold higher than the IC_50_ in WM164 cells after 72 h. When the cells were treated with 20 µM **5**, a slight increase of LDH release was found. However, compared to the maximal possible LDH release during complete cell lysis, the measured amount of LDH released by treated cells was generally quite low. In addition, these changes at 20 µM were not statistically significant in WM164 cells: 24 h: *p* = 0.182; 48 h: *p* = 0.115; 72 h: *p* = 0.269. In WM9 cells, the changes were statistically significant only at the highest tested concentration of 20 µM (*p* = 0.0197). In summary, necrosis seems to play only a minor role during the observed cells death at the concentrations tested. Nevertheless, it has been reported for shikonin that it induced necrosis in cancer cells such as lung and gastric cancer cells [57,58]. Cellular lysis appears also during the process of necroptosis [59] which has also been reported for shikonin [60]. Therefore, the increase of LDH release could also point to the induction of necroptosis by **5**, which should be investigated in future studies.

**Figure 8:**
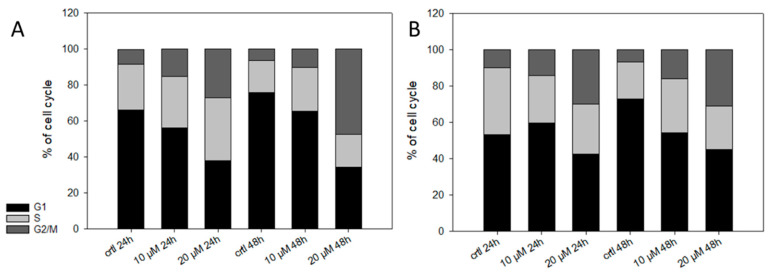
Effects of **5** on the cell cycle. (**A**) WM9 and (**B**) WM164 cells were treated with 10 µM and 20 µM of **5** for 24 h or 48 h (*n* = 6, mean). Results are displayed as percentage of cell cycle distribution. Control = vehicle treated cells (0.5% EtOH). **5** influence the distribution of the cell cycle only at high concentrations.

Finally, we investigated the effect of **5** on the cell cycle. It has been reported that shikonin derivatives are able to bind to tubulin and, therefore, lead to cell cycle arrest [43,61]. Also, in the case of **2**, we found a cell cycle arrest in different types of melanoma cell lines [14]. Therefore, we treated the cells with up to 20 µM of **5** for up to 48 h (Figure 8). Only at higher concentrations, **5** changed the cell cycle distribution statistically significant (Table 3). We also tested the effect of 5 µM **5** on the cell cycle. However, **5** had no effect on the cell cycle at this concentration (data not shown). Our results agree with other studies since Baloch et al. [43] reported a quite high IC_50_ of 25.28 µM for shikonin concerning its inhibitory effect on tubulin polymerization.

## 3. Materials and Methods

### 3.1. Chemicals

Shikonin was purchased from Chengdu Biopurify Phytochemicals Ltd. (Chengdu, China). β,β-Dimethylacrylshikonin (**2**) was isolated from dried roots of *Onosma paniculata* Bureau & Franchet (Boraginaceae) and identified as reported previously [14].

### 3.2. Synthesis of Shikonin Derivatives

The synthesis of shikonin derivatives is described below. Their NMR spectra can be found in the supplementary material, chapter 2. The purity of all compounds was analyzed using NMR experiments and always exceeded 95%. LC-ESI-MS measurements were performed on a Dionex Ultimate 3000 UHPLC (Thermo, San José, CA, USA). It was coupled with a Thermo LTQ XL linear ion trap mass spectrometer equipped with an H-ESI II probe (negative mode). The acquisition wavelength was 500 nm, source heater temperature: 250 °C, capillary temperature: 200 °C, source voltage: 3.5 kV, sheath gas flow: 50 arbitrary units, capillary voltage: −14 V, and auxiliary gas flow: 10 arbitrary units. A Kinetex C18 column (2.6 µm, 100 × 2.10 mm, Phenomenex, Torrance, CA, USA) was used as stationary phase. Water (A) and acetonitrile (B) were used as mobile phases (gradient program: 0–45 min: 55–100% B, flow rate: 0.2 mL/min, column temperature: 30 °C).

### 3.3. General Procedure for the Acylation of Shikonin

A solution of shikonin in abs. CH_2_Cl_2_ (0.1 mmol/5 mL) was cooled to 0 °C under argon atmosphere and DCC was added. After 15 min of stirring, DMAP was added. After an additional 15 min stirring, the corresponding acid was added and stirred for another 5.5 h to 5 days with slowly warming up to room temperature. Afterwards, 1 mL cyclohexane/0.1 mmol shikonin was added and the mixture was concentrated at room temperature and under reduced pressure to ca. 0.5 mL/0.1 mmol shikonin. The mixture was then filtered over 3 mm silica and 2 mm celite^®^ (eluent: petroleum ether/CH_2_Cl_2_ = 1:0 to 1:2). The resulting fractions were evaporated and subjected to flash CC and/or repeated PTLC (cyclohexane/CH_2_Cl_2_ mixtures).

*(R)-1-(1,4-Dihydro-5,8-dihydroxy-1,4-dioxonaphthalen-2-yl)-4-methylpent-3-enyl 2-(bicyclo[4 .1.0]heptan-1-yl)acetate (**4**),* 50 µmol Shikonin, 0.18 mmol DCC, 15 µmol DMAP and 57 µmol 2-(1-bicyclo[4.1.0]heptyl)acetic acid (**p1**); reaction time 15 h; CC on silica (4 g; cyclohexane to cyclohexane/CH_2_Cl_2_ = 2:1; PTLC on silica with cyclohexane/CH_2_Cl_2_ = 2:1 (three times developed); **4**, yield: 25%. **4**: R_f_ = 0.49 (silica, CH_2_Cl_2_); IR (ATR): 2990 (w), ≈2950 (br) (OH), 2927 (w), 2856 (w), 1736 (m) (C=O), 1607 (s), 1568 (m), 1451 (m), 1263(m), 1230 (m), 1202 (m) (COC), 1141 (s), 782 (m) cm^−1^; ^1^ H-NMR (CDCl_3_): 0.33, 0.35 (2t, 2H *J* ≈ 5 Hz H-7′’), 0.50–0.58 (m, 2H, H-7′’), 0.84–0.92 (m, 2H, H-2′’), 1.09–1.37 (m, 8H, H-4′’, H-5′’), 1.55–1.64 (m, 2H, H-3′’), 1.58 (s, 6H, H-6′), 1.69 (s, 6H, H-5′), 1.68–1.80 (m, 4H, H-6′’), 1.90–2.00 (m, 2H, H-3′’), 2.20–2.34 (m, 4H, H-α), 2.42–2.52 (m, 2H, H-2′), 2.58–2.67 (m, 2H, H-2′), 5.14 (tquint, *J* = 7.3, 0.9 Hz, 2H, H-3′), 6.04 (*J* = 7.3, 4.4, 0.8 Hz, 2H, H-1′), 7.00 (d, *J* = 0.9 Hz, H-3), 7.01 (d, *J* = 1.0 Hz, 1H, H-3), 7.19 (s, 4H, H-6, H-7), 12.43 (s, 2H, C5-O*H*), 12.59 (s, 2H, C8-O*H*); ^13^ C-NMR (CDCl_3_): *δ* 16.7 (C-1′’), 2x 17.0 (C-7′’), 17.7, 17.8 (C-2′’), 18.0 (C-6′), 2x 21.0 (C-4′’), 2 × 21.4 (C-5′’), 2 × 23.7 (C-3′’), 25.8 (C-5′), 28.6, 28.7 (C-6′’), 32.9, 33.0 (C-2′), 2x 46.3 (C-α), 2 × 69.2 (C-1′), 111.6 (C-4a), 111.8 (C-8a), 118.0 (C-3′), 2 × 131.5 (C-3), 132.8 (C-7), 2 × 132.8 (C-6), 135.9 (C-4′), 148.5 (C-2), 2 × 166.9 (C-5), 2 × 167.4 (C-8), 171.5 (COO), 2 × 176.8 (C-1), 2 × 177.3, (C-4); MS (ESI^−^) m/z (%): 423.24 (100) [M-H]^−^, [M-H]^−^ calculated for C_25_H_28_O_6_: 423.1808.

*(R)-1-(1,4-Dihydro-5,8-dihydroxy-1,4-dioxonaphthalen-2-yl)-4-methylpent-3-enyl 2-cyclopropyl-2-oxoacetate (**5**)*, 0.1 mmol Shikonin, 0.22 mmol DCC, 50 µmol DMAP and 0.15 mmol 2-cyclopropyl-2-oxoacetic acid (**p4**) reaction time 15 h; CC on silica (4 g; hexanes/CH_2_Cl_2_ = 1:0 to hexanes/CH_2_Cl_2_ = 0:1); PTLC on silica (developed four times with cyclohexane/CH_2_Cl_2_ = 2:1 and twice with cyclohexane/CH_2_Cl_2_ = 1:1); **5**, yield: 45%. **5**: R_f_ = 0.34 (silica, CH_2_Cl_2_); IR (ATR): 2971 (w), ≈2950 (br) (OH), 2915 (w), 2857 (w), 1735 (m) (C=O), 1715 (m) (C=O), 1608 (s), 1568 (m), 1451 (m), 1261 (s), 1230 (s), 1203 (s) (COC), 1057 (s), 778 (m) cm^−1^; ^1^H-NMR (CDCl_3_): 1.16–1.22 (m, 2H, H-2′’, H-3′’), 1.25–1.31 (m, 2H, H-2′’, H-3′’), 1.59 (s, 3H, H-6′), 1.68 (s, 3H, H-5′), 2.61 (dtm, *J* = 15.0, 7.5 Hz, 1H, H-2′), 2.67–2.76 (m, 2H, H-2′, H-1′’), 5.14 (tm, *J* = 7.3 Hz, 1H, H-3′), 6.17 (ddd, *J* = 7.1, 4.8, 0.5 Hz, 1H, H-1′), 7.11 (d, *J* = 0.7 Hz, 1H, H-3), 7.18 (s, 2H, H-6, H-7), 12.40 (s, 1H, C5-O*H*), 12.59 (s, 1H, C8-O*H*); ^13^C-NMR (CDCl_3_): *δ* 2 × 14.3 (C-2′’, C-3′’), 18.0 (C-6′), 18.2 (C-1′’), 25.8 (C-5′), 32.8 (C-2′), 71.4 (C-1′), 111.6 (C-4a), 111.8 (C-8a), 117.0 (C-3′), 131.4 (C-3), 133.3 (C-7), 133.6 (C-6), 136.9 (C-4′), 146.2 (C-2), 159.9 (COO), 168.8 (C-5), 169.3 (C-8), 174.6 (C-1), 176.2 (C-4), 193.3 (α-CO); MS (ESI^−^) m/z (%): 383.06 (100) [M-H]^−^, [M-H]^−^ calculated for C_21_H_20_O_7_: 383.1131.

*(R,E)-1-(1,4-Dihydro-5,8-dihydroxy-1,4-dioxonaphthalen-2-yl)-4-methylpent-3-enyl 3-cyclopropyl-2-propenoate (**6**),* 0.1 mmol Shikonin, 0.20 mmol DCC, 30 µmol DMAP and 0.10 mmol β-cyclopropylacrylic acid (**p3**); reaction time 17 h; two consecutive PTLC on silica (developed twice with cyclohexane/CH_2_Cl_2_ = 2:1 each). **6**, yield: 8 %. **6**: R_f_ = 0.29 (silica, CH_2_Cl_2_). IR (ATR): 2917 (m), ≈2950 (br) (OH), 2851 (w), 1717 (s) (C=O), 1643 (m), 1609 (vs), 1570 (m), 1453 (m), 1263 (s), 1203 (s) (COC), 1140 (s), 779 (w) cm^−1^; ^1^H-NMR (CDCl_3_): 0.63–0.73 (m, 2H, H-2′’, H-3′’), 0.95–1.04 (m, 2H, H-2′’, H-3′’), 1.58 (s, 3H, H-6′), 1.58–1.68 (m, 1H, H-1′’), 1.69 (s, 3H, H-5′), 2.50 (dtm, *J* = 15.0, 7.3 Hz, 1H, H-2′), 2.64 (dtm, *J* = 14.9, 5.9 Hz, 1H, H-2′), 5.14 (tm, *J* = 7.3 Hz, 1H, H-3′), 5.97 (d, *J* = 15.4 Hz, 1H, H-α), 6.04 (ddd, *J* = 7.1, 4.4, 0.8 Hz, 1H, H-1′), 6.48 (dd, *J* = 15.4, 10.1 Hz, 1H, H-β), 6.98 (d, *J* = 0.9 Hz, 1H, H-3), 7.18 (s, 2H, H-6, H-7), 12.43 (s, 1H, C5-O*H*), 12.59 (s, 1H, C8-O*H*); ^13^C-NMR (CDCl_3_): *δ* 8.9 (C-2′’, C-3′’), 14.6 (C-1′’), 18.0 (C-6′), 25.8 (C-5′), 32.9 (C-2′), 69.2 (C-1′), 111.6 (C-4a), 111.7 (C-8a), 117.3 (C-α), 117.9 (C-3′), 131.6 (C-3), 132.5 (C-7), 132.6 (C-6), 135.9 (C-4′), 148.8 (C-2), 155.8 (C-β), 165.3 (COO), 166.3 (C-5), 166.8 (C-8), 177.4 (C-1), 178.8 (C-4); MS (ESI^−^) m/z (%): 785.31 (9) [2(M-H)+Na]^−^, 382.14 (54) [M]^−^, 381.25 (100) [M-H]^−^; [M]^−^ calculated for C_22_H_22_O_6_: 382.1416.

*(R)-1-(1,4-Dihydro-5,8-dihydroxy-1,4-dioxonaphthalen-2-yl)-4-methylpent-3-enyl 3-cyclopropylpropanoate (**7**)*, 0.1 mmol Shikonin, 0.26 mmol DCC, 50 µmol DMAP and 0.15 mmol 3-cyclopropylpropanoic acid (**p2**) reaction time 15 h; CC on silica (8 g; hexanes/CH_2_Cl_2_ = 1:0 to hexanes/CH_2_Cl_2_ = 1:2); **7**, yield: 40%. **7**: R_f_ = 0.49 (silica, CH_2_Cl_2_); IR (ATR): 3080 (w), 2972 (w), ≈2950 (br) (OH), 2916 (w), 2857 (w), 1742 (m) (C=O), 1610 (s), 1569 (m), 1454 (m), 1228 (s), 1204 (s) (COC), 784 (m) cm^−1^; ^1^H-NMR (CDCl_3_): 0.04–0.09 (m, 2H, H-2′’, H-3′’), 0.40–0.49 (m, 2H, H-2′’, H-3′’), 0.66–0.77 (m, 1H, H-1′’), 1.54 (q, *J* = 7.3 Hz, 2H, H-β), 1.57 (s, 3H, H-6′), 1.68 (s, 3H, H-5′), 2.42–2.51 (m, 1H, 1H, H-2′), 2.48 (td, *J* = 7.5, 2.2 Hz, 2H, H-α), 2.61 (dtm, *J* = 15.2, 5.6 Hz, 1H, H-2′), 5.12 (tm, *J* = 7.4 Hz, 1H, H-3′), 6.02 (ddd, *J* = 7.2, 4.4, 0.8 Hz, 1H, H-1′), 6.98 (d, *J* = 1.0 Hz, 1H, H-3), 7.18 (s, 2H, H-6, H-7), 12.42 (s, 1H, C5-O*H*), 12.58 (s, 1H, C8-O*H*); ^13^C-NMR (CDCl_3_): *δ* 4.4, 4.5 (C-2′’, C-3′’), 10.4 (C-1′’), 17.9 (C-6′), 25.7 (C-5′), 30.0 (C-β), 32.9 (C-2′), 34.4 (C-α), 69.3 (C-1′), 111.6 (C-4a), 111.8 (C-8a), 117.8 (C-3′), 131.5 (C-3), 132.7 (C-7), 132.8 (C-6), 136.0 (C-4′), 148.4 (C-2), 166.8 (C-5), 167.4 (C-8), 172.4 (COO), 176.8 (C-1), 178.3 (C-4); MS (ESI^−^) m/z (%): 383.30 (100) [M-H]^−^, [M-H]^−^ calculated for C_22_H_24_O_6_: 383.4144.

*(R)-1-(1,4-Dihydro-5,8-dihydroxy-1,4-dioxonaphthalen-2-yl)-4-methylpent-3-enyl phenylacetate (**8**),* 0.1 mmol Shikonin, 0.15 mmol DCC, 25 µmol DMAP and two portions of 0.1 mmol phenylacetic acid each; reaction time 17 h; CC on silica (8 g; CH_2_Cl_2_); **8**, yield: 25%. **8**: R_f_ = 0.25 (silica, cyclohexane/CH_2_Cl_2_ = 1:4); ^1^H- and ^13^C-NMR data fit with literature values [23,38].

*(R)-1-(1,4-Dihydro-5,8-dihydroxy-1,4-dioxonaphthalen-2-yl)-4-methylpent-3-enyl cinnamate (**9**),* 0.1 mmol Shikonin, 0.15 mmol DCC, 25 µmol DMAP and 0.1 mmol cinnamic acid; reaction time 17 h; CC on silica (8 g; CH_2_Cl_2_); **9**, yield: 17%. **9**: R_f_ = 0.31 (silica, CH_2_Cl_2_); ^1^H-NMR data fit with literature values [26] and the ^13^C-NMR data fit with those of the corresponding alkannin derivative [25].

*(R)-1-(1,4-Dihydro-5,8-dihydroxy-1,4-dioxonaphthalen-2-yl)-4-methylpent-3-enyl 2-butynoate (**10**),* 0.1 mmol Shikonin, 0.20 mmol DCC, 25 µmol DMAP and 0.1 mmol 2-butynoic acid; reaction time 14 h; CC on silica (4 g; CH_2_Cl_2_); **10**, yield: 42%. **10**: R_f_ = 0.29 (silica, cyclohexane/CH_2_Cl_2_ = 1:4); IR (ATR): ≈ 3000 (vbr) (OH), 3052 (w), 2973 (w), 2928 (w), 2858 (w), 2238 (m) (C≡C), 1708 (s) (C=O), 1605 (s), 1568 (m), 1451 (m), 1238 (s) 1198 (s) (COC), 1066 (s), 768 (m), 738 (m) cm^−1^; ^1^H-NMR (CDCl_3_): 1.58 (s, 3H, H-6′), 1.70 (s, 3H, H-5′), 2.03 (s, 3H, H-γ), 2.51 (dtm, *J* = 15.0, 7.4 Hz, 1H, H-2′), 2.65 (dtm, *J* = 15.0, 6.0 Hz, 1H, H-2′), 5.12 (tm, *J* = 7.3 Hz, 1H, H-3′), 6.08 (ddd, *J* = 7.2, 4.6, 1.0 Hz, 1H, H-1′), 7.07 (d, *J* = 1.0 Hz, 1H, H-3), 7.18 (s, 2H, H-6, H-7), 12.42 (s, 1H, C5-O*H*), 12.57 (s, 1H, C8-O*H*); ^13^C-NMR (CDCl_3_): *δ* 3.9 (C-γ), 18.0 (C-6′), 25.7 (C-5′), 32.7 (C-2′), 70.3 (C-1′), 72.4 (C-α), 87.0 (C-β), 111.6 (C-4a), 111.8 (C-8a), 117.8 (C-3′), 131.5 (C-3), 133.0 (C-7), 133.2 (C-6), 136.5 (C-4′), 147.1 (C-2), 152.4 (COO), 167.7 (C-5), 168.2 (C-8), 175.8 (C-1), 177.4 (C-4); MS (ESI^−^) m/z (%): 354.07 (36) [M]^−^, 353.15 (100) [M-H]^−^; [M]^−^ calculated for C_20_H_18_O_6_: 354.1103.

*(R)-1-(1,4-Dihydro-5,8-dihydroxy-1,4-dioxonaphthalen-2-yl)-4-methylpent-3-enyl 2-pentynoate (**11**),* 0.1 mmol Shikonin, 0.15 mmol DCC, 25 µmol DMAP and 0.1 mmol 2-pentynoic acid; reaction time 16 h; CC on silica (4 g; cyclohexane / CH_2_Cl_2_ = 1:4); **11**, yield: 35%. **11**: R_f_ = 0.38 (silica, cyclohexane/CH_2_Cl_2_ = 1:4); IR (ATR): ≈3052 (w), 2980 (w), 2970 (vbr) (OH), 2937 (w), 2858 (w), 2236 (m) (C≡C), 1710 (s) (C=O), 1609 (s), 1569 (m), 1453 (m), 1232 (s) 1200 (s) (COC), 1080 (s), 1052 (s), 777 (m), 750 (m) cm^−1^; ^1^H-NMR (CDCl_3_): 1.24 (t, *J* = 7.5 Hz, 3H, H-δ), 1.58 (s, 3H, H-6′), 1.70 (s, 3H, H-5′), 2.39 (q, *J* = 7.5 Hz, 2H, H-γ), 2.51 (dtm, *J* = 14.6, 7.5 Hz, 1H, H-2′), 2.65 (dtm, *J* = 15.0, 5.9 Hz, 1H, H-2′), 5.14 (tm, *J* = 7.3 Hz, 1H, H-3′), 6.08 (ddd, *J* = 7.1, 4.6, 0.9 Hz, 1H, H-1′), 7.07 (d, *J* = 0.9 Hz, 1H, H-3), 7.18 (s, 2H, H-6, H-7), 12.43 (s, 1H, C5-O*H*), 12.57 (s, 1H, C8-O*H*); ^13^C-NMR (CDCl_3_): *δ* 12.4 (C-δ), 12.5 (C-γ), 18.0 (C-6′), 25.8 (C-5′), 32.7 (C-2′), 70.8 (C-1′), 72.0 (C-α), 92.1 (C-β), 111.6 (C-4a), 111.8 (C-8a), 117.3 (C-3′), 131.6 (C-3), 132.9 (C-7), 133.1 (C-6), 136.5 (C-4′), 147.2 (C-2), 152.6 (COO), 167.6 (C-5), 168.1 (C-8), 175.9 (C-1), 177.5 (C-4); MS (ESI^−^) m/z (%): 368.18 (24) [M]^−^, 367.21 (100), [M]^−^ calculated for C_21_H_20_O_6_: 368.1260.

*(R)-1-(1,4-Dihydro-5,8-dihydroxy-1,4-dioxonaphthalen-2-yl)-4-methylpent-3-enyl 3-phenylpropynoate (**12**),* 50 µmol Shikonin, 0.12 mmol DCC, 15 µmol DMAP and 68 µmol phenylpropynoic acid; reaction time 19 h; PTLC on silica with cyclohexane/CH_2_Cl_2_ = 1:1 (twice developed); **12**, yield: 67%. **12**: R_f_ = 0.42 (silica, cyclohexane/CH_2_Cl_2_ = 1:4); IR (ATR): 3058 (w), 2971 (w), 2915 (w), 2857 (w), 2212 (m) (CΞC), 1711 (s) (C=O), 1608 (s), 1568 (m), 1452 (m), 1275 (s), 1238 (s) 1164 (s), 1164 (vs) (COC), 1111 (m), 755 (s), 687 (m) cm^−1^; ^1^H-NMR (CDCl_3_): 1.61 (s, 3H, H-6′), 1.72 (s, 3H, H-5′), 2.57 (dtm, *J* = 14.9, 7.4 Hz, 1H, H-2′), 2.70 (dtm, *J* = 14.8, 5.7 Hz, 1H, H-2′), 5.19 (tm, *J* = 7.3 Hz, 1H, H-3′), 6.17 (ddd, *J* = 7.3, 4.7, 1.0 Hz, 1H, H-1′), 7.14 (d, *J* = 0.9 Hz, 1H, H-3), 7.19 (s, 2H, H-6, H-7), 7.40 (tm, *J* = 7.4 Hz, 2H, H-3′’, H-5′’), 7.48 (tt, *J* = 7.5, 1.1 Hz, 1H, H-4′’), 7.63 (m, 2H, H-2′’, H-6′’), 12.43 (s, 1H, C5-O*H*), 12.59 (s, 1H, C8-O*H*); ^13^C-NMR (CDCl_3_): *δ* 18.0 (C-6′), 25.8 (C-5′), 32.8 (C-2′), 71.1 (C-1′), 80.2 (C-α), 87.6 (C-β), 111.6, (C-4a), 111.9 (C-8a), 117.2 (C-3′), 119.3 (C-1′’), 128.6 (C-3′’, C-5′’), 130.9 (C-4′’), 131.6 (C-3), 133.0 (C-7), 133.1 (C-2′’, C-3′’), 133.3 (C-6), 136.6 (C-4′), 147.0 (C-2), 152.8 (COO), 167.9 (C-5), 168.4 (C-8), 175.6 (C-1), 177.2 (C-4); MS (ESI^−^) m/z (%):416.18 (27) [M]^−^, 415.12 (100) [M-H]^−^, [M]^−^ calculated for C_25_H_20_O_6_: 416.1260.

*(R,E)-1-(1,4-Dihydro-5,8-dihydroxy-1,4-dioxonaphthalen-2-yl)-4-methylpent-3-enyl 4-oxo-4-(4-methylphenyl)-2-butenoate (**13**),* 50 µmol Shikonin, 0.15 mmol DCC, 15 µmol DMAP and 95 µmol (E) 4-(4-methylphenyl)-3-oxo-2-butenoic acid; reaction time 16 h; PTLC on silica with cyclohexane/CH_2_Cl_2_ = 2:1 (four times developed); **13**, yield: 4%. **13**: R_f_ = 0.51 (silica, CH_2_Cl_2_); IR (ATR): 2967 (w), 2918 (m), ≈2950 (br) (OH), 2851 (w), 1728 (m) (C=O), 1669 (m), 1607 (vs), 1570 (m), 1453 (m), 1297 (s) (C-O-C), 1205 (m), 1161 (m), 756 (w) cm^−1^; ^1^H-NMR (CDCl_3_): 1.61 (s, 3H, H-6′), 1.70 (s, 3H, H-5′), 2.45 (s, 3H, Ph-C*H*_3_), 2.58 (dtm, *J* = 15.2, 7.6 Hz, 1H, H-2′), 2.70 (dtm, *J* = 15.2, 5.4 Hz, 1H, H-2′), 5.16 (tm, *J* = 7.2 Hz, 1H, H-3′), 6.17 (dd, *J* = 7.3, 4.7 Hz, 1H, H-1′), 6.95 (d, *J* = 15.5 Hz, 1H, H-α), 7.04 (d, *J* = 0.8 Hz, 1H, H-3), 7.19 (s, 2H, H-6, H-7), 7.32 (d, *J* = 8.2 Hz, 2H, H-3′’, H-5′’), 7.91 (d, *J* = 8.2 Hz, 2H, H-2′’, H-6′’), 7.96 (d, *J* = 15.5 Hz, 1H, H-β), 12.41 (s, 1H, C5-O*H*), 12.61 (s, 1H, C8-O*H*); ^13^C-NMR (CDCl_3_): *δ* 18.0 (C-6′), 21.8 (Ph-*C*H_3_), 25.8 (C-5′), 32.9 (C-2′), 70.5 (C-1′), 111.7 (C-4a), 111.8 (C-8a), 117.4 (C-3′), 129.0 (C-2′’, C-6′’), 129.7 (C-3′’, C-5′’), 131.2 (C-α), 131.3 (C-3), 133.1 (C-7), 133.3 (C-6), 134.0 (C-4′’), 136.5 (C-4′), 137.8 (C-β), 145.2 (C-1′’), 147.2 (C-2), 164.5 (COO), 168.0 (C-5), 168.6 (C-8), 175.5 (C-1), 177.0 (C-4), 188.6 (C-γ); MS (ESI^−^) m/z (%): 1417.45 (100) [3M-2H+K]^−^, 1401.56 (91) [3M-2H+Na]^−^, 1001.40 (55), 269.13 (42) [M-H-RCOOH]^−^; [3M-2H+K]^−^ calculated for C_27_H_24_O_7_: 1417.4047.

Synthesis and spectroscopic data of this compound were published mislabeled as *Z*-isomer. ^13^C-NMR shift values fit with our data, but ^1^H-NMR data and assignment of ^13^C-NMR signals are deficient [34].

*(R)-1-(1,4-Dihydro-5,8-dihydroxy-1,4-dioxonaphthalen-2-yl)-4-methylpent-3-enyl 2-oxopropanoate (**14**),* 0.25 mmol Shikonin, 0.75 mmol and 0.20 mmol DCC, 62 µmol DMAP and 0.25 mmol and 0.68 mmol pyruvic acid; reaction time 240 min plus 90 min; flash filtration on silica (10 g; cyclohexane/CH_2_Cl_2_ = 2:1 to 0:1); **14**, from the acrylester derived from HBr elimination. **14**, yield: 44%. **14**: R_f_ = 0.31 (silica, CH_2_Cl_2_); IR (ATR): 2969 (w), ≈2950 (br) (OH), 2928 (w), 2856 (w), 1733 (s) (C=O), 1607 (s), 1569 (m), 1435 (m), 1411 (m), 1262 (m), 1231 (s), 1202 (s) (COC), 1113 (m), 779 (m) cm^−1^; ^1^H-NMR (CDCl_3_): 1.59 (s, 3H, H-6′), 1.69 (s, 3H, H-5′), 2.50 (s, 3H, H-β), 2.61 (dtm, *J* = 14.9, 7.4 Hz, 1H, H-2′), 2.70 (dtm, *J* = 15.0, 5.9 Hz, 1H, H-2′), 5.13 (tm, *J* = 7.1 Hz, 1H, H-3′), 6.15 (dd, *J* = 7.1, 5.1 Hz, 1H, H-1′), 7.09 (s, 1H, H-3), 7.17 (s, 2H, H-6 and H-7), 12.38 (s, 1H, C5-O*H*), 12.58 (s, 1H, C8-O*H*); ^13^C-NMR (CDCl_3_): *δ* 17.9 (C-6′), 25.7 (C-5′), 26.7 (C-2′), 32.8 (C-β), 70.3 (C-1′), 71.5 (C-2′’), 111.6, (C-4a), 111.8 (C-8a), 116.9 (C-3′), 131.5 (C-3), 133.5 (C-7), 133.7 (C-6), 136.9 (C-4′), 145.9 (C-2), 159.6 (COO), 169.2 (C-5), 169.7 (C-8), 174.1 (C-1), 175.7 (C-4), 190.9 (C-α); MS (ESI^−^) m/z (%): 1111.26 (100) [3M-2H+K]^−^, 269.25 (40) [M-H-RCOOH]^−^, [3M-2H+K]^−^ calculated for C_19_H_18_O_7_: 1111.2638.

*(R)-1-(1,4-Dihydro-5,8-dihydroxy-1,4-dioxonaphthalen-2-yl)-4-methylpent-3-enyl 2-oxo-phenylacetate (**15**),* 50 µmol Shikonin, 0.12 mmol DCC, 15 µmol DMAP and 67 µmol 2-oxo-phenylacetic acid; reaction time 19 h; PTLC on silica with cyclohexane/CH_2_Cl_2_ = 1:1 (twice developed); **15**, yield: 57%. **15**: R_f_ = 0.33 (silica, cyclohexane/CH_2_Cl_2_ = 1:4); IR (ATR): 3064 (w), 2972(w), 2930 (w) 2857 (w), 1742 (m) (C=O), 1688 (m) (C=O), 1609 (s), 1570 (m), 1451 (m), 1193 (s) 1173 (s) (COC), 982 (m), 682 (m) cm^−1^; ^1^H-NMR (CDCl_3_): 1.59 (s, 3H, H-6′), 1.72 (s, 3H, H-5′), 2.61 (dtm, *J* = 15.1, 7.7 Hz, 1H, H-2′), 2.76 (dtm, *J* = 15.1, 5.5 Hz, 1H, H-2′), 5.20 (tm, *J* = 7.3 Hz, 1H, H-3′), 6.37 (ddd, J = 7.1, 4.3, 0.8 Hz, 1H, H-1′), 7.18 (d, *J* = 0.8 Hz, 1H, H-3), 7.19 (s, 2H, H-6, H-7), 7.53 (tm, J = 7.8 Hz, 2H, H-3′’, H-5′’), 7.69 (tt, *J* = 7.7, 1.2 Hz, 1H, H-4′’), 8.00 (m, 2H, H-2′’, H-6′’), 12.41 (s, 1H, C5-OH), 12.62 (s, 1H, C8-OH); ^13^C-NMR (CDCl_3_): δ 18.0 (C-6′), 25.8 (C-5′), 33.0 (C-2′), 71.2 (C-1′), 111.6, (C-4a), 111.8 (C-8a), 117.2 (C-3′), 129.0 (C-3′’, C-5′’), 130.0 (C-2′’, C-3′’), 131.4 (C-3), 132.2 (C-1′’), 133.4 (C-7), 133.8 (C-6), 135.1 (C-4′’), 137.0 (C-4′), 146.0 (C-2), 162.6 (COO), 169.1 (C-5), 169.6 (C-8), 174.3 (C-1), 175.9 (C-4), 185.6 (C-α); MS (ESI^−^) m/z (%): 1297.25 (100) [3M-2H+K]^−^, 1281.41 (85) [3M-2H+Na]^−^, 269.18 (22) [M-H-RCOOH]^−^, [3M-2H+K]^−^ calculated for C_24_H_20_O_7_: 1297.3108.

*(R)-1-(1,4-Dihydro-5,8-dihydroxy-1,4-dioxonaphthalen-2-yl)-4-methylpent-3-enyl 2-(2-oxocyclopentyl)acetate (**16**),* 0.1 mmol Shikonin, 0.25 mmol DCC, 25 µmol DMAP and 0.18 mmol 2-(2-oxocyclopentyl)acetic acid; reaction time 15 h; CC on silica (4 g; cyclohexane/CH_2_Cl_2_ = 1:0 to cyclohexane/CH_2_Cl_2_ = 0:1); PTLC on silica (developed seven times with cyclohexane/CH_2_Cl_2_ = 2:1); **16**, yield: 33%. **16**: R_f_ = 0.11 (silica, CH_2_Cl_2_); IR (ATR): 2966 (w), ≈2950 (br) (OH), 2917 (w), 2878 (w), 1736 (m) (C=O), 1609 (s), 1569 (m), 1452 (m), 1261(m), 1231 (m), 1202 (m) (COC), 1160 (m), 782 (m) cm^−1^; ^1^H-NMR (CDCl_3_): 1.58 (s, 6H, H-6′), 1.58-1.68 (m, 2H, H-5′’), 1.69 (s, 6H, H-5′), 1.74-1.88 (m, 2H, H-4′’), 2.01–2.10 (m, 2H, H-4′’), 2.11–2.23 (m, 2H, H-3′’), 2.25–2.41 (m, 4H, H-5′’, H-4′’), 2.41–2.56 (m, 6H, H-2′, H-α, H-1′’), 2.57–2.67 (m, 2H, H-2′), 7.74–2.88 (m, 2H, H-α), 5.07–5.15 (m, 2H, H-3′), 6.00–6.07 (m, 2H, H-1′), 7.00 (d, *J* = 1.6 Hz, 1H, H-3), 7.03 (d, *J* = 0.8 Hz, 1H, H-3), 7.18 (s, 4H, H-6, H-7), 12.42 (s, 1H, C5-O*H*), 12.43 (s, 1H, C5-O*H*), 12.58 (s, 2H, C8-O*H*); ^13^C-NMR (CDCl_3_): *δ* 18.0 (C-6′), 20.6 (C-2′’), 25.7 (C-5′), 29.2, 29.3 (C-5′’), 32.8, 32.9 (C-2′), 33.9, 34.0 (C-α), 2 × 37.3 (C-3′’), 45.6 (C-1′’), 2 × 69.8 (C-1′), 111.6 (C-4a), 111.9 (C-8a), 117.7 (C-3′), 2x 131.5 (C-3), 2x 132.8 (C-7), 2 × 133.0 (C-6), 136.1, 136.2 (C-4′), 147.9 (C-2), 167.3, 167.5 (C-5), 167.7, 167.8 (C-8), 171.0 (COO), 176.3, 176.3 (C-1), 177.8, 177.9 (C-4), 218.8 (C-2′’); MS (ESI^−^) m/z (5): 411.27 (100) [M-H]^−^, [M-H]^−^ calculated for C_23_H_24_O_7_: 411.1444.

*(R)-1-(1,4-Dihydro-5,8-dihydroxy-1,4-dioxonaphthalen-2-yl)-4-methylpent-3-enyl methyl butanedioate (**17**),* 50 µmol Shikonin, 0.12 mmol DCC, 12.5 µmol DMAP and 76 µmol monomethyl succinate (4-methoxy-4-oxobutanoic acid); reaction time 18 h; CC on silica (8 g; cyclohexane/CH_2_Cl_2_ = 2:1 to 0:1); **17**, yield: 15 %. **17**: R_f_ = 0.36 (silica, CH_2_Cl_2_); IR (ATR): 2961 (m), 2918 (w), 2855 (w), 1735 (m) (C=O), 1607 (s), 1568 (m), 1452 (m), 1436 (m), 1410 (m), 1345 (m), 1261 (m), 1229 (m), 1200 (s) (COC), 1150 (s), 1111 (m), 1020 (m), 781 (m) cm^−1^; ^1^H-NMR (CDCl_3_): 1.58 (s, 3H, H-6′), 1.69 (s, 3H, H-5′), 2.49 (dtm, *J* = 14.9, 7.3 Hz, 1H, H-2′), 2.58-2.68 (m, 3H, H-2′, H-α*), 2.70–2.75 (m, 2H, H-β*), 3.70 (s, 3H, OC*H*_3_), 5.12 (tm, *J* = 7.3 Hz, 1H, H-3′), 6.04 (dd, *J* = 7.1, 4.6 Hz, 1H, H-1′), 7.01 (d, *J* = 0.8 Hz, 1H, H-3), 7.18 (s, 2H, H-6, H-7), 12.42 (s, 1H, C5-O*H*), 12.57 (s, 1H, C8-O*H*); ^13^C-NMR (CDCl_3_): *δ* 17.9 (C-6′), 25.7 (C-5′), 28.8, 29.2 (C-α, C-β), 32.8 (C-2′), 51.9 (O*C*H_3_), 69.9 (C-1′), 111.6, (C-4a), 111.8 (C-8a), 117.6 (C-3′), 131.5 (C-3), 132.8 (C-7), 132.9 (C-6), 136.2 (C-4′), 147.9 (C-2), 167.2 (C-5), 167.7 (C-8), 171.1 (C-1′’), 172.4 (*C*OOCH_3_), 176.4 (C-1), 177.9 (C-4); MS (ESI^−^) m/z (%): 402.10 (28) [M]^−^, 401.15 (100) [M-H]^−^, [M]^−^ calculated for C_21_H_22_O_8_: 402.1315.

*(R)-1-(1,4-Dihydro-5,8-dihydroxy-1,4-dioxonaphthalen-2-yl)-4-methylpent-3-enyl ethyl (2E)-but-2-enedioate (**18**),* 0.1 mmol Shikonin, 0.15 mmol DCC, 20 µmol DMAP and 0.11 mmol monoethyl fumarate ((*E*)-4-methoxy-4-oxobut-2-enoic acid); reaction time 17 h; CC on silica (4 g; CH_2_Cl_2_); **18**, yield: 7%. **18**: R_f_ = 0.24 (silica, CH_2_Cl_2_); IR (ATR): 3074 (w), 2980 (m), ≈2950 (br) (OH), 2930 (w), 2857 (w), 1719 (s) (C=O), 1608 (s), 1569 (m), 1452 (m), 1294 (s), 1256 (s), 1230 (s), 1202 (s) (COC), 1149 (s), 1112 (m), 1026 (m), 771 (m) cm^−1^; ^1^H-NMR (CDCl_3_): 1.35 (t, *J* = 7.1 Hz, O-CH_2_-C*H*_3_), 1.57 (s, 3H, H-6′), 1.69 (s, 3H, H-5′), 2.54 (dtm, *J* = 15.0, 7.3 Hz, 1H, H-2′), 2.67 (dtm, *J* = 15.0, 5.0 Hz, 1H, H-2′), 4.29 (q, *J* = 7.1 Hz, O-C*H*_2_-CH_3_), 5.13 (tm, *J* = 7.3 Hz, 1H, H-1′), 6.17 (ddd, *J* = 7.3, 4.6, 0.8 Hz, 1H, H-1′), 6.90, 6.92 (2d, *J* = 15.9 Hz, 2H, H-α, H-β), 7.00 (d, *J* = 0.8 Hz, 1H, H-3), 7.19 (s, 2H, H-6, H-7), 12.41 (s, 1H, C5-O*H*), 12.59 (s, 1H, C8-O*H*); ^13^C-NMR (CDCl_3_): *δ* 14.1 (O-CH_2_-*C*H_3_), 18.0 (C-6′), 25.8 (C-5′), 32.8 (C-2′), 61.5 (O-*C*H_2_-CH_3_), 70.5 (C-1′), 111.6 (C-4a), 111.8 (C-8a), 117.3 (C-3′), 131.2 (C-3), 132.7 (C-α or C-β), 133.2 (C-7), 133.4 (C-6), 134.8 (C-α or C-β), 136.6 (C-4′), 147.1 (C-2), 163.7 (C-α-*C*OO), 164.7 (*C*OOEt), 168.2 (C-5), 168.7 (C-8), 175.3 (C-1), 176.8 (C-4); MS (ESI^−^) m/z (%):1279 (5) [3M-2H+K]^−^, 1265 (8) [3M-2H+Na]^−^, 1264 (14) [3M-2H+Na]^−^, 1263 (19) [3M-2H+Na]^−^, 414.14 (28) [M]^−^, 413.21 (100) [M-H]^−^; [M]^−^ calculated for C_22_H_22_O_8_: 414.1315.

*(R)-1-(1,4-Dihydro-5,8-dihydroxy-1,4-dioxonaphthalen-2-yl)-4-methylpent-3-enyl 2-methylpropanoate (**19**),* 0.1 mmol Shikonin, 0.20 mmol DCC, 25 µmol DMAP and 0.11 mmol isobutyric acid; reaction time 22 h; PTLC on silica (1 mm layer; developed once with cyclohexane/CH_2_Cl_2_ = 1:4); **19**, yield: 14%. **19**: R_f_ = 0.24 (silica, cyclohexane/CH_2_Cl_2_ = 1:4); ^1^H- and ^13^C-NMR data fit with literature values [62,63,64].

*(R)-1-(1,4-Dihydro-5,8-dihydroxy-1,4-dioxonaphthalen-2-yl)-4-methylpent-3-enyl 3-methyl-butanoate (**20**),* 0.1 mmol Shikonin, 0.15 mmol DCC, 25 µmol DMAP and 0.1 mmol isovaleric acid; reaction time 17 h; PTLC on silica (developed three times with cyclohexane/CH_2_Cl_2_ = 2:5); **20**, yield: 11 %. **20**: R_f_ = 0.29 (silica, cyclohexane/CH_2_Cl_2_ = 1:4); ^1^H- and ^13^C-NMR data fit with literature values [62,64].

*(R)-1-(1,4-Dihydro-5,8-dihydroxy-1,4-dioxonaphthalen-2-yl)-4-methylpent-3-enyl (E)-2-butenoate (**21**),* 0.1 mmol Shikonin, 0.15 mmol DCC, 25 µmol DMAP and two portions of 0.1 mmol crotonoic acid each; reaction time 16 plus 24 h; CC on silica (4 g; cyclohexane/CH_2_Cl_2_ = 1:1 to 0:1); **21**, yield: 17%. **21**: R_f_ = 0.27 (silica, cyclohexane/ CH_2_Cl_2_ = 1:4); ^1^H-NMR (CDCl_3_): 1.58 (s, 3H, H-6′), 1.69 (s, 3H, H-5′), 1.93 (dd, *J* = 6.8 Hz, 1.9, 3H, H-γ), 2.50 (dtm, *J* = 14.9, 7.2 Hz, 1H, H-2′), 2.64 (dtm, *J* = 15.1, 5.5 Hz, 1H, H-2′), 5.14 (tquint, *J* = 7.3, 1.3 Hz, 1H, H-3′), 5.91 (dq, *J* = 15.5, 1.5 Hz 2H, H-α), 6.07 (ddd, *J* = 6.2, 3.7, 0.8 Hz, 1H, H-1′), 6.98 (d, *J* = 1.0 Hz, 1H, H-3), 7.05 (dq, *J* = 15.5, 6.9 Hz, 1H, H-β), 7.18 (s, 2H, H-6, H-7), 12.43 (s, 1H, C5-O*H*), 12.59 (s, 1H, C8-O*H*); ^13^C-NMR (CDCl_3_): *δ* 17.9, 18.1 (C-γ, C-6′), 25.8 (C-5′), 29.7 (C-β), 32.9 (C-2′), 69.3 (C-1′), 111.6 (C-4a), 111.8 (C-8a), 117.8 (C-3′), 122.1 (C-α), 131.5 (C-3), 132.6, 132.7 (C-7, C-6), 136.0 (C-4′), 146.1 (C-ß), 148.5 (C-2), 165.1 (COO), 166.6 (C-5), 167.1 (C-8), 177.1 (C-1), 178.6 (C-4); ^1^H-NMR shift values fit with literature values [39].

*(R,E,E)-1-(1,4-Dihydro-5,8-dihydroxy-1,4-dioxonaphthalen-2-yl)-4-methylpent-3-enyl 2,4-hexadienoate (**22**),* 0.1 mmol Shikonin, 0.35 mmol DCC, 37.5 µmol DMAP and 0.1 mmol sorbic acid each; reaction time 17 h; PTLC on silica four times developed with cyclohexane/CH_2_Cl_2_ = 1:1 and once with CH_2_Cl_2_; **22**, yield: 8%. **22**: R_f_ = 0.40 (silica, CH_2_Cl_2_). ^1^H- and ^13^C-NMR data fit with literature values [23,38].

*(R)-1-(1,4-Dihydro-5,8-dihydroxy-1,4-dioxonaphthalen-2-yl)-4-methylpent-3-enyl benzoate (**23**),* 0.1 mmol Shikonin, 0.15 mmol DCC, 25 µmol DMAP and 0.1 mmol benzoic acid each; reaction time 17 h; PTLC on silica (twice developed with cyclohexane/CH_2_Cl_2_ = 1:1 and twice developed with cyclohexane/CH_2_Cl_2_ = 1:2); **23**, yield: 5%. **23**: R_f_ = 0.27 (silica, cyclohexane/CH_2_Cl_2_ = 1:4); ^1^H- and ^13^C-NMR data fit with literature values [23,38].

*(R)-1-(1,4-Dihydro-5,8-dihydroxy-1,4-dioxonaphthalen-2-yl)-4-methylpent-3-enyl diphenylacetate (**24**)*, 50 µmol Shikonin, 0.12 mmol DCC, 15 µmol DMAP and 61 µmol diphenylacetic acid each; reaction time 13 h; CC on silica (8 g; cyclohexane/CH_2_Cl_2_ = 1:1 to 4:1); PTLC on silica with cyclohexane/CH_2_Cl_2_ = 1:1 (three times developed); PTLC on silica with cyclohexane/CH_2_Cl_2_ = 2:1 (three times developed); **24**, yield: 29%. **24**: R_f_ = 0.69 (silica, CH_2_Cl_2_);

^1^H- and ^13^C-NMR data fit with literature values [23,38].

*(R,E)-1-(1,4-Dihydro-5,8-dihydroxy-1,4-dioxonaphthalen-2-yl)-4-methylpent-3-enyl 3-phenyl-propanoate (**25**),* 50 µmol Shikonin, 0.12 mmol DCC, 20 µmol DMAP and 73 µmol 3-phenylpropanoic acid; reaction time 15 h; PTLC on silica with cyclohexane/CH_2_Cl_2_ = 1:1 (three times developed); **25**, yield: 14%. **25**: R_f_ = 0.45 (silica, cyclohexane/CH_2_Cl_2_ = 1:4); IR (ATR): 3028 (vw), 2965 (w), 2918 (w), ≈2950 (br) (OH), 2851 (w), 1738 (s) (C=O), 1608 (s), 1568 (m), 1453 (m), 1342 (m), 1264 (m), 1229 (m), 1201 (s) (C-O-C), 1146 (m), 1112 (m), 735 (m), 698 (m) cm^−1^; ^1^H- and ^13^C-NMR data fit with literature values [36].

*(R,E)-1-(1,4-Dihydro-5,8-dihydroxy-1,4-dioxonaphthalen-2-yl)-4-methylpent-3-enyl 3-(4-dimethylaminophenyl)2-propenoate (**26**),* 0.5 mmol Shikonin, 1.5 mmol DCC, 0.125 mmol DMAP and 0.675 mmol *p*-dimethylaminocinnamic acid; reaction time 24 h; flash CC on silica with cyclohexane/CH_2_Cl_2_ = 2:1 to 0:1; **26**, yield: 6%. **26**: R_f_ = 0.13 (silica, CH_2_Cl_2_). IR (ATR): 2920 (m, br), 2852 (w), 1707 (m), (C=O), 1597 (vs), 1526 (m), 1444 (m), 1232 (m), 1206 (m), 1182 (m), 1146 (s) (COC), 1113 (m), 815 (w) cm^−1^; ^1^H-NMR (CDCl_3_): 1.60 (d, *J* = 0.8 Hz, 3H, H-6′), 1.69 (d, *J* = 0.8 Hz, 3H, H-5′), 2.56 (dtm, *J* = 15.0, 7.1 Hz, 1H, H-2′), 2.68 (dtm, *J* = 15.0, 5.7 Hz, 1H, H-2′), 3.06 (s, 6H, NC*H*_3_), 5.19 (tm, *J* = 7.2 Hz, 1H, H-3′), 6.12 (ddd, *J* = 7.0, 4.4, 0.7 Hz, 1H, H-1′), 6.31 (d, *J* = 15.8 Hz, 1H, H-α), 6.83 (d, *J* = 8.0 Hz, 1H, H-3′’), 7.04 (d, *J* = 1.0 Hz, 1H, H-3), 7.19 (s, 2H, H-6 and H-7), 7.47 (d, *J* = 8.6 Hz, 1H, H-3′’), 7.67 (d, *J* = 15.8 Hz, 1H, H-β), 12.43 (s, 1H, C5-O*H*), 12.61 (s, 1H, C8-O*H*); ^13^C-NMR (CDCl_3_): *δ* 18.0 (C-6′), 25.8 (C-5′), 32.9 (C-2′), 40.9 (N*C*H_3_), 69.2 (C-1′), 111.6 (C-4a), 111.9 (C-8a), 112.4 (C-α), 112.9 (C-3′’, C-5′’), 117.9 (C-3′), 123.6 (C-3′’), 129.9 (C-2′’, C-6′’), 131.7 (C-3), 132.4 (C-7), 132.5 (C-6), 135.8 (C-4′), 146.2 (C-β), 148.8 (C-2), 166.2 (C-8), 166.3 (COO), 166.8 (C-5), 177.5 (C-4), 178.9 (C-1); MS no spectra available via (ESI^−^) or (ESI^+^), [M]^−^ calculated for C_27_H_27_NO_6_: 461.1838.

*(R)-1-(1,4-Dihydro-5,8-dihydroxy-1,4-dioxonaphthalen-2-yl)-4-methylpent-3-enyl pyridine-3-carboxylate (**27**),* 50 µmol Shikonin, 0.12 mmol DCC, 55 µmol DMAP and 85 µmol nicotinic acid; reaction time 15 h; PTLC on silica with CH_2_Cl_2_ (three times developed) and PTLC on silica with CH_2_Cl_2_ (three times developed); **27**, yield: 10%. **27**: R_f_ = 0.50 (silica, CH_2_Cl_2_/MeOH = 40:1); ^1^H-NMR data fit with literature values [27].

*(R)-1-(1,4-Dihydro-5,8-dihydroxy-1,4-dioxonaphthalen-2-yl)-4-methylpent-3-enyl pyridine-4-carboxylate (**28**),* 50 µmol Shikonin, 100 µmol DCC, 7.5 µmol DMAP and 75 µmol isonicotinic acid; reaction time 22 h; flash CC on silica (8 g; CH_2_Cl_2_ to CH_2_Cl_2_/MeOH = 40:1), PTLC on silica with CH_2_Cl_2_ (three times developed) and PTLC on silica with cyclohexane/CH_2_Cl_2_ = 1:4 (four times developed); **28**, yield: 1%. **28**: R_f_ = 0.41 (silica, CH_2_Cl_2_ / MeOH = 40:1); IR (ATR): 2961 (w), 2918 (m), ≈2950 (br) (OH), 2851 (w), 1734 (s) (C=O), 1610 (s), 1570 (m), 1559 (m), 1456 (m), 1406 (m), 1269 (s) (C-O-C), 1205 (m), 1117 (m), 757 (w) cm^−1^; ^1^H-NMR (CDCl_3_): 1.62 (s, 3H, H-6′), 1.69 (s, 3H, H-5′), 2.68 (dtm, J = 15.2, 7.2 Hz, 1H, H-2′), 2.78 (dtm, J = 15.0, 5.6 Hz, 1H, H-2′), 5.18 (tm, J = 7.3 Hz, 1H, H-3′), 6.30 (ddd, J = 7.2, 4.8, 0.7 Hz, 1H, H-1′), 7.06 (d, J = 0.9 Hz, 1H, H-3), 7.19 (s, 2H, H-6 and H-7), 8.02 (s, 2H, H-3′’ and H-5′’), 8.87 (d, J = 7.3 Hz, 2H, H-2′’ and H-6′’), 12.38 (s, 1H, C5-OH), 12.63 (s, 1H, C8-OH); ^13^C-NMR (CDCl_3_ / MeOD = 2:1): δ 17.6 (C-6′), 25.8 (C-5′), 32.6 (C-2′), 70.9 (C-1′), 111.4, (C-4a), 111.8 (C-8a), 117.1 (C-3′), 123.0 (C-3′’, C-5′’), 130.6 (C-3), 133.2, 133.4 (C-6, C-7), 136.5 (C-4′), 137.2 (C-4′’), 146.4 (C-2), 150.0 (C-2′’, C-6′’), 163.6 (COO), 168.7 (C-8), 169.1 (C-5), 173.8 (C-1), 175.3 (C-4); MS (ESI^−^) m/z (%): 807 (14) [2(M-H)+Na], 393.09 (28) [M]^−^, 392.07 (100) [M-H]^−^; [M]^−^ calculated for C_22_H_19_NO_6_: 393.1212.

*(R)-1-(1,4-Dihydro-5,8-dihydroxy-1,4-dioxonaphthalen-2-yl)-4-methylpent-3-enyl 3-(1*H*-indol-3-yl)-propanoate (**29**),* 50 µmol Shikonin, 0.12 mmol DCC, 15 µmol DMAP and 63 µmol 3-(1*H*-indol-3-yl)-propanoic acid; reaction time 19 h; PTLC on silica CH_2_Cl_2_ (twice developed); **29**, yield: 22%. **29**: R_f_ = 0.22 (silica, CH_2_Cl_2_); ^1^H-NMR (CDCl_3_): 1.53 (s, 3H, H-6′), 1.64 (s, 3H, H-5′), 2.41 (dtm, *J* = 14.9, 7.5 Hz, 1H, H-2′), 2.54 (dtm, *J* = 14.7, 5.7 Hz, 1H, H-2′), 2.77-2.83 (m, 2H, H-α), 3.13 (tm, *J* = 7.4 Hz, 2H, H-β), 5.03 (tquint, *J* = 7.2, 1.1 Hz, 1H, H-3′), 5.99 (ddd, *J* = 6.9, 4.8, 0.7 Hz, 1H, H-1′), 6.70 (d, *J* = 0.7 Hz, 1H, H-3), 6.99 (d, *J* = 2.1 Hz, 1H, H-2′’), 7.11 (td, *J* = 7.4 Hz, 0.8 Hz, 1H, H-6′’), 7.16 (td, *J* = 7.8 Hz, 1.3 Hz, 1H, H-7′’), 7.18 (s, 2H, H-6, H-7), 7.34 (d, *J* = 7.3 Hz, 1H, H-8′’), 7.59 (d, *J* = 7.7 Hz, 1H, H-5′’), 8.00 (s br, 1H, NH), 12.43 (s, 1H, C5-O*H*), 12.56 (s, 1H, C8-O*H*); ^1^H-NMR data fit with literature values (Wang et al., 2014). ^13^C-NMR (CDCl_3_): *δ* 17.9 (C-6′), 20.7 (C-β), 25.7 (C-5′), 32.8 (C-2′), 35.0 (C-α), 69.4 (C-1′), 111.2 (C-8′’), 111.6 (C-4a), 111.8 (C-8a), 114.6 (C-3‘’), 117.6 (C-3′), 118.6 (C-5′’), 119.4 (C-6′’), 121.5 (C-2′’), 122.1 (C-7′’), 127.1 (C-4′’), 131.5 (C-3), 132.5 (C-7), 132.7 (C-6), 136.0 (C-4′), 136.3 (C-9‘‘), 148.3 (C-2), 166.5 (C-5), 167.0 (C-8), 172.3 (COO), 177.1 (C-1), 178.6 (C-4).

*(R,E)-1-(1,4-Dihydro-5,8-dihydroxy-1,4-dioxonaphthalen-2-yl)-4-methylpent-3-enyl 3-(furan-2-yl)-2-propenoate (**30**),* 50 µmol Shikonin, 0.12 mmol DCC, 15 µmol DMAP and 72 µmol 3-(furan-2-yl)-2-propenoic acid; reaction time 13 h; PTLC on silica with cyclohexane/CH_2_Cl_2_ = 1:1 (three times developed); **30**, yield: 5%. **30**: R_f_ = 0.43 (silica, cyclohexane/CH_2_Cl_2_ = 1:4); IR (ATR): 2969 (w), 2918 (m, br), 2853 (w), 1713 (m), (C=O), 1637 (m), 1609 (s), 1569 (m), 1454 (m), 1263 (m), 1232 (m), 1204 (s), 1155 (s), (COC), 1017 (m), 752 (m) cm^−1^; ^1^H-NMR (CDCl_3_): 1.60 (d, *J* = 0.8 Hz, 3H, H-6′), 1.69 (s, 3H, H-5′), 2.55 (dtm, *J* = 14.9, 7.4 Hz, 1H, H-2′), 2.68 (dtm, *J* = 14.8, 5.7 Hz, 1H, H-2′), 5.17 (tm, *J* = 7.2 Hz, 1H, H-3′), 6.12 (ddd, *J* = 7.2, 4.4, 0.5 Hz, 1H, H-1′), 6.38 (d, *J* = 15.7 Hz, 1H, H-α), 6.50 (dd, *J* = 3.4, 1.8 Hz, 1H, H-4′’), 6.66 (d, *J* = 3.4 Hz, 1H, H-3′’), 7.03 (d, *J* = 0.6 Hz, 1H, H-3), 7.19 (s, 2H, H-6 and H-7), 7.46 (d, *J* = 15.7 Hz, 1H, H-β), 7.52 (d, *J* = 1.5 Hz, 1H, H-5‘’), 12.42 (s, 1H, C5-O*H*), 12.60 (s, 1H, C8-O*H*); ^13^C-NMR (CDCl_3_): *δ* 18.0 (C-6′), 25.8 (C-5′), 32.9 (C-2′), 69.6 (C-1′), 111.6 (C-4a), 111.9 (C-8a), 112.4 (C-4′’), 114.9 (C-α), 115.5 (C-3′’), 117.8 (C-3′), 131.6 (C-3), 132.0 (C-β), 132.6 (C-7), 132.8 (C-6), 136.1 (C-4′), 145.1 (C-5′’), 148.4 (C-2), 150.7 (C-2′’), 165.7 (COO), 166.8 (C-8), 167.3 (C-5), 177.0 (C-4), 178.4 (C-1); MS (ESI^−^) m/z (%): 837 (51) [2(M-H)+Na]^−^, 408.07 (41) [M]^−^, 407.20 (67) [M-H]^−^, 137 (100), [M]^−^ calculated for C_23_H_20_O_7_: 408.1209.

*(R)-1-(1,4-Dihydro-5,8-dihydroxy-1,4-dioxonaphthalen-2-yl)-4-methylpent-3-enyl bromoacetate (**31**),* 0.1 mmol Shikonin, 0.15 mmol DCC, 25 µmol DMAP and 0.1 mmol bromoacetic acid each; reaction time 17 h; CC on silica (8 g; CH_2_Cl_2_) and PTLC on silica with cyclohexane/CH_2_Cl_2_ = 2:1 (four times developed); **31**, yield: 12%. **31**: R_f_ = 0.33 (silica, CH_2_Cl_2_). ^1^H-NMR (CDCl_3_): 1.51 (s, 3H, H-6′), 1.70 (s, 3H, H-5′), 2.52 (dtm, *J* = 15.1, 7.5 Hz, 1H, H-2′), 2.66 (dtm, *J* = 15.1, 5.6 Hz, 1H, H-2′)), 3.87 (d, *J* = 12.1 Hz, 1H, C*H*_2_Br), 3.90 (d, *J* = 12.1 Hz, 1H, C*H*_2_Br), 5.13 (tquint, *J* = 7.3, 1.3 Hz, 1H, H-3′), 6.09 (ddd, *J* = 7.3, 4.5, 0.9 Hz, 1H, H-1′), 7.08 (d, *J* = 0.9 Hz, 1H, H-3), 7.18 (s, 2H, H-6, H-7), 12.41 (s, 1H, C5-O*H*), 12.57 (s, 1H, C8-O*H*); ^13^C-NMR (CDCl_3_): *δ* 18.0 (C-6′), 25.3 (*C*H_2_Br), 25.8 (C-5′), 32.8 (C-2′), 70.2 (C-1′), 111.6 (C-4a), 111.9 (C-8a), 117.2 (C-3′), 131.2 (C-3), 133.1 (C-7), 133.4 (C-6), 136.6 (C-4′), 146.8 (C-2), 166.1 (COO), 168.3 (C-5), 168.8 (C-8), 175.2 (C-1), 176.8 (C-4); disregarding the assignment of the signals the ^1^H-NMR spectrum fits with literature values [46].

*(R)-1-(1,4-Dihydro-5,8-dihydroxy-1,4-dioxonaphthalen-2-yl)-4-methylpent-3-enyl 3-bromopropanoate (**32**),* 0.3 mmol Shikonin, 16 mL CH_2_Cl_2_, two portions 0.45 mmol DCC, 75 µmol DMAP and two portions of 0.3 mmol 3-bromopropanoic acid; reaction time 17 h plus 4 days; CC on silica (10 g; CH_2_Cl_2_) and PTLC on silica with cyclohexane/CH_2_Cl_2_ = 2:1 (six times developed). It was impossible to separate **32**, from the acryl ester derived from HBr elimination. **32**, yield: 2%. **32**: R_f_ = 0.38 (silica, CH_2_Cl_2_); IR (ATR): 2972 (w), ≈2950 (br) (OH), 2926 (w), 2857 (w), 1742 (m) (C=O), 1608 (s), 1568 (m), 1435 (m), 1408 (m), 1228 (s), 1201 (s) (COC), 1112 (m), 774 (m) cm^−1^; ^1^H-NMR (CDCl_3_): 1.59 (s, 3H, H-6′), 1.69 (s, 3H, H-5′), 2.51 (dtm, *J* = 14.8, 7.2 Hz, 1H, H-2′), 2.64 (dtm, *J* = 14.9, 5.5 Hz, 1H, H-2′), 3.01 (t, *J* = 6.7 Hz, 2H, H-α), 3.60 (2t, *J* = 6.8 Hz 2H, H-β), 5.13 (tm, *J* = 7.3 Hz, 1H, H-3′), 6.09 (ddd, *J* = 7.3, 4.6, 0.9 Hz, 1H, H-1′), 7.04 (d, *J* = 1.0 Hz, 1H, H-3), 7.18 (s, 2H, H-6, H-7), 12.41 (s, 1H, C5-O*H*), 12.58 (s, 1H, C8-O*H*); ^13^C-NMR (CDCl_3_): *δ* 18.0 (C-6′), 25.5 (C-5′), 29.7 (C-β), 32.9 (C-2′), 37.7 (C-α), 70.2 (C-1′), 111.6 (C-4a), 111.8 (C-8a), 117.5 (C-3′), 131.5 (C-3), 133.0 (C-7), 133.2 (C-6), 136.3 (C-4′), 147.5 (C-2), 167.7 (C-5), 168.2 (C-8), 169.4 (COO), 175.8 (C-1), 177.4 (C-4); MS (ESI^−^) m/z (%): 269.12 (100) [M-RCOOH-H]^−^, 241.08 (61) [269-CO]; [M]^−^ calculated for C_19_H_19_BrO_6_: 422.0365 and 424.0345.

*(R)-1-(1,4-Dihydro-5,8-dihydroxy-1,4-dioxonaphthalen-2-yl)-4-methylpent-3-enyl 4-bromo-butanoate (**33**),* 0.1 mmol Shikonin, 0.25 mmol DCC, 50 µmol DMAP and 0.5 mmol 4-bromobutanoic acid; reaction time 5 days; CC on silica (10 g; cyclohexane/CH_2_Cl_2_ = 2:5); **33**, yield: 23%. **33**: R_f_ = 0.34 (silica, cyclohexane/CH_2_Cl_2_ = 1:4); IR (ATR): 3055 (w), 2967(w), 2920 (m) (OH), 2855 (w), 1738 (s) (C=O), 1607 (s), 1568 (m), 1453 (m), 1198 (s) (COC), 1112 (s), 775 (m) cm^−1^; ^1^H-NMR (CDCl_3_): 1.59 (s, 3H, H-6′), 1.70 (s, 3H, H-5′), 2.20 (quint, *J* = 6.8 Hz, 2H, H-β), 2.49 (dtm, *J* = 14.9, 7.0 Hz, 1H, H-2′), 2.57-2.67 (m, 3H, H-2′, H-α), 3.47 (t, *J* = 6.4 Hz, 2H, H-γ), 5.12 (tm, *J* = 7.3 Hz, 1H, H-3′), 6.05 (ddd, *J* = 7.4, 4.6, 0.9 Hz, 1H, H-1′), 7.00 (d, *J* = 1.0 Hz, 1H, H-3), 7.19 (s, 2H, H-6, H-7), 12.42 (s, 1H, C5-OH), 12.59 (s, 1H, C8-OH); ^13^C-NMR (CDCl_3_): δ 18.0 (C-6′), 25.8 (C-5′), 27.5 (C-β), 32.38, 32.44 (C-α, C-γ), 32.9 (C-2′), 69.6 (C-1′), 111.6 (C-4a), 111.8 (C-8a), 117.6 (C-3′), 131.3 (C-3), 132.9 (C-7), 133.1 (C-6), 136.3 (C-4′), 147.9 (C-2), 167.5 (C-5), 168.0 (C-8), 171.4 (COO), 176.1 (C-1), 177.6 (C-4); MS (ESI^−^) m/z (%): 438.11 (27) [M]^−^, 437.17 (83) [M-H]^−^, 436.36 (35) [M]^−^, 435.30 (100) [M-H]^−^; [M]^−^ calculated for C_20_H_21_BrO_6_: 436.0522 and 438.0501.

*(R)-1-(1,4-Dihydro-5,8-dihydroxy-1,4-dioxonaphthalen-2-yl)-4-methylpent-3-enyl 5-bromopentanoate (**34**),* 0.1 mmol Shikonin, 0.15 mmol DCC, 25 µmol DMAP and 0.1 mmol 5-brompentanoic acid each; reaction time 17 h; CC on silica (8 g; CH_2_Cl_2_) and PTLC on silica with cyclohexane/CH_2_Cl_2_ = 2:1 (four times developed); **34**, yield: 20%. **34**: R_f_ = 0.32 (silica, CH_2_Cl_2_); IR (ATR): 3055 (w), 2967(w), 2920 (m) (OH), 2855 (w), 1738 (s) (C=O), 1607 (s), 1568 (m), 1453 (m), 1198 (s) (COC), 1112 (s), 775 (m) cm^−1^; ^1^H-NMR (CDCl_3_): 1.59 (s, 3H, H-6′), 1.70 (s, 3H, H-5′), 1.77-1.86 (m, 2H, H-β), 1.87-1.96 (m, 2H, H-γ), 2.44 (td, *J* = 7.1, 1.7 Hz, 2H, H-α), 2.49 (dtm, *J* = 14.9, 7.4 Hz, 1H, H-2′), 2.61 (dtm, *J* = 14.7, 5.4 Hz, 1H, H-2′), 3.42 (t, *J* = 6.5 Hz, 2H, H-δ), 5.12 (tm, *J* = 7.3 Hz, 1H, H-3′), 6.05 (dd, *J* = 7.1, 6.1, Hz, 1H, H-1′), 6.99 (s, 1H, H-3), 7.18 (s, 2H, H-6 and H-7), 12.41 (s, 1H, C5-O*H*), 12.58 (s, 1H, C8-O*H*); ^13^C-NMR (CDCl_3_): *δ* 18.0 (C-6′), 23.4 (C-β), 25.7 (C-5′), 31.8 (C-γ), 32.8 (C-δ), 32.9 (C-2′), 33.3 (C-α), 69.5 (C-1′), 111.6 (C-4a), 111.8 (C-8a), 117.7 (C-3′), 131.3 (C-3), 132.9 (C-7), 133.0 (C-6), 136.1 (C-4′), 148.0 (C-2), 167.4 (C-5), 167.9 (C-8), 171.8 (COO), 176.2 (C-1), 177.7 (C-4); MS (ESI-) m/z (%): 452.04 (30) [M]^−^, 451.08 (100) [M-H]^−^, 450.16 (30) [M]^−^, 449.16 (98) [M-H]^−^; [M]^−^ calculated for C_21_H_23_BrO_6_: 450.0678 and 452.0658.

### 3.4. Synthesis of Precursor Acids

#### 3.4.1. 2-(1-Bicyclo[4.1.0]heptyl)acetic Acid (**p1**)

Under argon and at 0 °C, a solution of (1-cyclohexenyl)-2-acetic (226 mg, 1.61 mmol) in 1.3 mL toluene and and CH_2_I_2_ (1.06 g, 4.97 mmol) were added to a solution of ZnEt_2_ in toluene (3.2 mL, 1 M). After stirring overnight, the mixture was poured into HCl (2 M). The organic layer was separated, and the aqueous phase was extracted with Et_2_O. The combined organic layers were extracted with NaOH (1 M). The aqueous phase was acidified with 2 M HCl and extracted with CH_2_CI_2_. Evaporation of the solvent gave **p1** [65], which was used without further purification. Yield: 60 %. ^1^H- and ^13^C NMR data fit with literature values [66].

#### 3.4.2. 3-Cyclopropylpropanoic Acid (**p2**)

A solution of LDA (2. 5 mL, 2 M in THF/toluene/heptane) was added to THF (25 mL) under argon. The mixture was cooled to 0 °C and acetic acid (150 mg, 2.5 mmol) in THF (4 mL) were added dropwise. The mixture was then stirred for 30 min at 45 °C. After cooling to room temperature, bromomethylcyclopropane (405 mg, 3 mmol) was added within 15 min. The mixture was stirred at 45 °C for 5.5 h, cooled to room temperature, and poured into a mixture of Et_2_O and water (25 mL and 37 mL resp.). The organic layer was acidified with 2 N HCl and extracted AcOEt (3 × 25 mL). The extract was dried over Na_2_SO_4_ and most of the solvent was evaporated under reduced pressure resulting in a 74% solution of **p2** in THF (67 mg, yield: 23%). This solution was used without further purification.

^1^H-NMR (CDCl_3_): 0.06 (q, *J* = 4.9 Hz, 2H, cyclpropyl-C*H*_2_), 0.39–49 (m, 2H, cyclpropyl-C*H*_2_), 0.67–0.79 (m, 1H), 1.53 (q, *J* = 7.3 Hz, 2H), 2.45 (t, *J* = 7.4 Hz, 2H, C*H*_2_-COOH), 10.72 (br.s, COO*H*); data fit with literature [67]; ^13^C NMR (CDCl_3_), δ: 4.45 (cyclpropyl-*C*H_2_), 10.4 (CH); 29.8 (CH_2_-*C*H_2_-COOH), 34.2 (*C*H_2_-CH_2_-COOH), 180.4 (*C*OOH); data fit with literature, except δ(*C*O) [68,69].

#### 3.4.3. (*E*) 3-Cyclopropylpropenoic Acid (**p3**)

A mixture of 500 mg (7,13 mmol) cyclopropanecarbaldehyde, 1.04 g (10 mmol) malonic acid, and 0.94 mL pyridine was stirred under argon for 6 h at 105 °C bath temperature, cooled to room temperature, acidified with ca. 7 mL 2 M HCl, and cooled in an ice bath. The precipitate was filtered with suction, washed with water, and dried over P_2_O_5_ *in vacuo* resulted in **p3** (270 mg, yield 34%; drying caused loss due to sublimation) [70]. ^1^H-NMR (CDCl_3_) data fit with literature values [31]; ^13^C-NMR (CDCl_3_): *δ* 9.0 (*C*H_2_), 14.6 (cyclopropane *C*H), 117.4 (C-α), 157.1 (C-β), 172.2 (COOH).

#### 3.4.4. 2-Cyclopropyl-2-Oxoacetic Acid (**p4**)

KMnO_4_ (4.42 g, 28 mmol) was solved in water (38 mL). Acetylcyclopropane (1.26 g, 15 mmol) was added under stirring, the mixture was heated to 30 °C, and 10% aq. KOH (6 mL) was added. The mixture was then gently heated with an oil bath and stirred for a further hour at 60 °C, cooled to room temperature, and filtered. The filter cake was washed with water (10 mL). The filtrate and washing solutions were combined, concentrated to about 15 mL, acidified with concentrated HCl to pH 2 and extracted with CH_2_CI_2_ (5 × 30 mL). Drying over Na_2_SO_4_ and evaporation of the solvent gave **p4** (1.33 g; 78%) [71]. ^1^H and ^13^C; NMR data fit with literature [32].

### 3.5. Cell Culture

WM9, WM164, and MUG-Mel2 melanoma cell lines were cultivated in RPMI1640 medium (Gibco, Thermo Fisher Scientific, Waltham, MA, USA). The medium was supplemented with 2 mM L-glutamine, 10% fetal bovine serum (FBS, Gibco), and 1% penicillin/streptomycin solution (Pen/Strep, Gibco). HEK-293 (human epithelial cells) were grown in Dulbecco’s Modified Eagle Medium: Nutrient Mixture F-12 (DMEM/F12, Gibco^®^), supplemented with L-glutamine (2mM), 10 % FBS, and 1 % Pen/Strep. All cells were grown in a humidified 5% CO_2_ atmosphere and at 37 °C. They were passaged by trypsinization when reaching 90% confluence using a 0.25% trypsin-EDTA solution (Gibco).

### 3.6. XTT Viability Assay

To study cell viability, the cell proliferation kit II (XTT) (Roche Diagnostics, Mannheim, Germany; cat. no. 11 465 015 001) was used and performed according to the manufacturer’s instructions. Cell suspensions with 50,000 c/mL were prepared and 100 µL seeded in each well of a 96 well plate (clear plate, flat bottom). To allow the cells to adhere, cells were incubated for 24 h before the treatment was started. Control cells were then treated with 0.5% ethanol; all other cells were treated with different concentrations (1.0 µM, 5.0 µM, and 10.0 µM) of one of the shikonin derivatives. After 72 h of incubation, 50 µL of a freshly prepared XTT solution consisting of 5 mL XTT solution plus 100 µL electron coupling reagent were added to each well and incubated for another 2 h at 37 °C. Finally, the absorbance was measured with a Hidex Sense Microplate Reader (Hidex, Turku, Finland) at 490 nm and a reference wavelength of 650 nm. **2** was always tested as a reference at 5.0 µM. The assay was performed with three replicates and two different cell passages at least.

### 3.7. ApoToxGlo^TM^ Triplex Assay

The ApoToxGlo^TM^ Triplex Assay was purchased from Promega (Fitchburg, WI, USA, cat. no. G6320) and performed according to the manufacturer’s instructions. In brief, 10,000 cells/well (WM9 or MUG-Mel2 cells) in 100 µL of medium were pipetted in 96-well plates (white plates, flat bottom), incubated for 24 h and then treated with different concentrations of **5** for 4 h, 24 h and 48 h. Staurosporine (Abcam, Cambridge, UK) at a concentration of 25.0 µM is known to induce apoptosis and served as positive control. Subsequently, the viability/cytotoxicity reagent was prepared and 20 µL added to each well. After mixing for 30 s (orbital shaking, 300–500 rpm), the plates were incubated for another 30 min at 37 °C. Fluorescence was then measured at 400_Ex_/505_Em_ (viability) and 485_Ex_/520_Em_ (cytotoxicity) using a Hidex Sense Microplate Reader. Afterwards, the Caspase-Glo^®^ 3/7 reagent was prepared and 100 µ were added to each well, followed by mixing (orbital shaking, 300–500 rpm, 30 s), and incubation at room temperature for another 30 min. Finally, luminescence was measured using a Hidex Sense Microplate Reader. The assay was performed at least two times, with three replicates each.

### 3.8. LDH Assay

To measure LDH release, the CytoTox 96^®^ Non-Radioactive Cytotoxicity Assay (Promega) were used in accordance with the manufacturers protocol. Cell suspension of 100,000 c/mL were prepared and seeded into 96 well plates (clear, flat bottom, 100 µL/well). To allow the cells to adhere, cells were incubated for 24 h before the treatment was started. Afterwards, cells were treated with 1 µM, 5 µM, 10 µM, or 20 µM of **5** for 24 h, 48 h, or 72 h. To measure maximum LDH release, cells were lysed using the lyse reagent included in the assay kit. To quantify LDH release, the plates were centrifuged and 50 µL of each well were transferred into another 96 well plate. After addition of 50 µL CytoTox96 Reagent for 30 min, 50 µL of the stop solution were added and absorption were measured at 490 nm using a Hidex Sense Microplate Reader. Finally, using the following formula, the amount of LDH release was calculated: 100 × OD_490_ test well/OD_490_ maximum LDH release.

### 3.9. Cell Cycle Analysis

For cell cycle analysis, 2 mL of a 150,000 c/mL cell suspension were seeded into 6 well plates and incubated for 24 h. Afterwards, the cells were treated with 10 µM or 20 µM of **5** for 24 h and 48 h. The cells were collected including supernatants and by using trypsinization, centrifugation, and washing with RPMI cell culture medium. Finally, cells were resuspended in 500 µL PBS and fixed with 5 mL ice cold ethanol for 10 min. For FACS analyses, cells were centrifuged and resuspended with 200 µL PI lysis buffer. After incubation for 20 min in the dark, cells were analyzed using a LSRII™flow cytometer (BD Biosciences, Franklin Lakes, NJ, USA) and ModFit software.

## 4. Conclusions

In previous studies, shikonin and derivatives have been demonstrated to be potent cytotoxic substances. In this study, we prepared 31 shikonin derivatives by synthesis. Most of them are novel derivatives, which have not yet been reported. One goal was to further optimize our previous hit **3**. Another goal was to synthesize a broad spectrum of structural features to gain more insight into the structure-activity relationship. All derivatives were screened for their cytotoxicity in several melanoma cell lines. The results indicate that there is no strict structure-activity relationship and the different cell lines exhibited distinct sensitivities towards the derivatives. The most potent derivative was **5**, which is a cyclopropyloxoacetate derivative of shikonin and, thus, structurally related to our previous hit **3**. Compared to **3**, **5** was more cytotoxic. Subsequent pharmacological investigations revealed that **5** leads to caspase 3/7 activation, no significant LDH release, and to a G2/M phase cell cycle arrest at higher concentrations. Nevertheless, it was also cytotoxic towards non-tumorigenic cells, which needs to be evaluated in more detail in future studies. In summary, our results indicate that shikonin derivatives might be potential drug leads for the development of novel melanoma treatment options.

## Figures and Tables

**Table 1:**
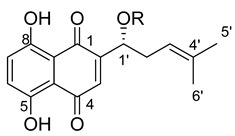
Structures of shikonin (**1**) and derivatives thereof.

Compound	R	Compound	R
**1**	H	**2**	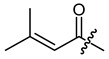
**3**	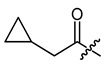	**4**	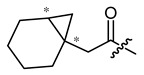
**5**	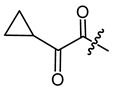	**6**	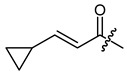
**7**	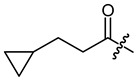	**8**	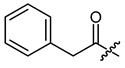
**9**	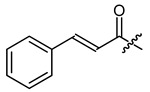	**10**	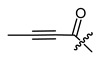
**11**	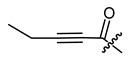	**12**	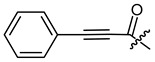
**13**	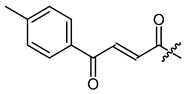	**14**	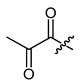
**15**	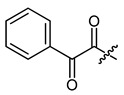	**16**	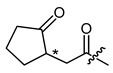
**17**	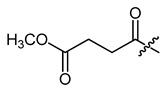	**18**	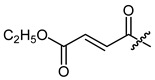
**19**	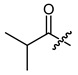	**20**	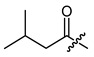
**21**	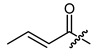	**22**	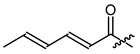
**23**	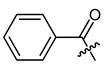	**24**	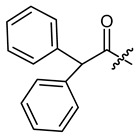
**25**	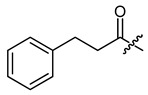	**26**	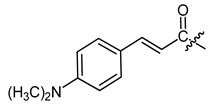
**27**	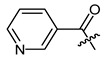	**28**	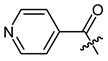
**29**	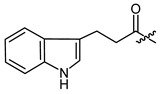	**30**	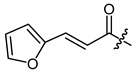
**31**	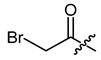	**32**	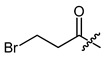
**33**	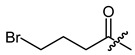	**34**	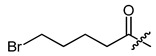

**Table 2:** IC_50_ values (µM) of **2**, **3**, and **5** after 72 h of treatment and as determined using SigmaPlot 14.0 and the four-parameter logistic curve (*n* = 6, mean ± sem). n.d. = not determined.

Cell Line	IC_50_ Values of 5 (µM)	IC_50_ Values of 3 [17] (µM)	IC_50_ Values of 2 [14] (µM)
WM9	1.5 ± 0.1	3.2 ± 0.8	2.7 ± 0.3
WM164	4.5 ± 0.5	4.9 ± 1.7	8.3 ± 0.3
MUG-Mel2	2.4 ± 0.2	3.2 ± 0.3	7.2 ± 0.5
HEK293	3.4 ± 0.2	5.4 ± 0.7	n.d.

**Table 3:** Calculated p-values when comparing the amount of control and treated cells in the G2/M phase (student’s *t*-test, *n* = 6). The number of cells in the S-phase was not statistically significantly changed.

Cell Line	10 µM (24 h)	20 µM (24 h)	10 µM (48 h)	20 µM (48 h)
WM9	0.0311	0.0014	0.1467	0.0515
WM164	0.3313	0.0153	0.0553	0.0065

## Data Availability

Not applicable.

## Supplementary Materials

The following are available online at https://www.mdpi.com/1422-0067/22/5/2774/s1.
