# Supplementary material for: Synthesis and Pharmacological In Vitro Investigations of Novel Shikonin Derivatives with a Special Focus on Cyclopropane Bearing Derivatives
Source: Int J Mol Sci. 2021 Mar 9;22(5):2774. doi: 10.3390/ijms22052774 (PMC7967198; doi:10.3390/ijms22052774)
Supplement: Supplementary file 1 [file ijms-22-02774-s001.pdf]

International Journal of
Molecular Sciences

MDPI

Supplemental Material to:

Synthesis and pharmacological in-vitro investigations of novel shikonin derivatives with a special focus on cyclopropane bearing derivatives

Nadine Kretschmer [1], Antje Hufner [2], Christin Durchschein [1], Katrin Popodi [1], Beate Rinner [3], Birgit Lohberger [4], Rudolf Bauer [1*]

[1] Institute of Pharmaceutical Sciences, Department of Pharmacognosy, University of Graz, Beethovenstr. 8, 8010 Graz, Austria; nadine.kretschmer@uni-graz.at (N.K.); christin.durchschein@hotmail.com (C.D.); katrin.popodi@gmx.at (K.P.)
[2] Institute of Pharmaceutical Sciences, Department of Pharmaceutical Chemistry, University of Graz, Universitaetsplatz 1, 8010 Graz, Austria; antje.huefner@uni-graz.at (A.H.)
[3] Division of Biomedical Research, Medical University of Graz, Roseggerweg 48, 8036 Graz, Austria; beate.rinner@medunigraz.at (B.R.)
[4] Department of Orthopedics and Trauma, Medical University Graz, Auenbruggerplatz 5, 8036 Graz, Austria; birgit.lohberger@medunigraz.at (B.L.)
* Correspondence: rudolf.bauer@uni-graz.at (R.B.)

Content of supporting information file:

1. Results of XTT screening. 3

2. ^{1}H-NMR spectra ($CDCl_3$, 400 MHz) of all shikonin derivatives synthesized for this study and ^{13}C-NMR spectra (100 MHz) or HMBC spectra of derivatives with yet unknown ^{13}C-NMR spectra 5

3. ^{1}H NMR spectra of precursor acids.......... 34

1. Results of XTT screening.

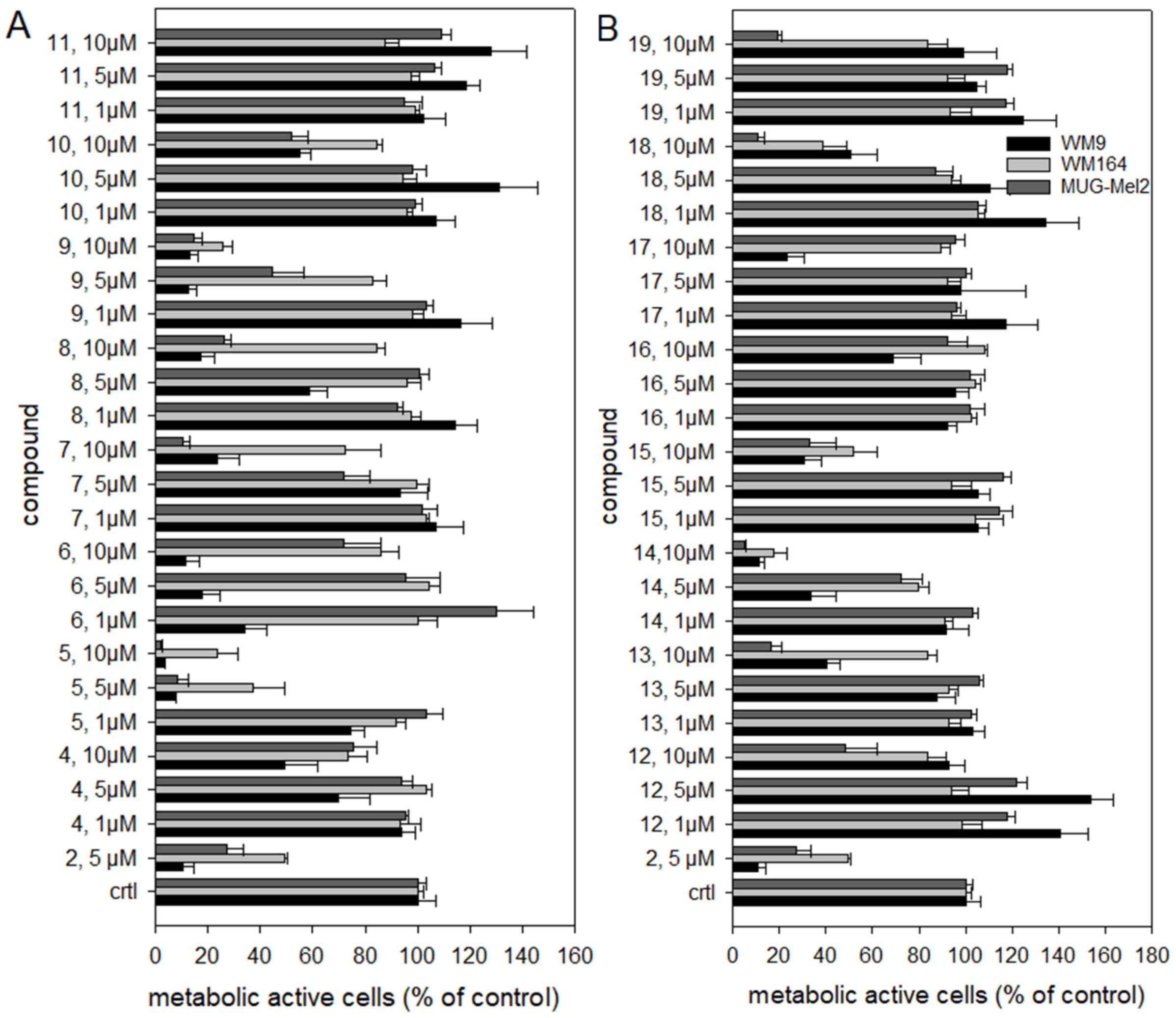

Figure S1. Results of compounds **4-11** (A) and to **12-19** (B) compared to 5.0 µM of **2**. Vinblastine was used as positive control. At a concentration of 0.01 nM, it reduced the cell viability compared to control cells to: WM9 cells: 23.8 ± 1.5 %, WM164 cells: 59.4 ± 4.4 %, and MUG-Mel2 cells: 65.0 ± 6.9 % (n = 6, mean ± sem, 72h of treatment).

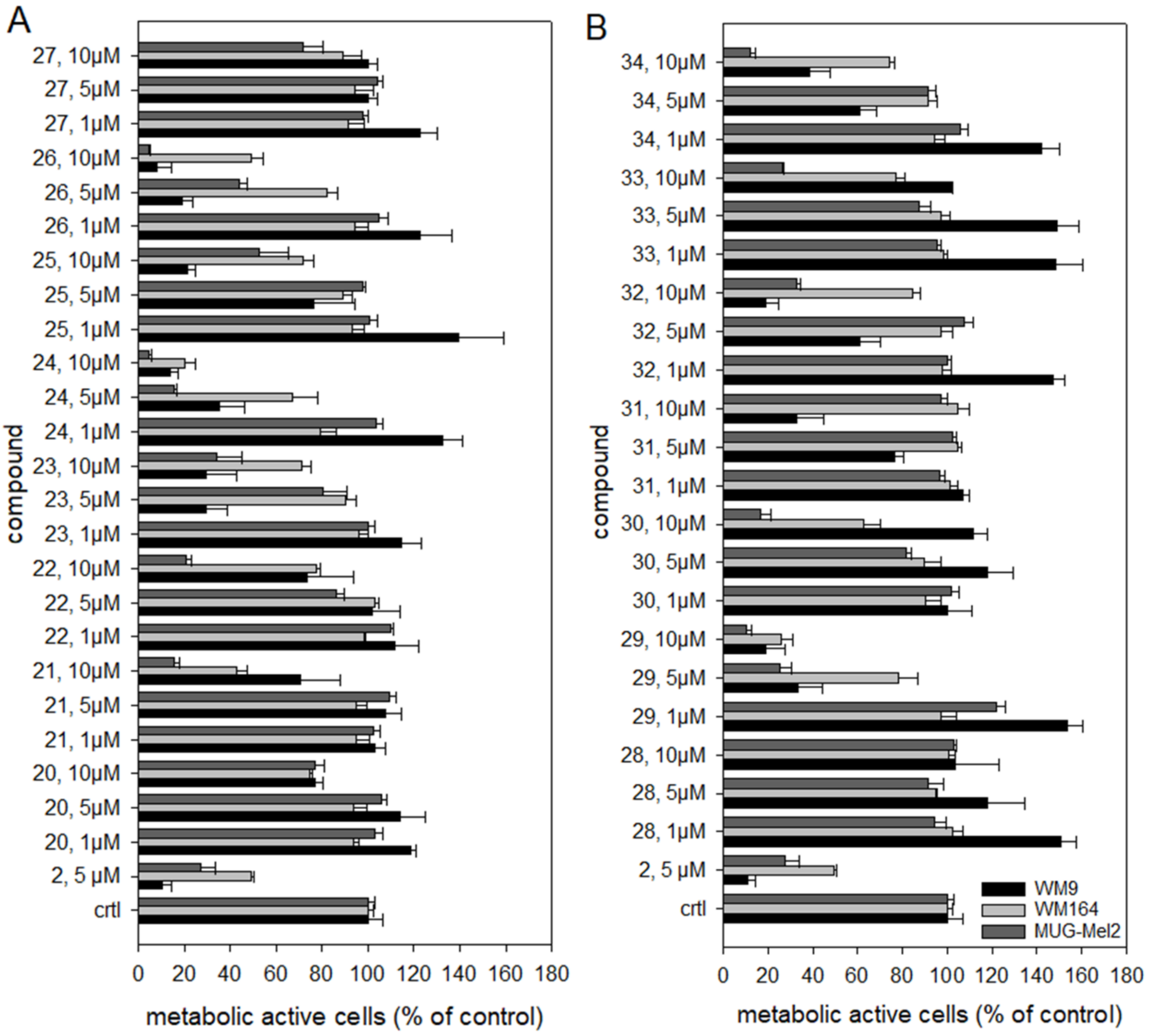

Figure S2. Results of compounds **20-27** (A) and **28**-**34** (B) compared to 5.0 µM of **2**. Vinblastine was used as positive control. At a concentration of 0.01 nM, it reduced the cell viability compared to control cells to: WM9 cells: 23.8 ± 1.5 %, WM164 cells: 59.4 ± 4.4 %, and MUG-Mel2 cells: 65.0 ± 6.9 % (n = 6, mean ± sem, 72h of treatment).

2. ^{1}H-NMR spectra (CDCl$_3$, 400 MHz) of all shikonin derivatives synthesized for this study and ^{13}C-NMR spectra (100 MHz) or HMBC spectra of derivatives with yet unknown ^{13}C-NMR spectra

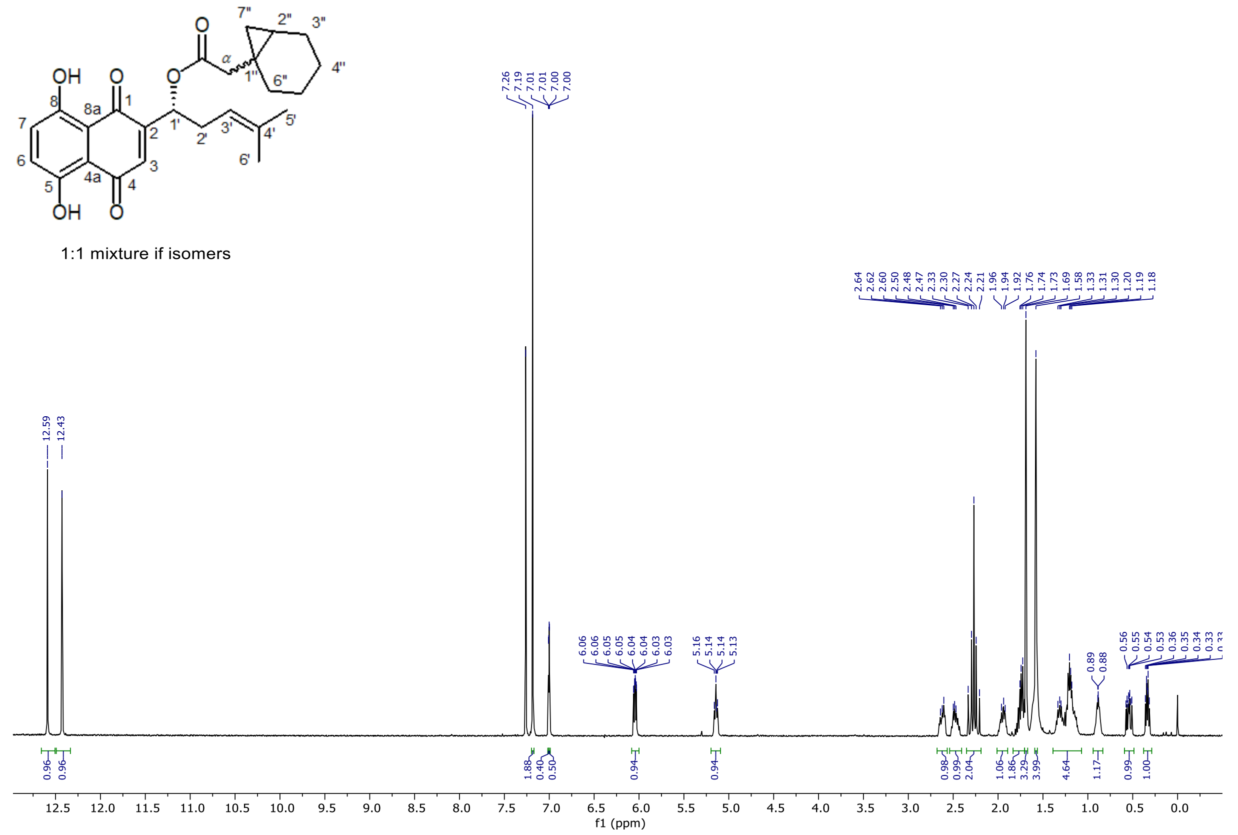

^{1}H-NMR spectrum (CDCl$_3$, 400 MHz) of **4**

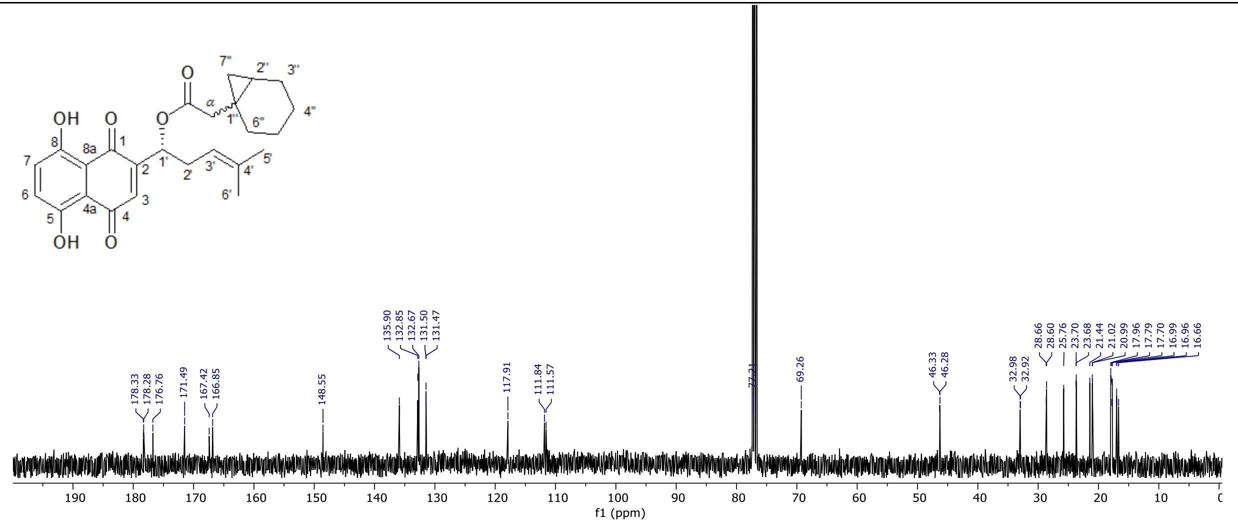

^{13}C-NMR spectrum (CDCl$_3$, 100 MHz) of **4**

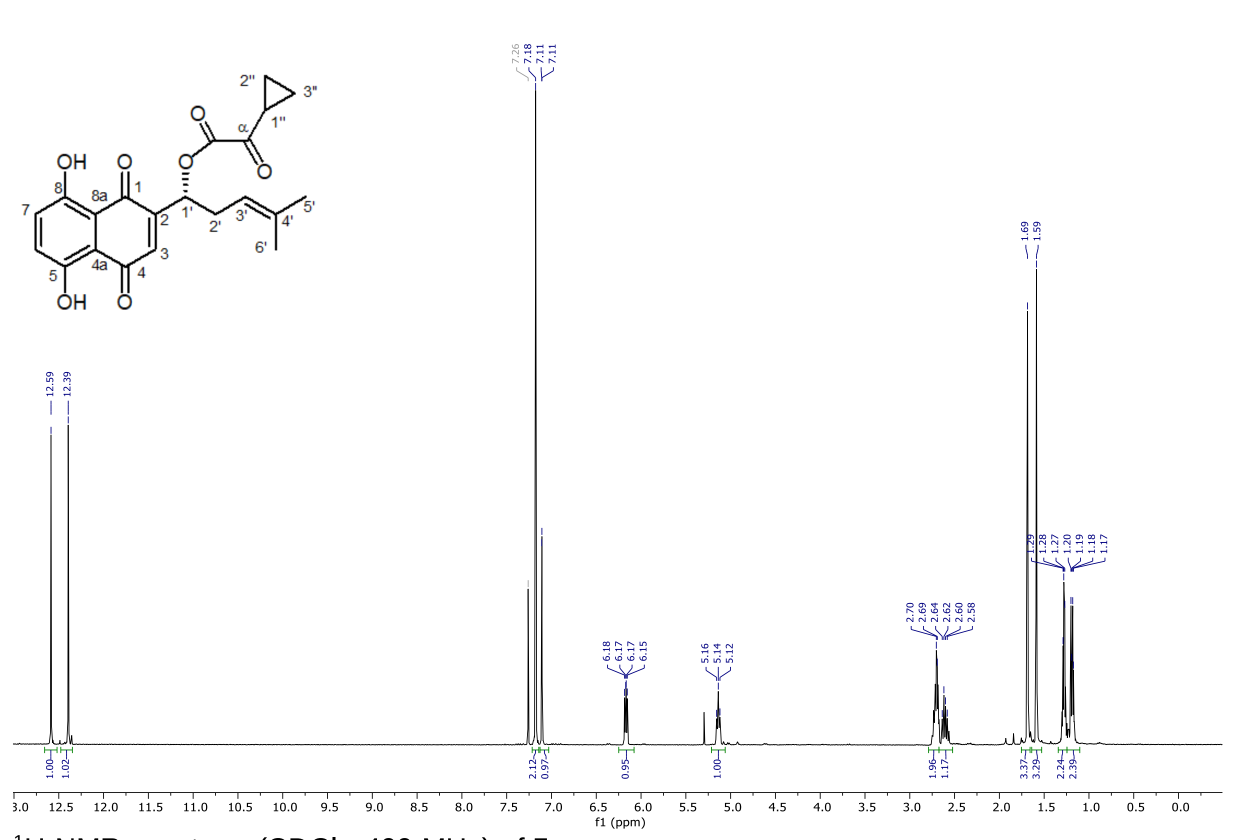

^{1}H-NMR spectrum ($CDCl_3$, 400 MHz) of **5**

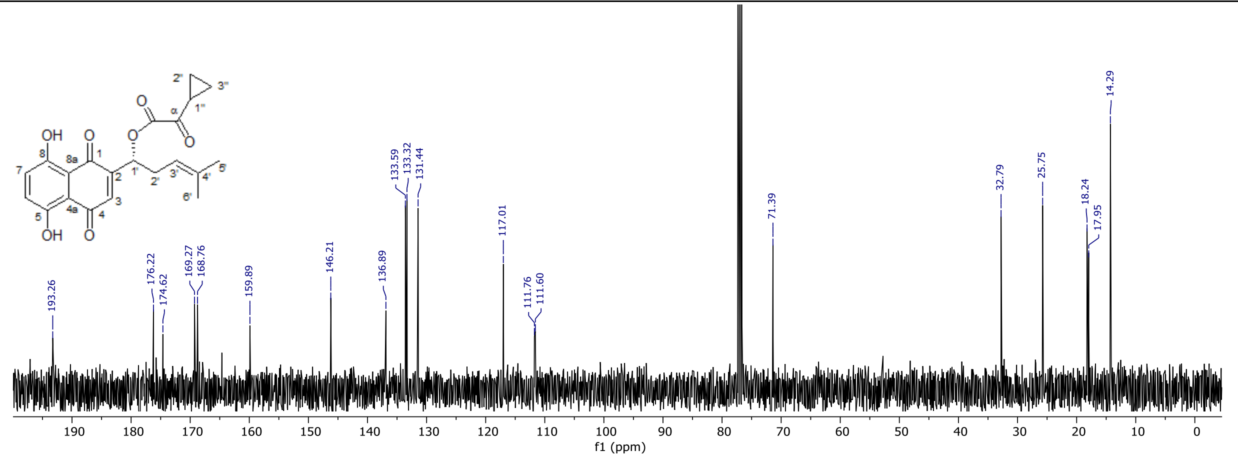

^{13}C-NMR spectrum ($CDCl_3$, 100 MHz) of **5**

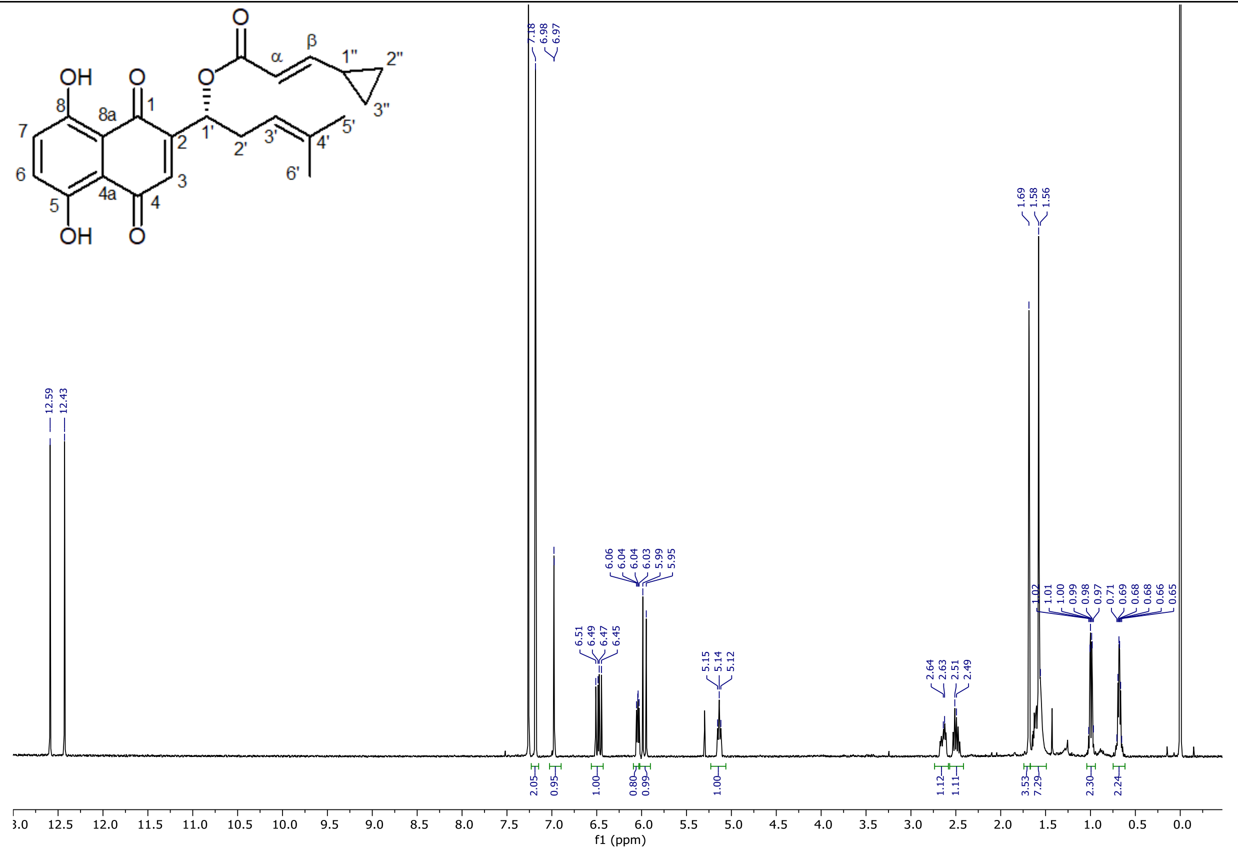

^{1}H-NMR spectrum ($CDCl_3$, 400 MHz) of **6**

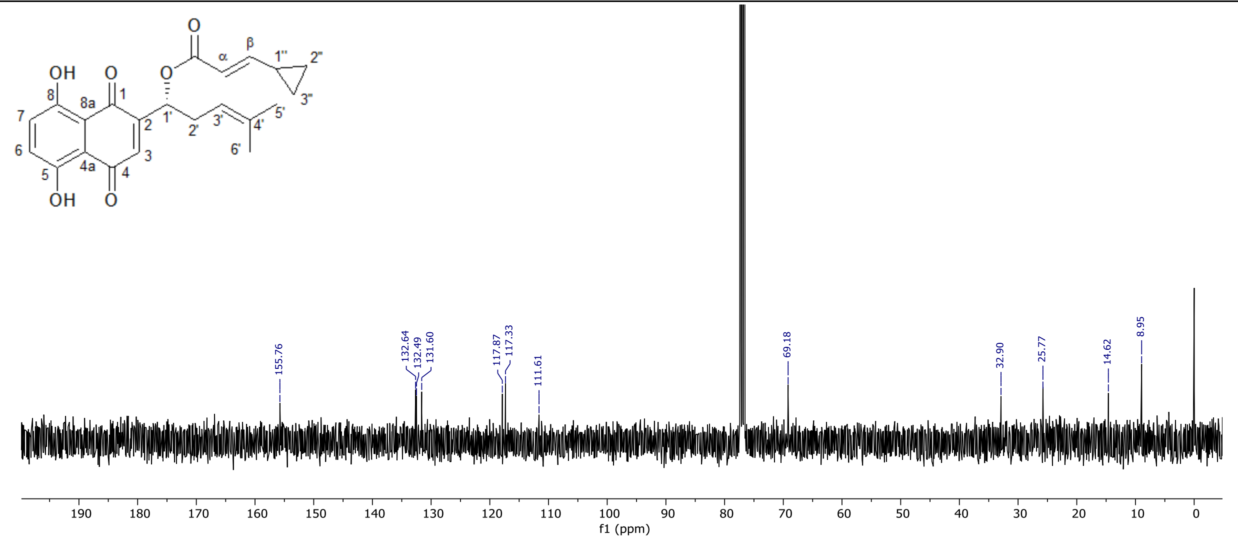

^{13}C-NMR spectrum ($CDCl_3$, 100 MHz) of **6**

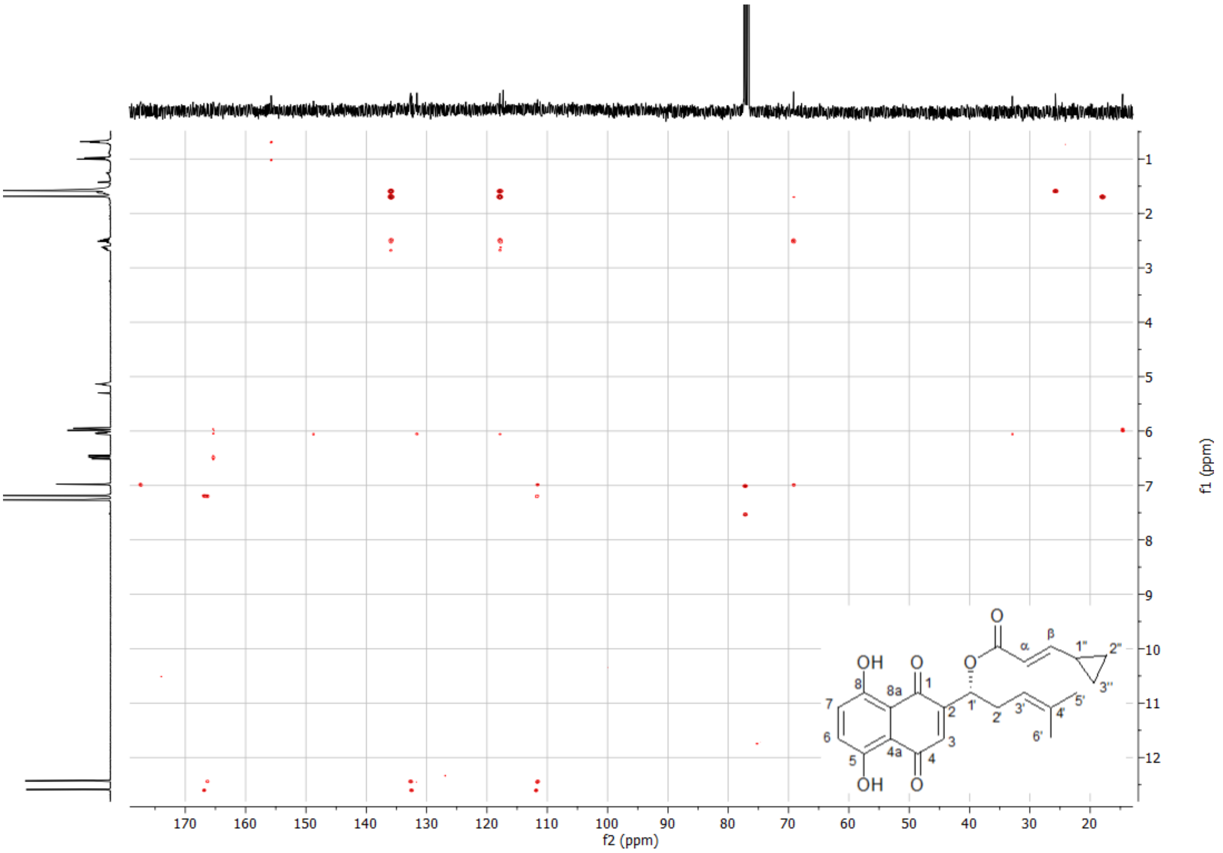

HMBC spectrum ($CDCl_3$, 400 MHz) of **6**

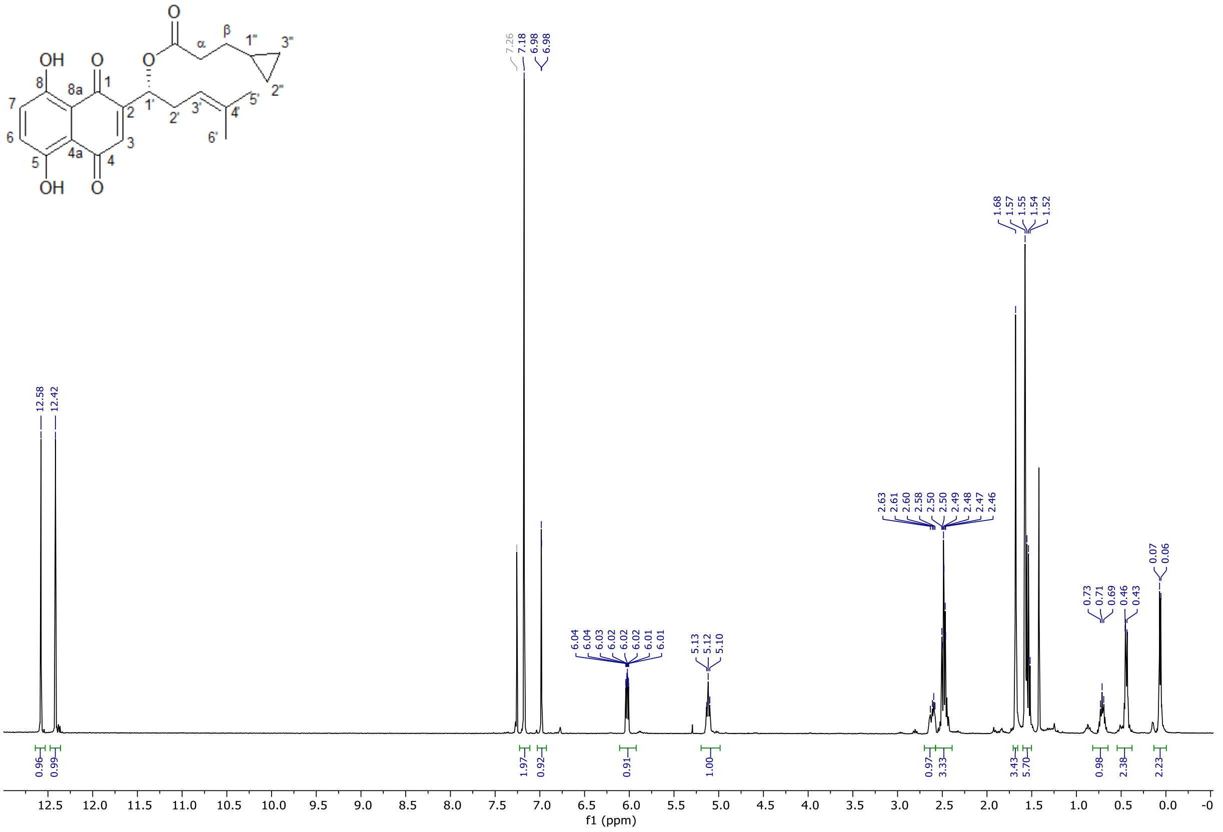

^{1}H-NMR spectrum ($CDCl_3$, 400 MHz) of **7**

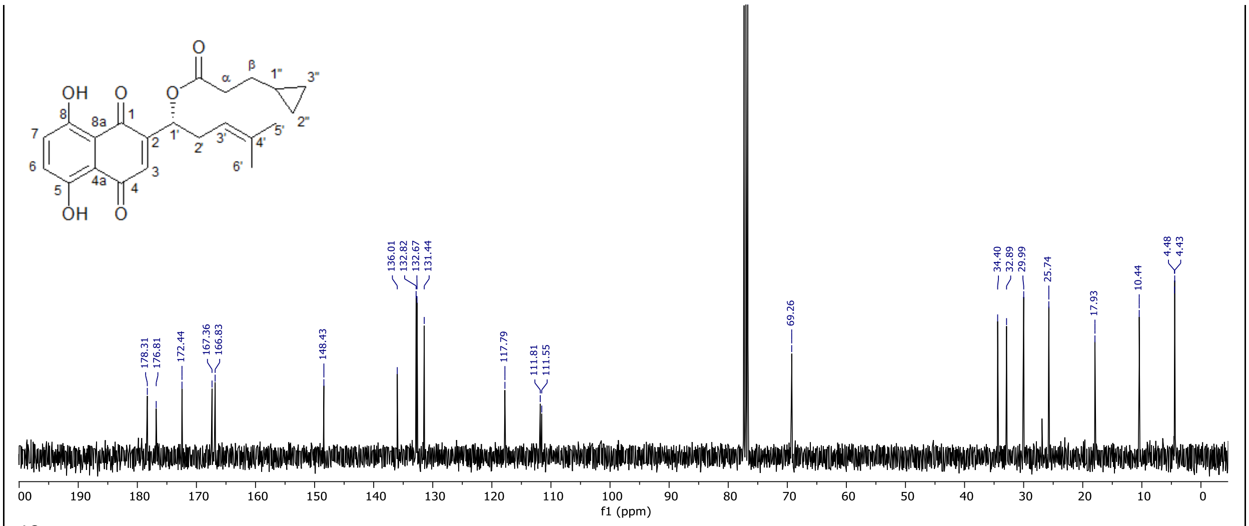

^{13}C-NMR spectrum (CDCl$_3$, 100 MHz) of **7**

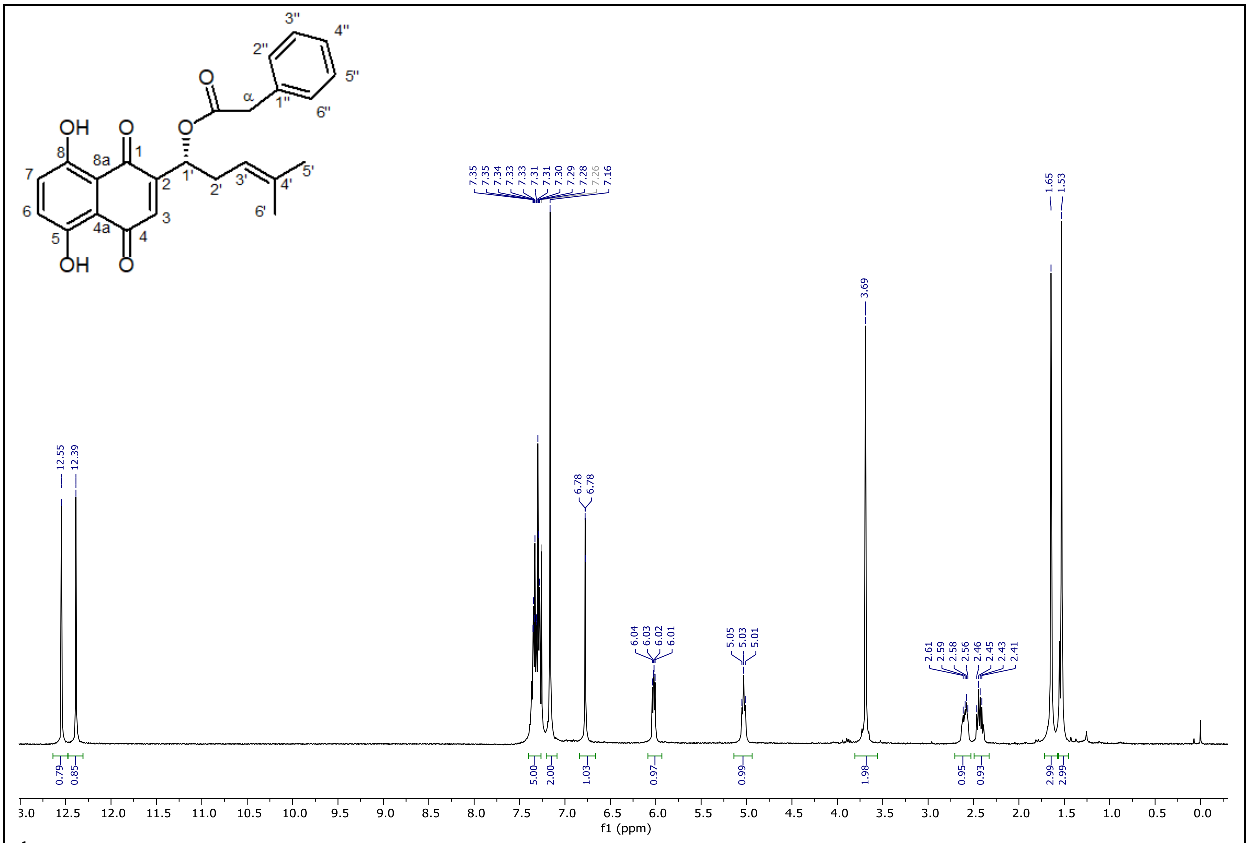

^{1}H-NMR spectrum (CDCl$_3$, 400 MHz) of **8**

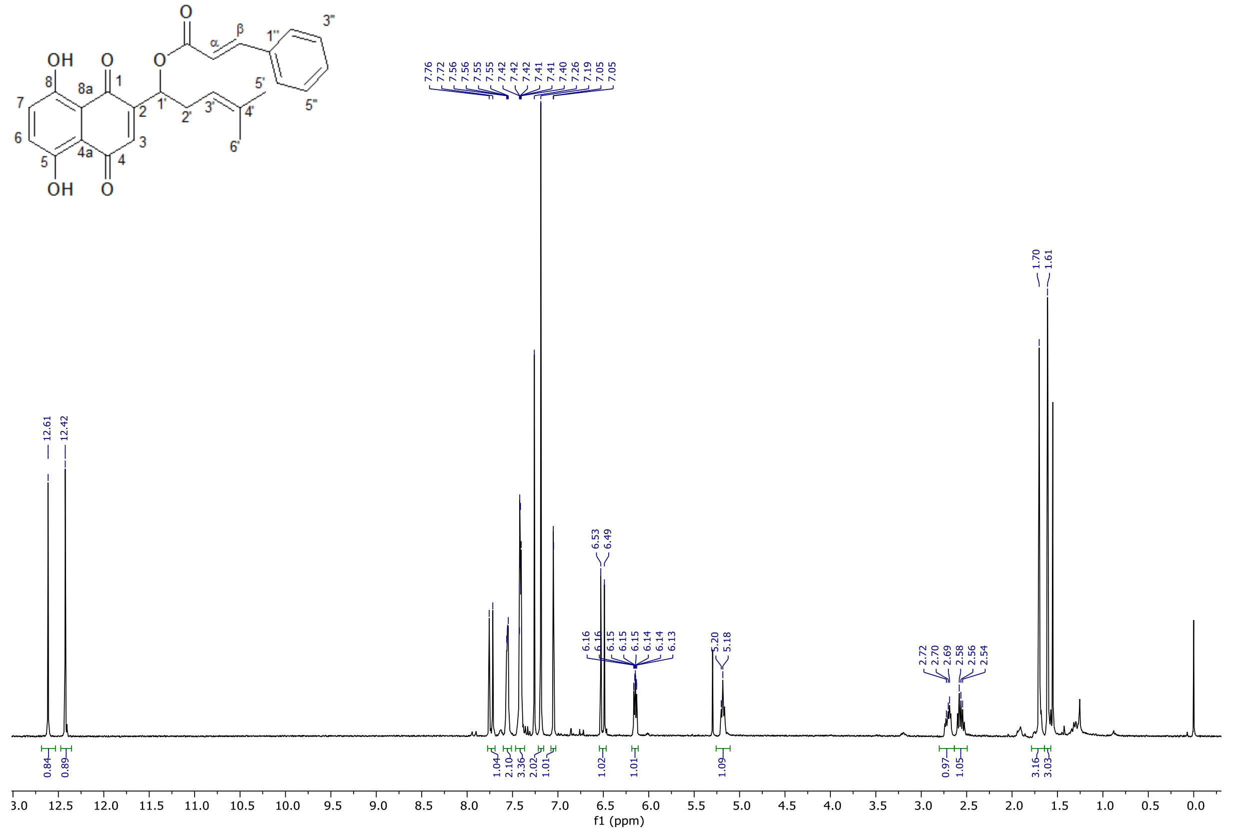

^{1}H-NMR spectrum (CDCl$_3$, 400 MHz) of **9**

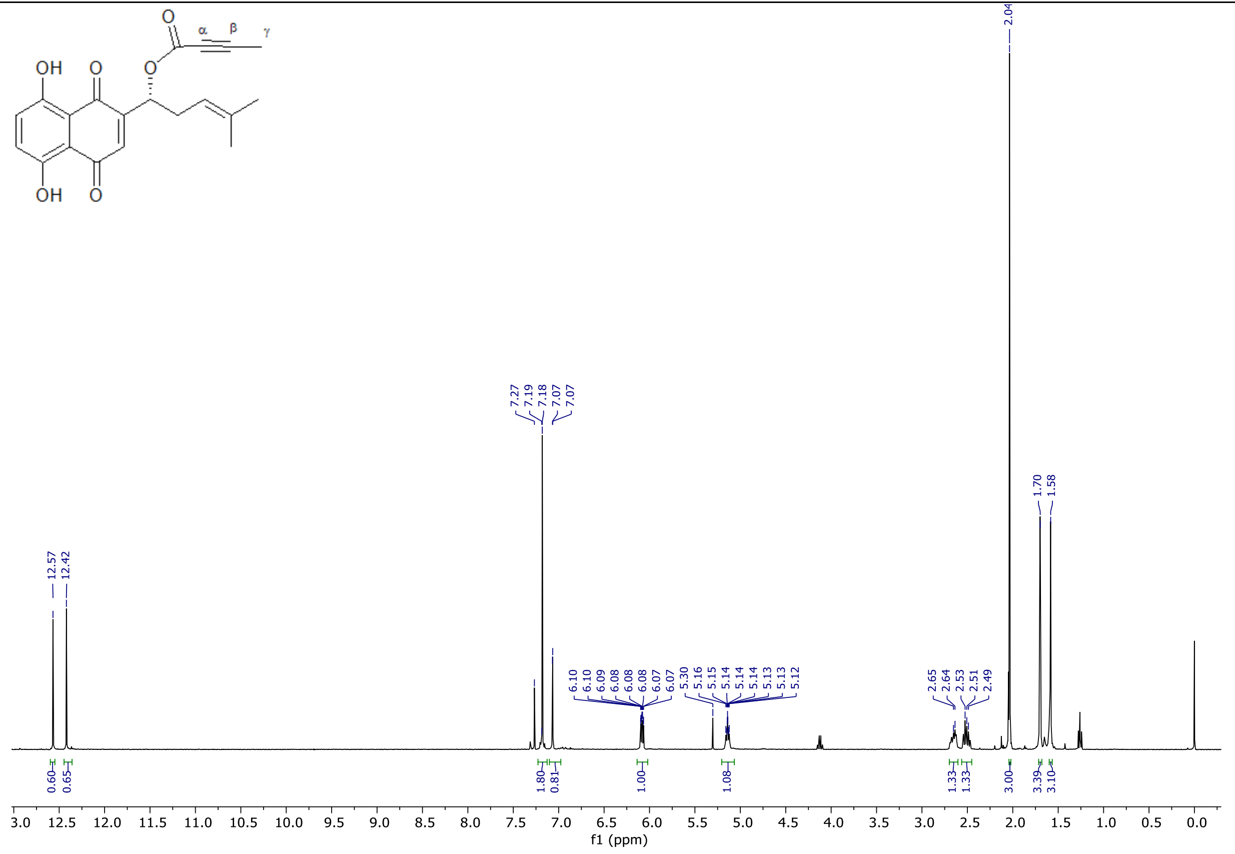

^{1}H-NMR spectrum (CDCl$_3$, 400 MHz) of **10**

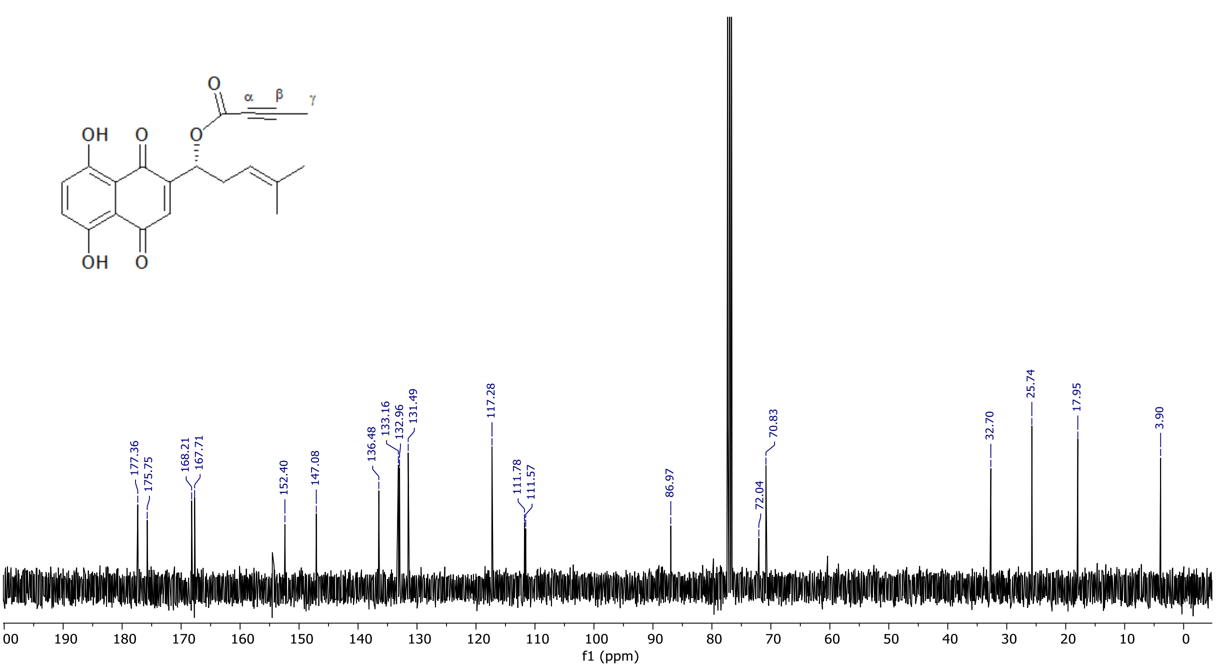

^{13}C-NMR spectrum ($CDCl_3$, 100 MHz) of **10**

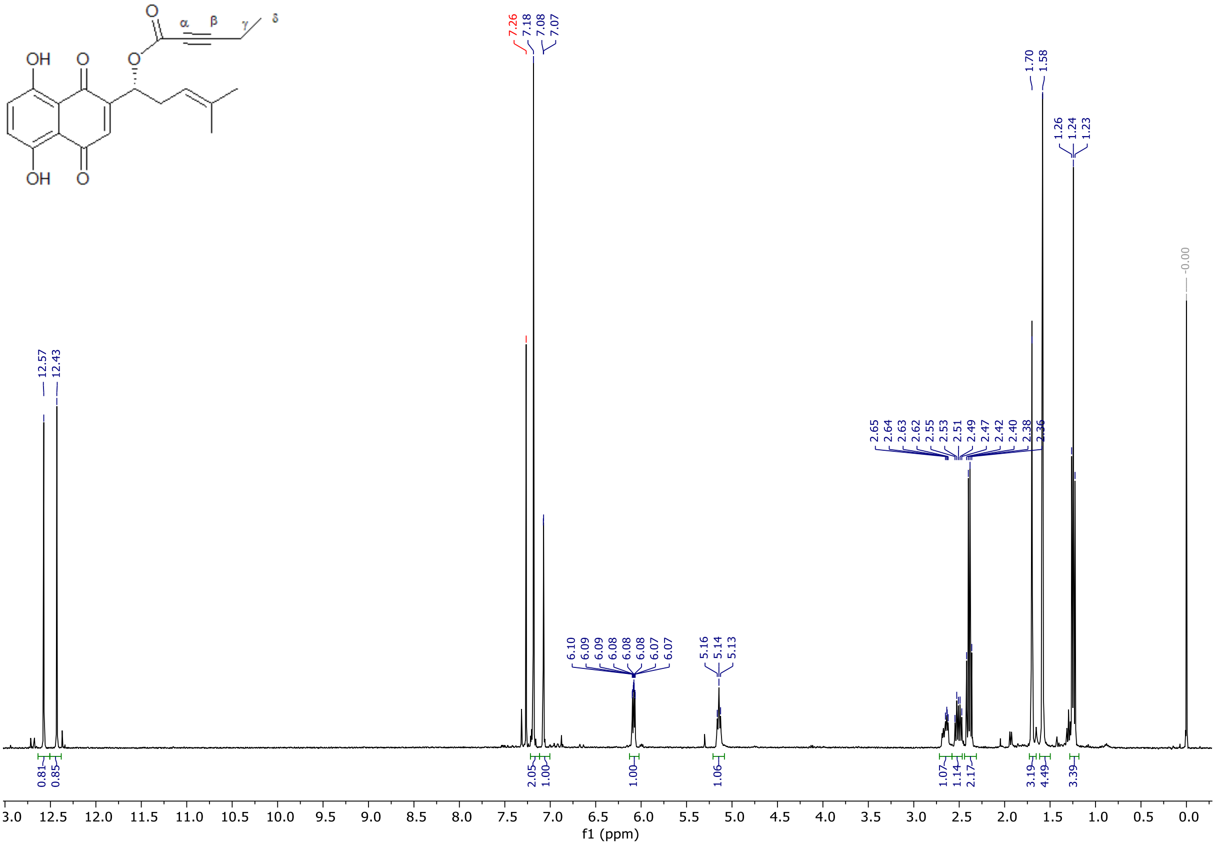

^{1}H-NMR spectrum ($CDCl_3$, 400 MHz) of **11**

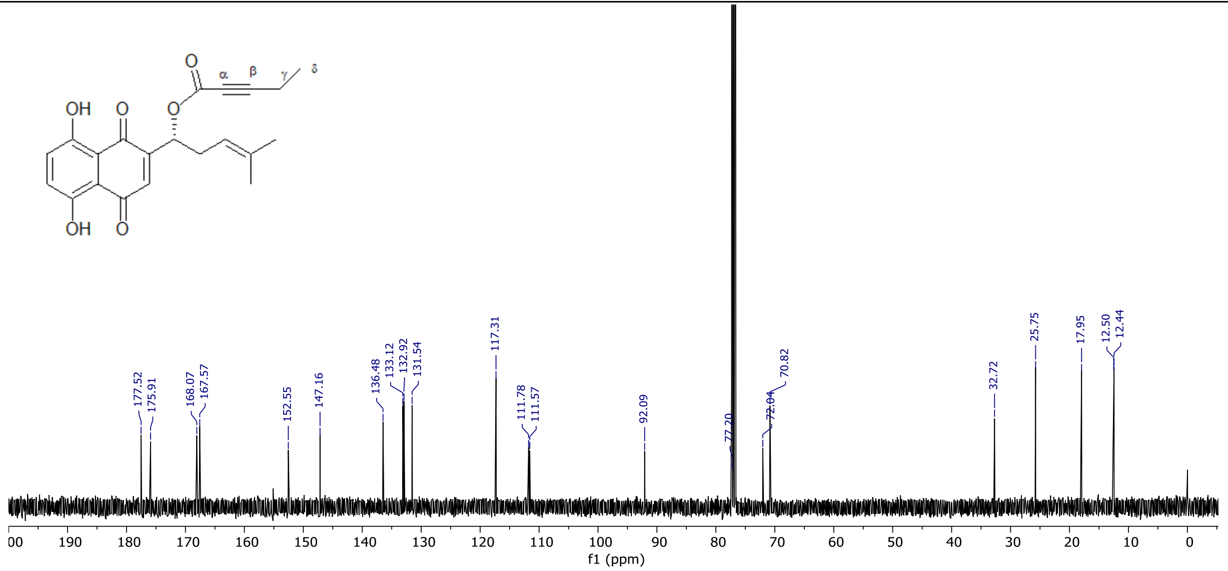

^{13}C-NMR spectrum ($CDCl_3$, 100 MHz) of **11**

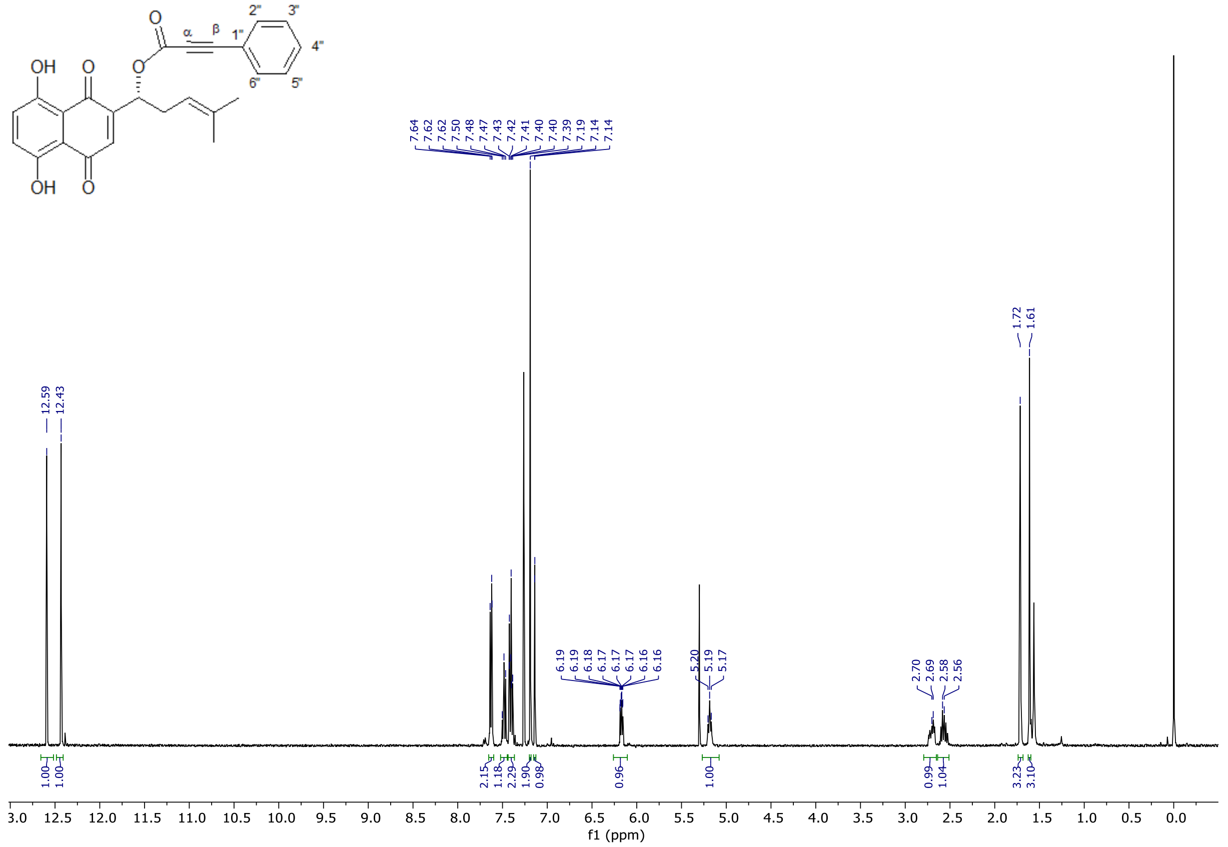

^{1}H-NMR spectrum ($CDCl_3$, 400 MHz) of **12**

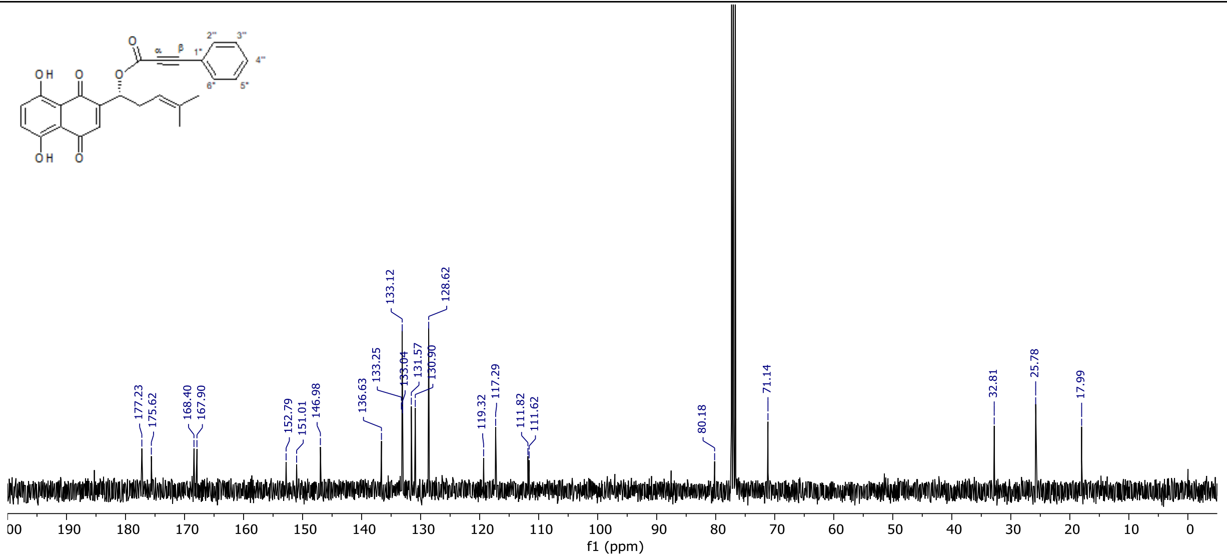

^{13}C-NMR spectrum ($CDCl_3$) of **12**

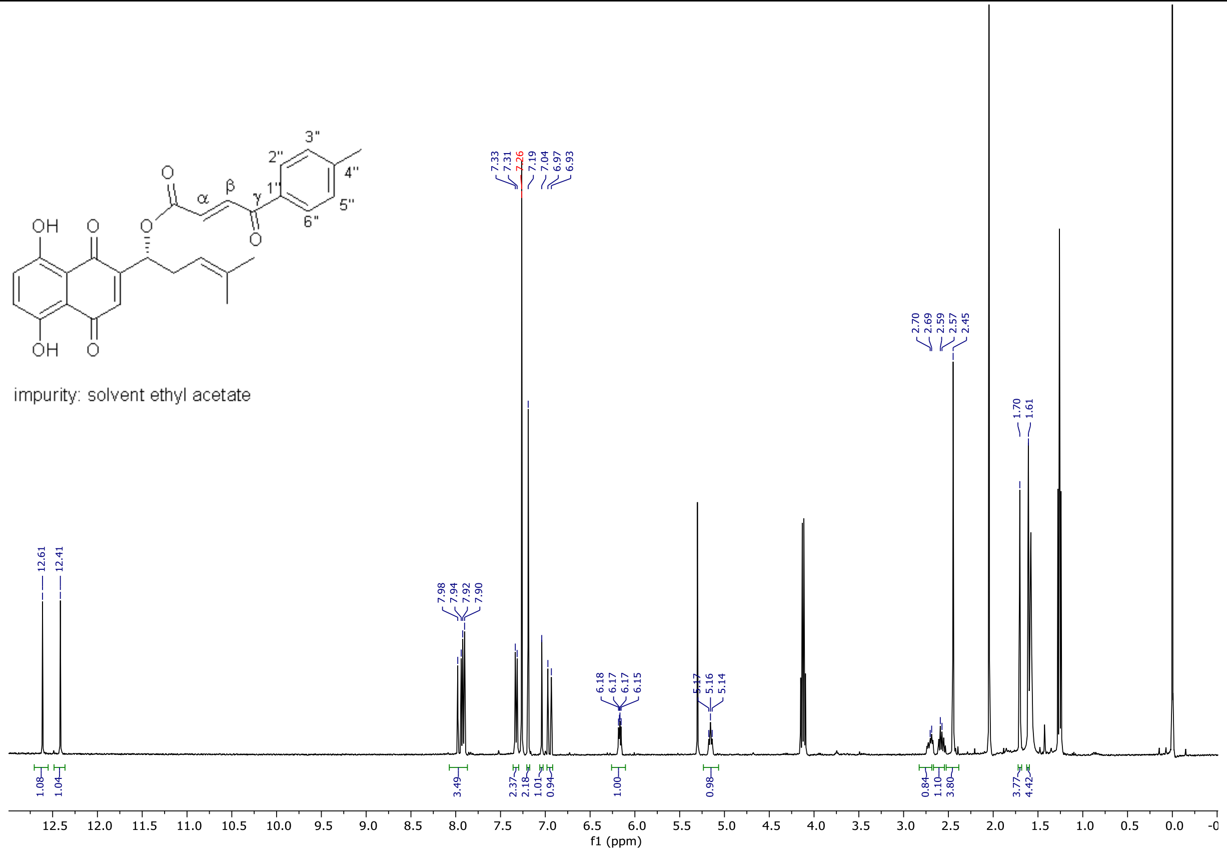

^{1}H-NMR spectrum ($CDCl_3$, 400 MHz) of **13**

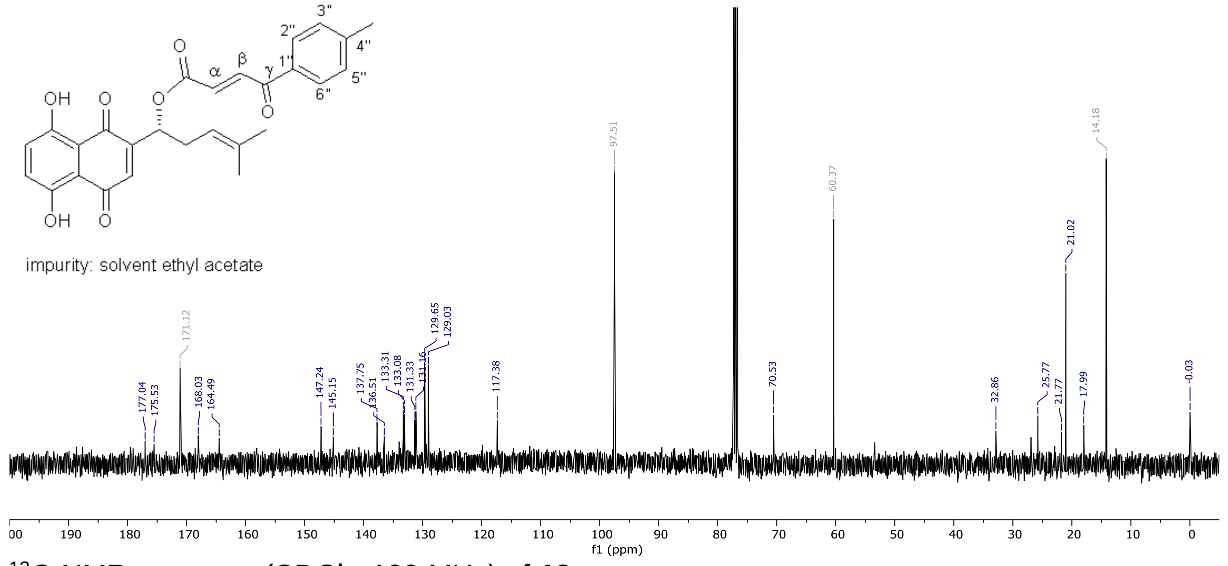

^{13}C-NMR spectrum ($CDCl_3$, 100 MHz) of **13**

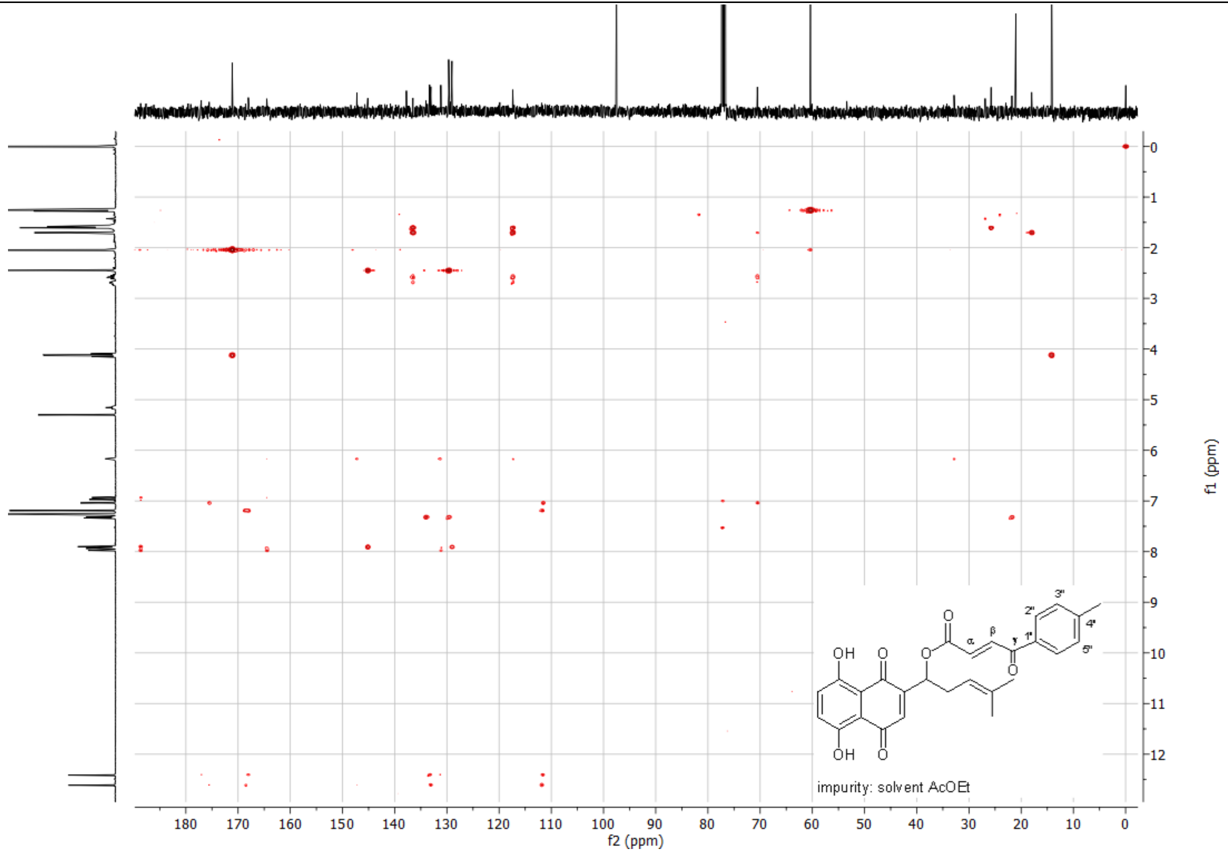

HMBC spectrum ($CDCl_3$, 100 MHz) of **13**

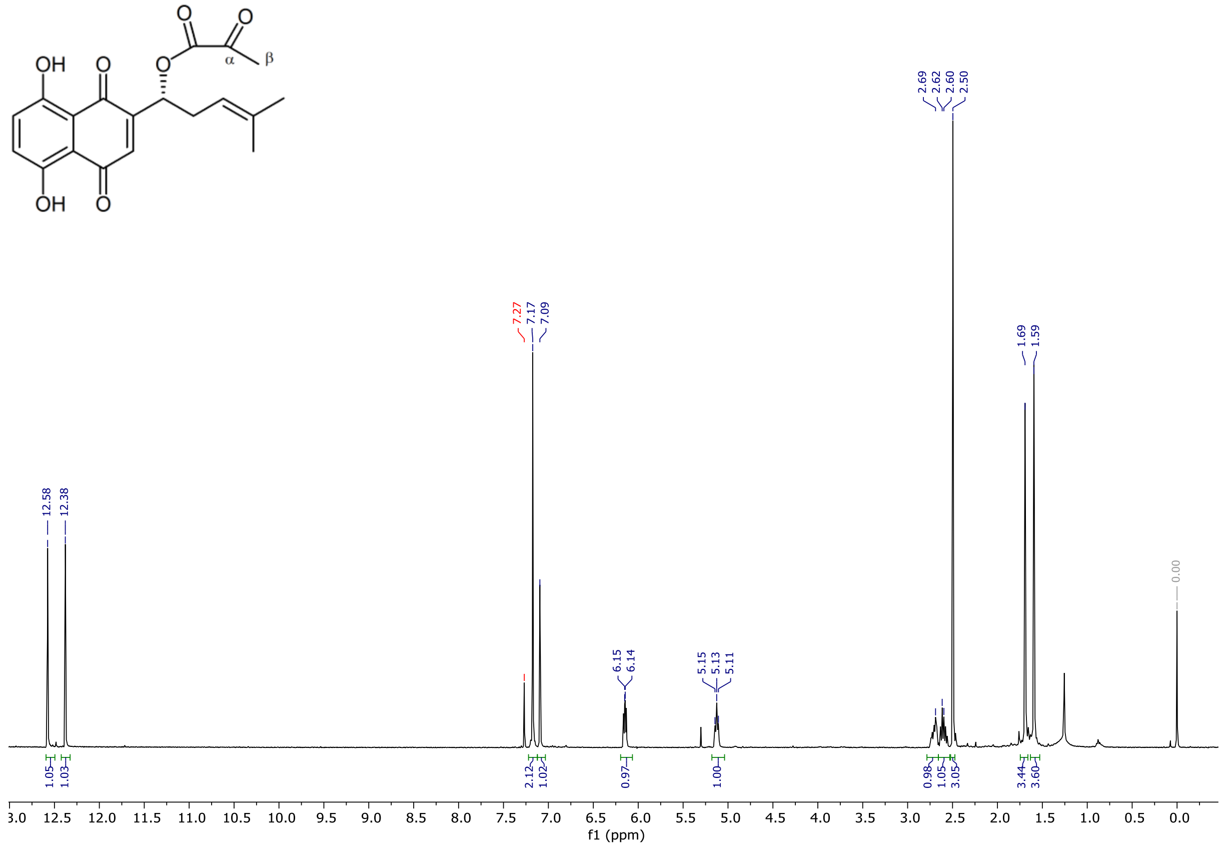

^{1}H-NMR spectrum ($CDCl_3$, 400 MHz) of **14**

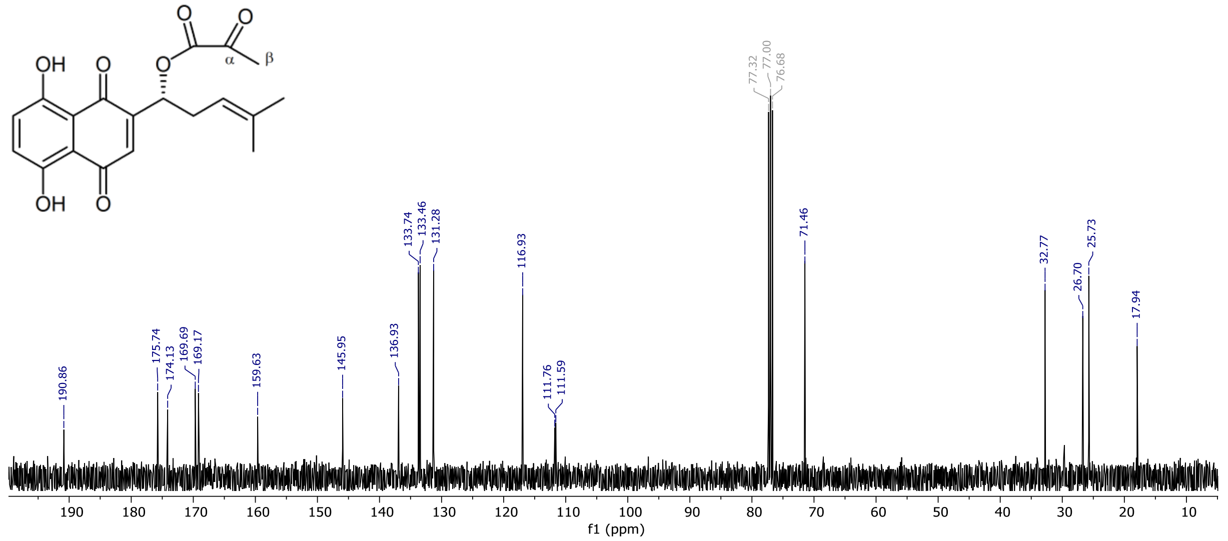

^{13}C-NMR spectrum ($CDCl_3$, 100 MHz) of **14**

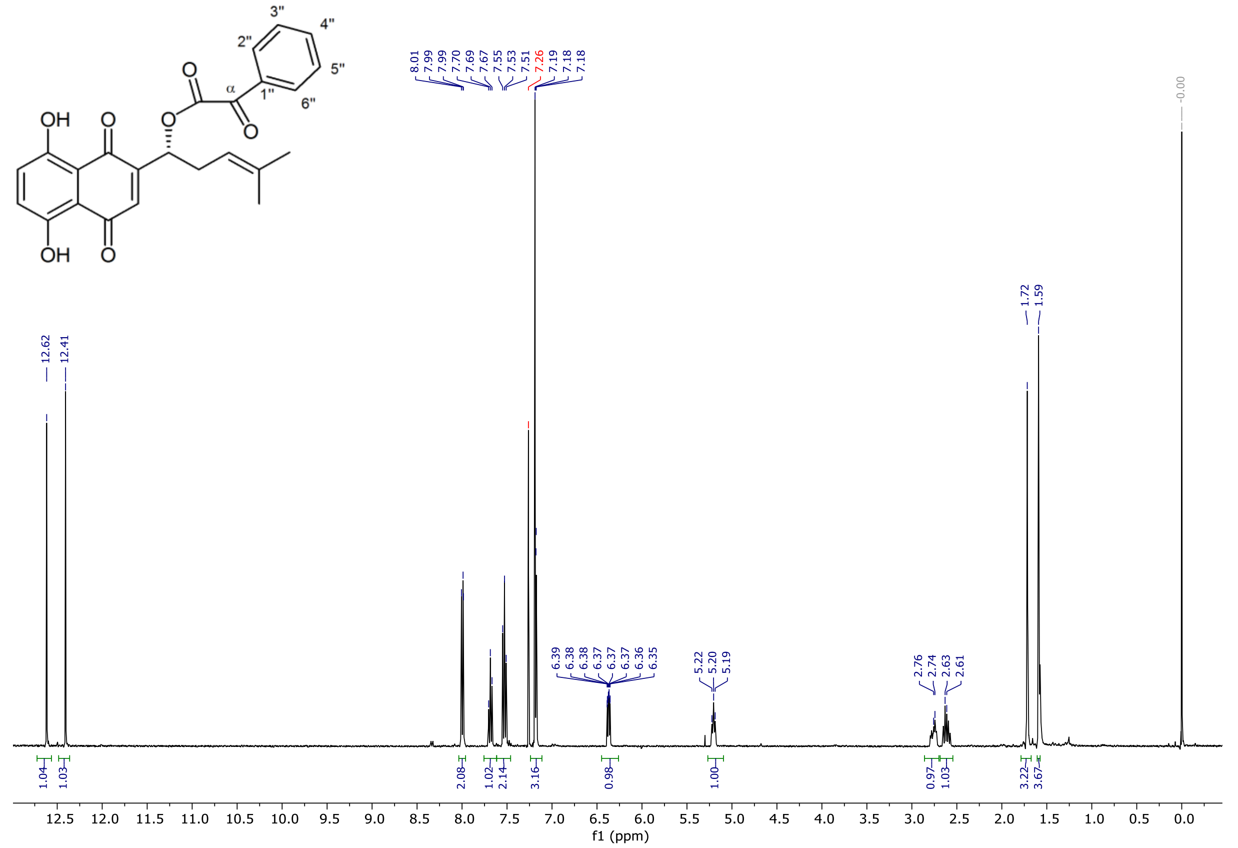

^{1}H-NMR spectrum ($CDCl_3$, 400 MHz) of **15**

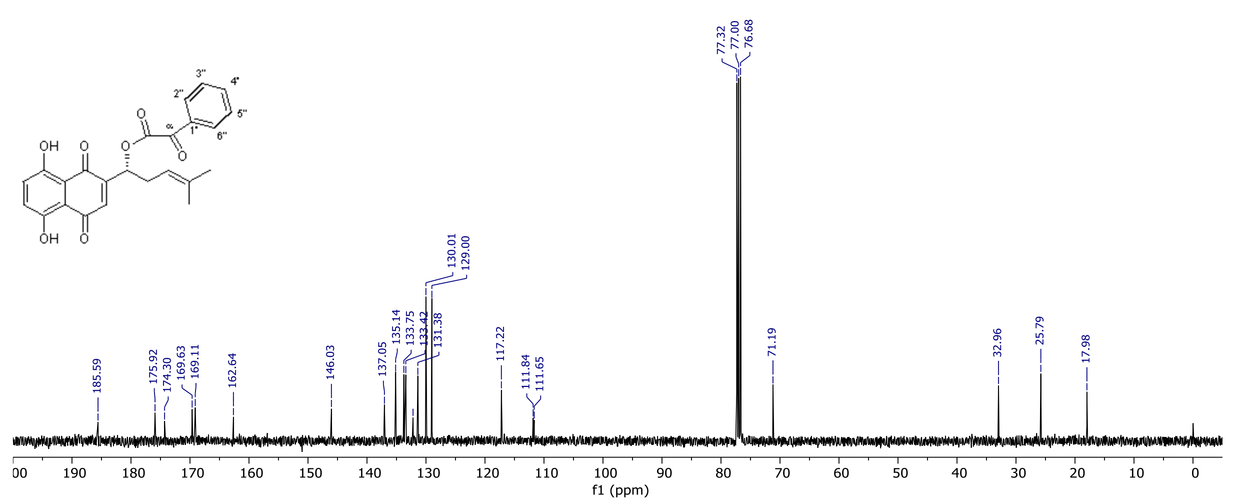

^{13}C-NMR spectrum ($CDCl_3$, 100 MHz) of **15**

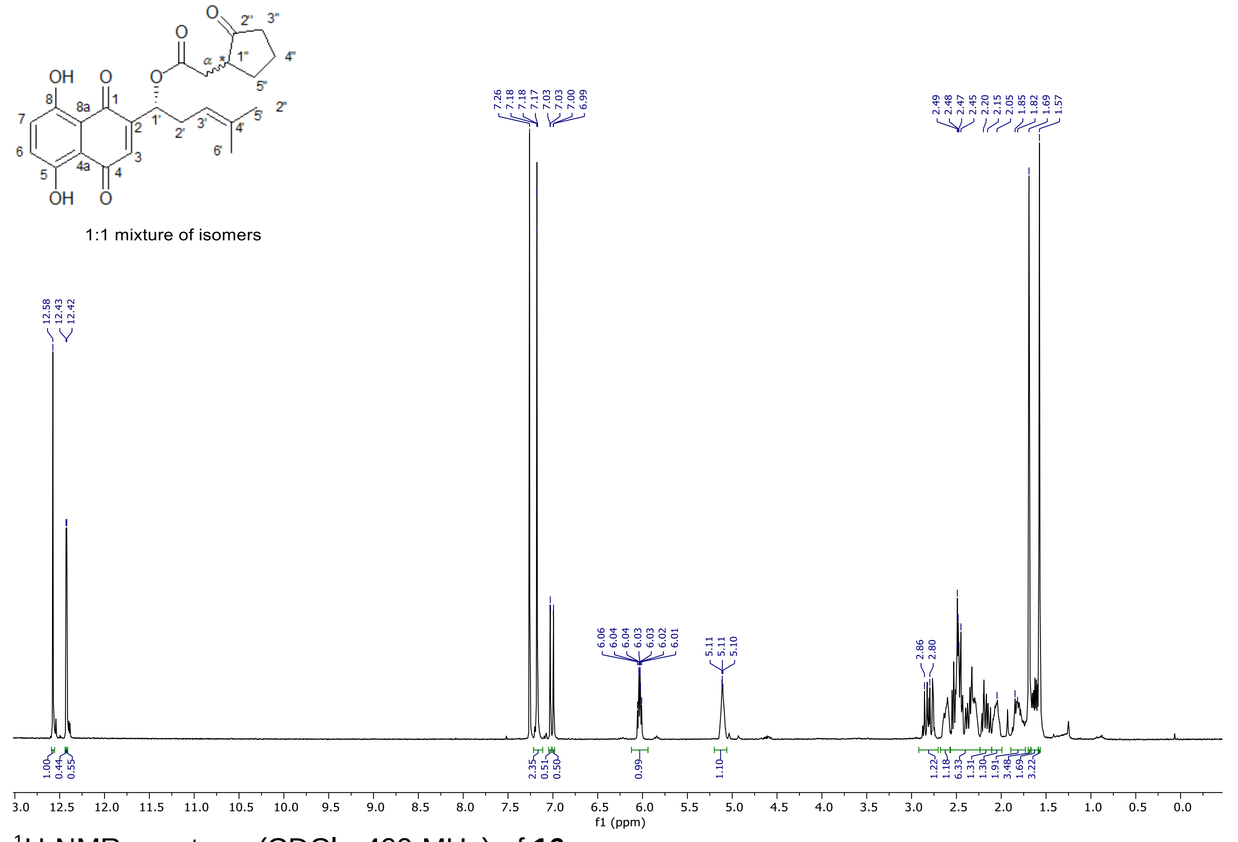

^{1}H-NMR spectrum ($CDCl_3$, 400 MHz) of **16**

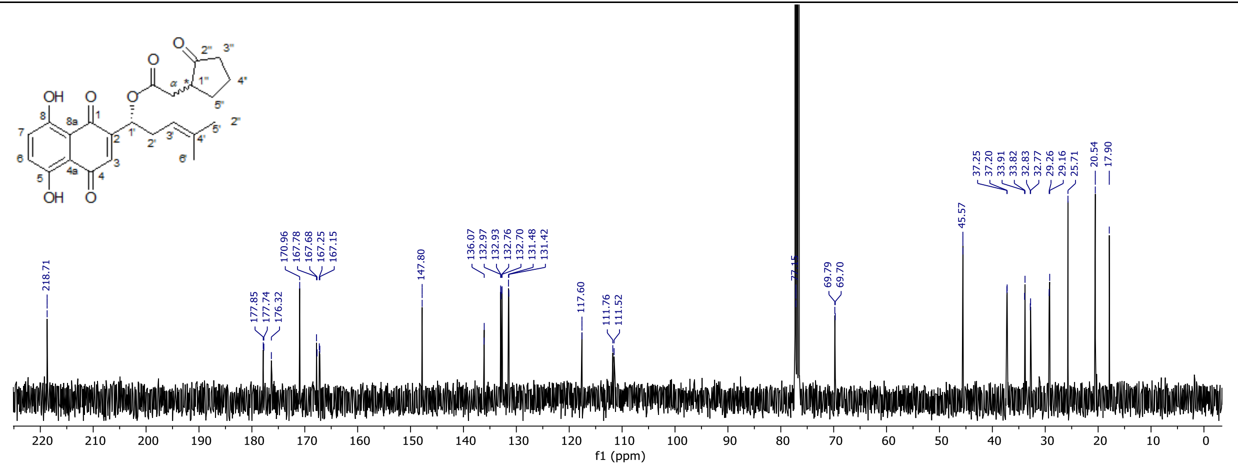

^{13}C-NMR spectrum ($CDCl_3$, 100 MHz) of **16**

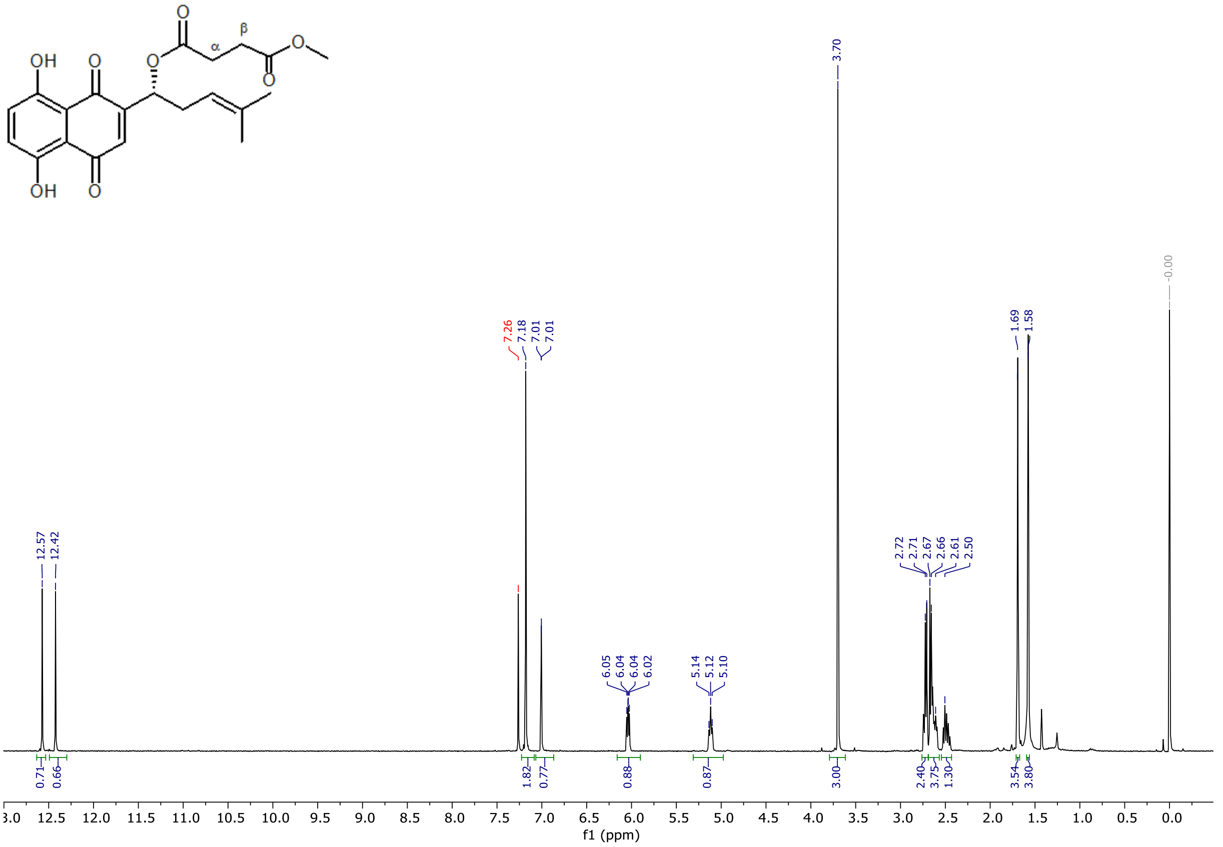

^{1}H-NMR spectrum (CDCl$_3$, 400 MHz) of **17**

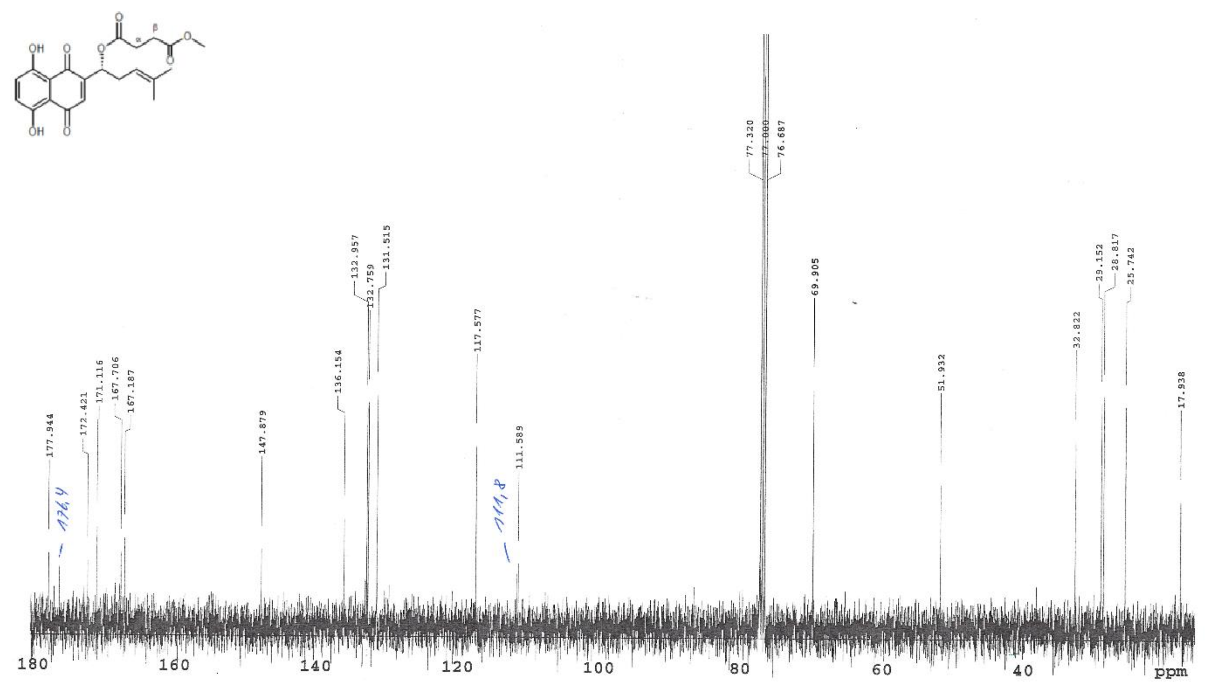

^{13}C-NMR spectrum (CDCl$_3$, 100 MHz) of **17**

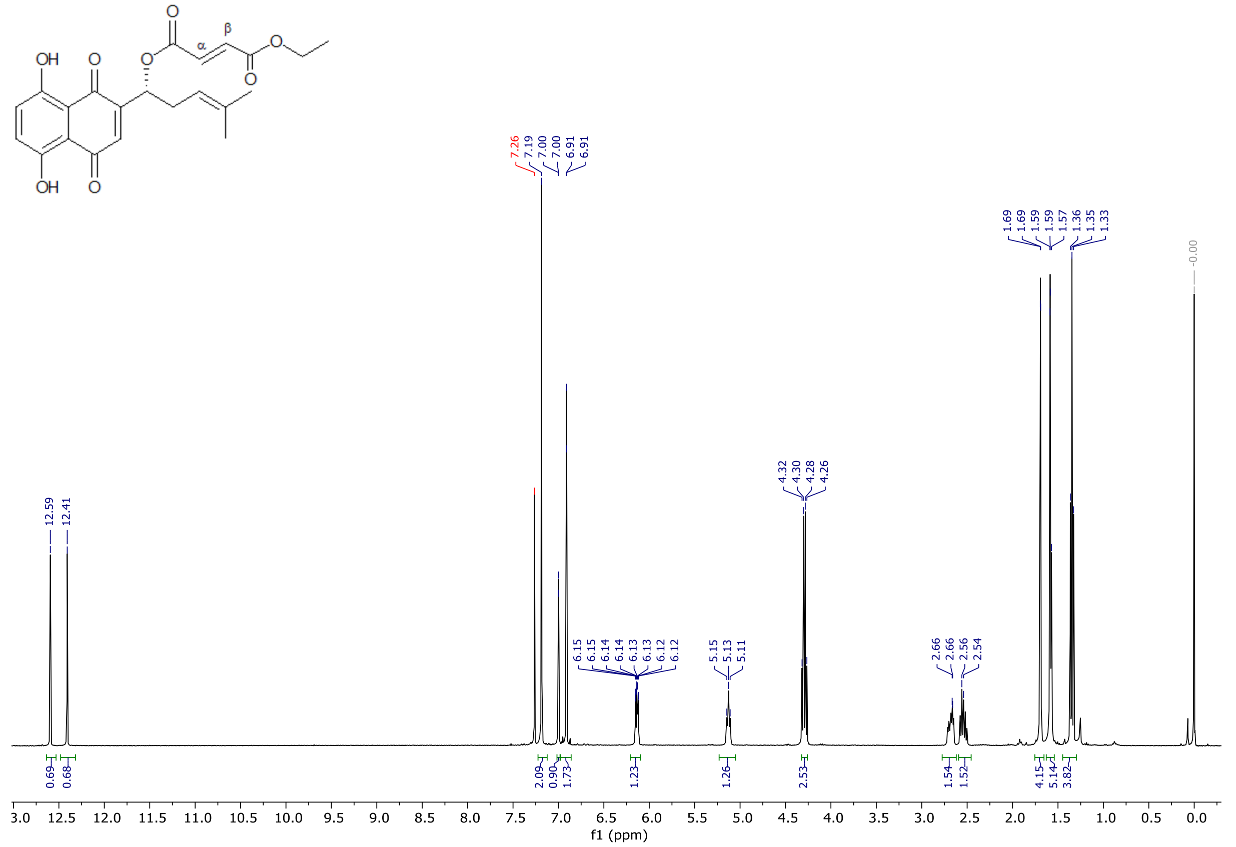

^{1}H-NMR spectrum ($CDCl_3$, 400 MHz) of **18**

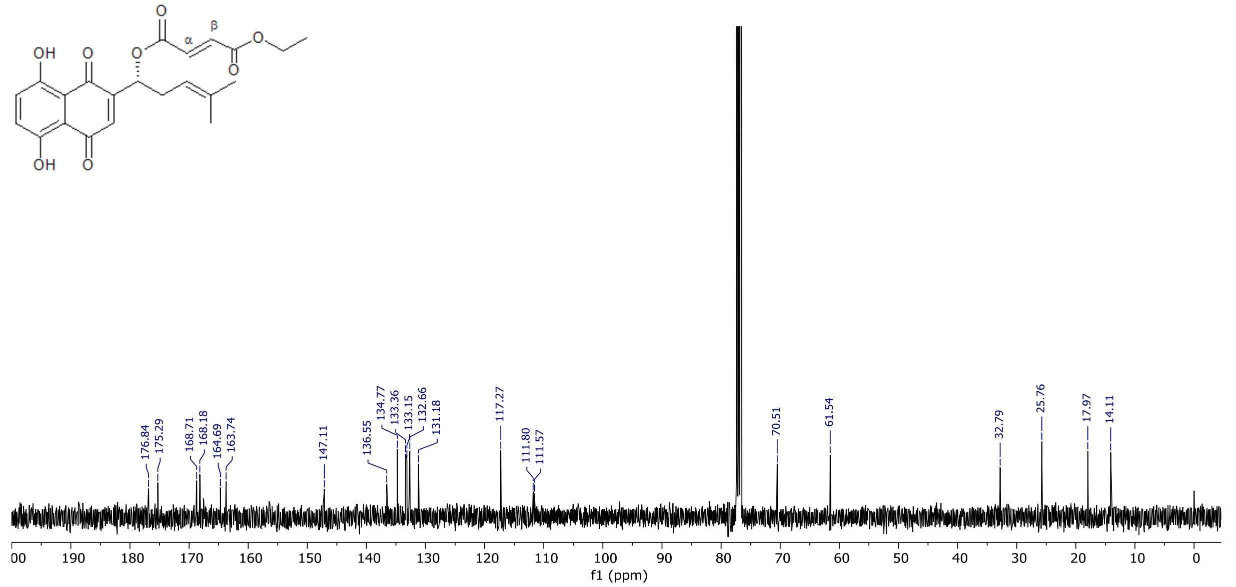

^{13}C-NMR spectrum ($CDCl_3$, 100 MHz) of **18**

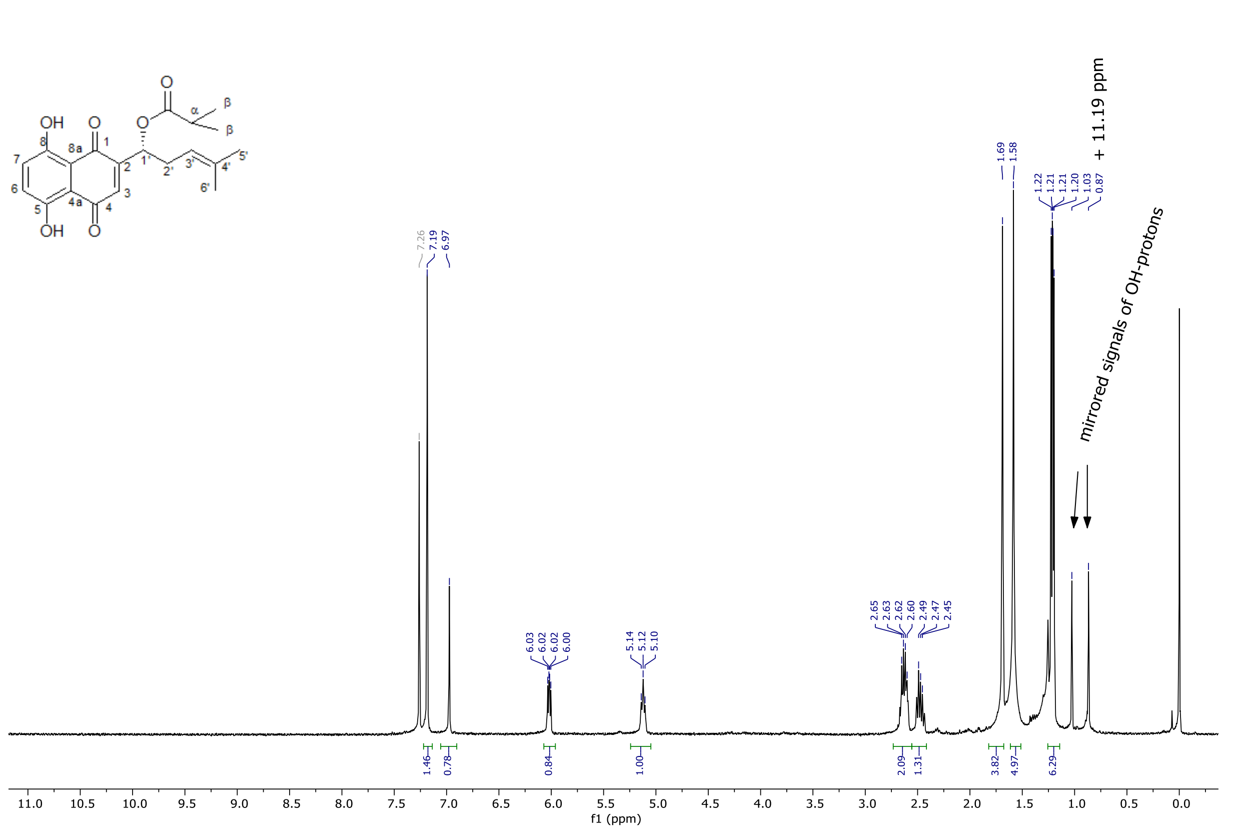

^{1}H-NMR spectrum ($CDCl_3$, 400 MHz) of **19**

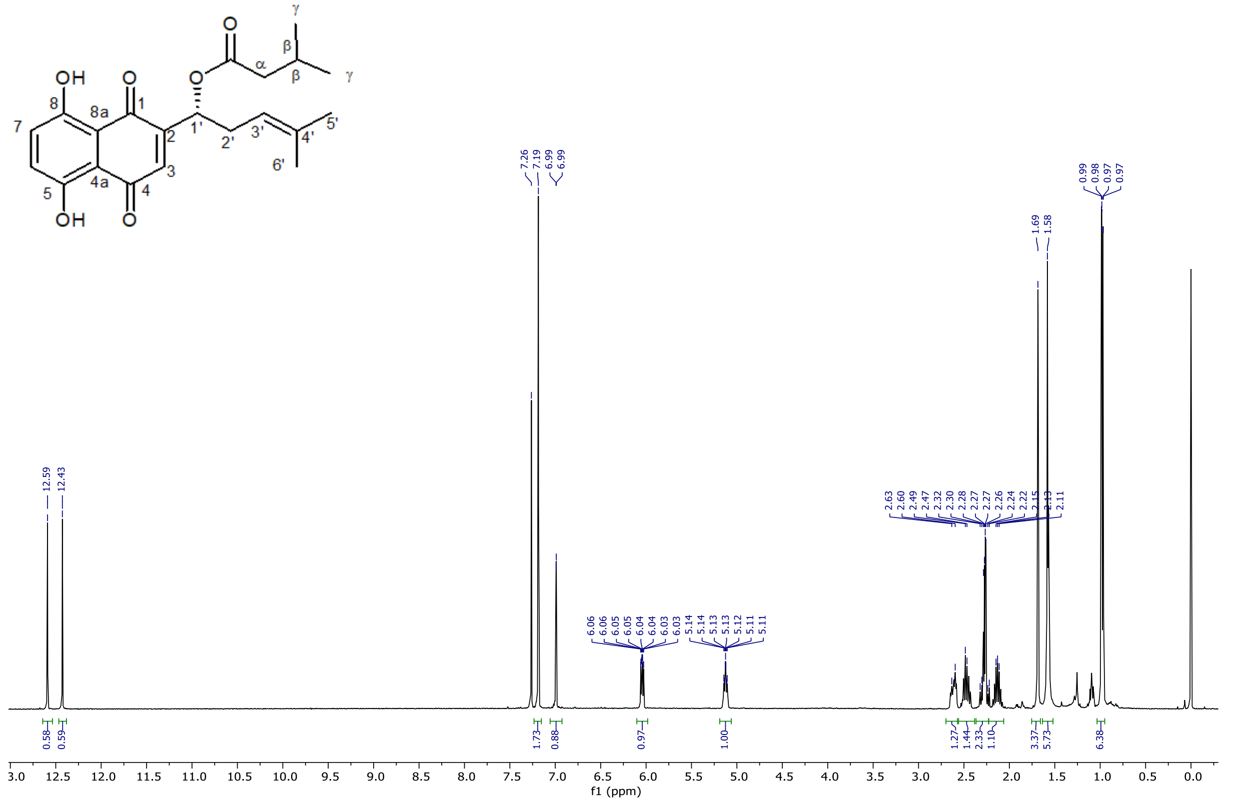

^{1}H-NMR spectrum ($CDCl_3$, 400 MHz) of **20**

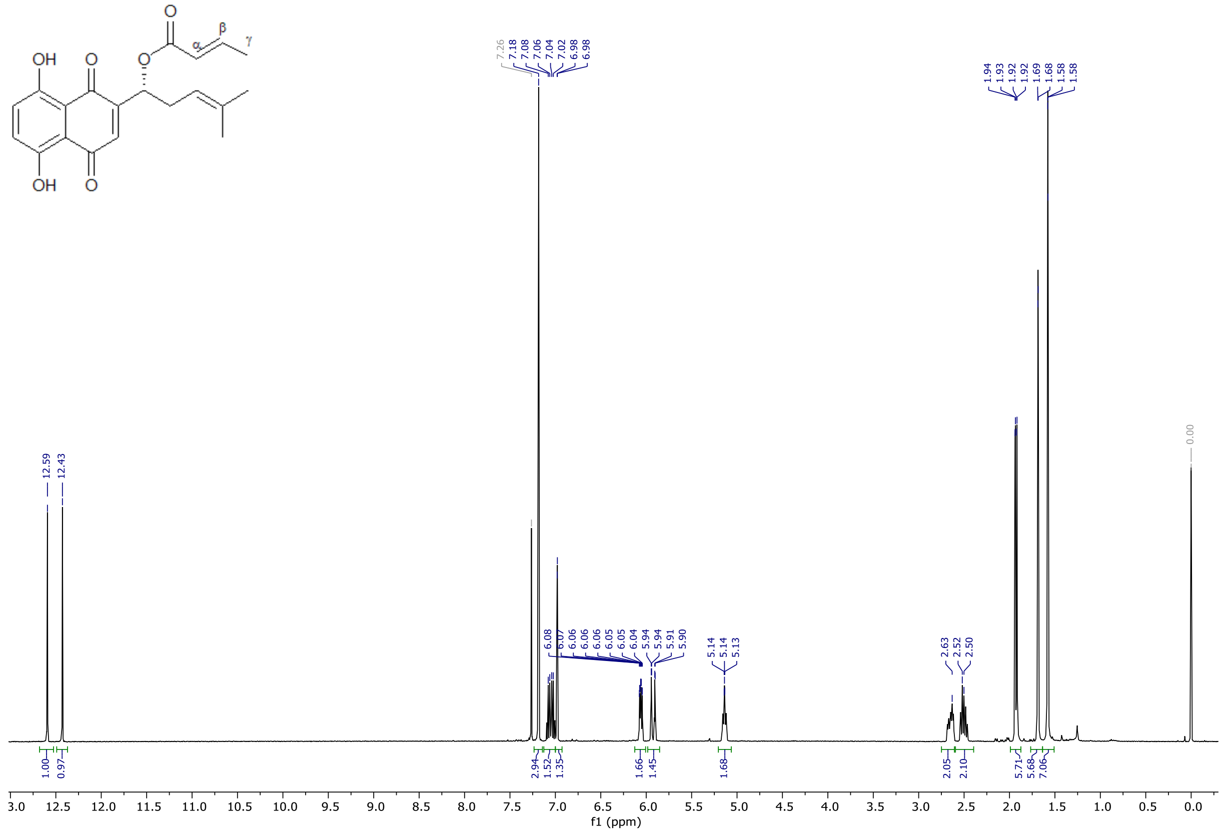

^{1}H-NMR spectrum (CDCl$_3$, 400 MHz) of **21**

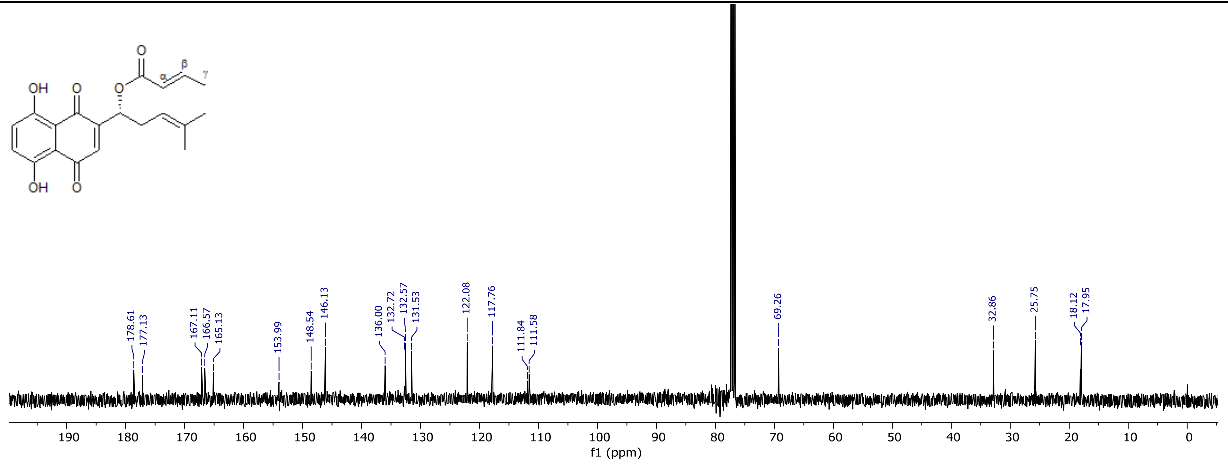

^{13}C-NMR spectrum (CDCl$_3$, 100 MHz) of **21**

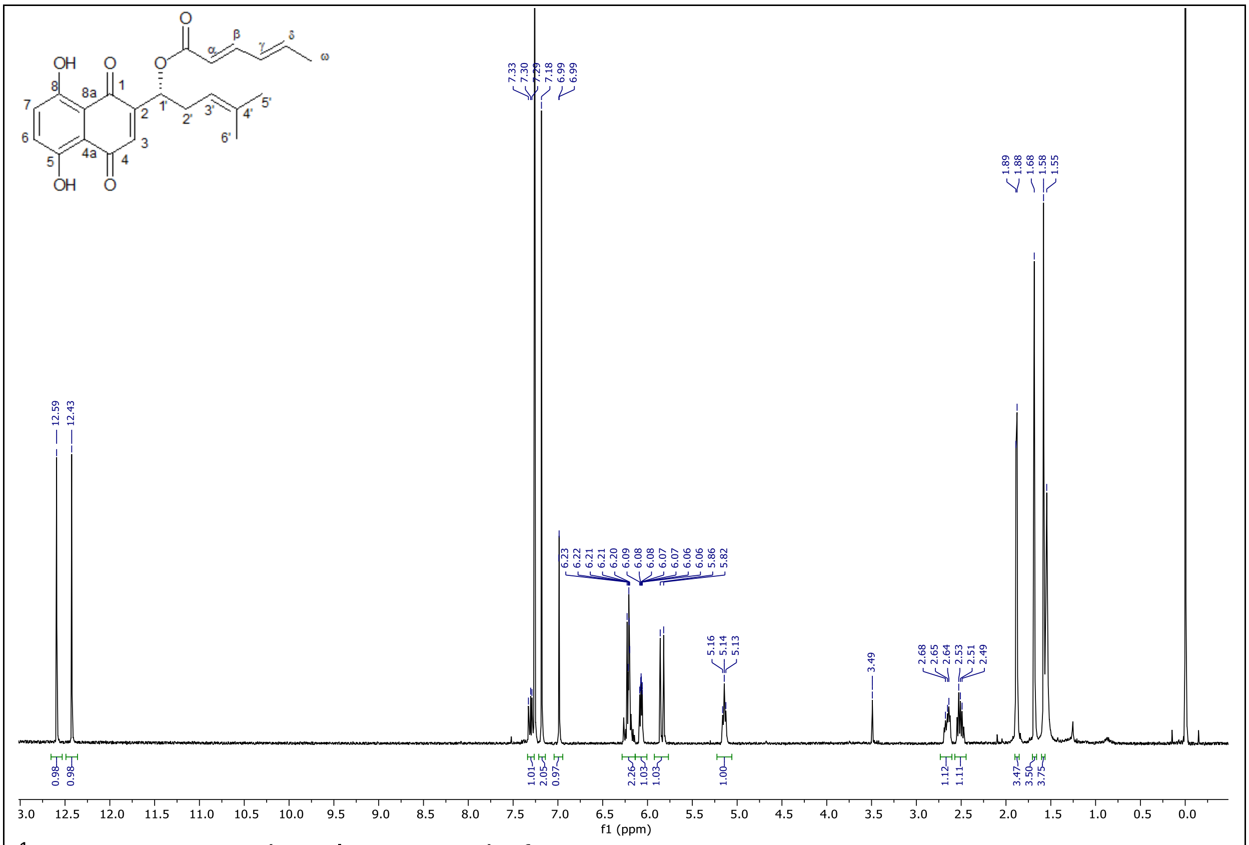

^{1}H-NMR spectrum (CDCl$_3$, 400 MHz) of **22**

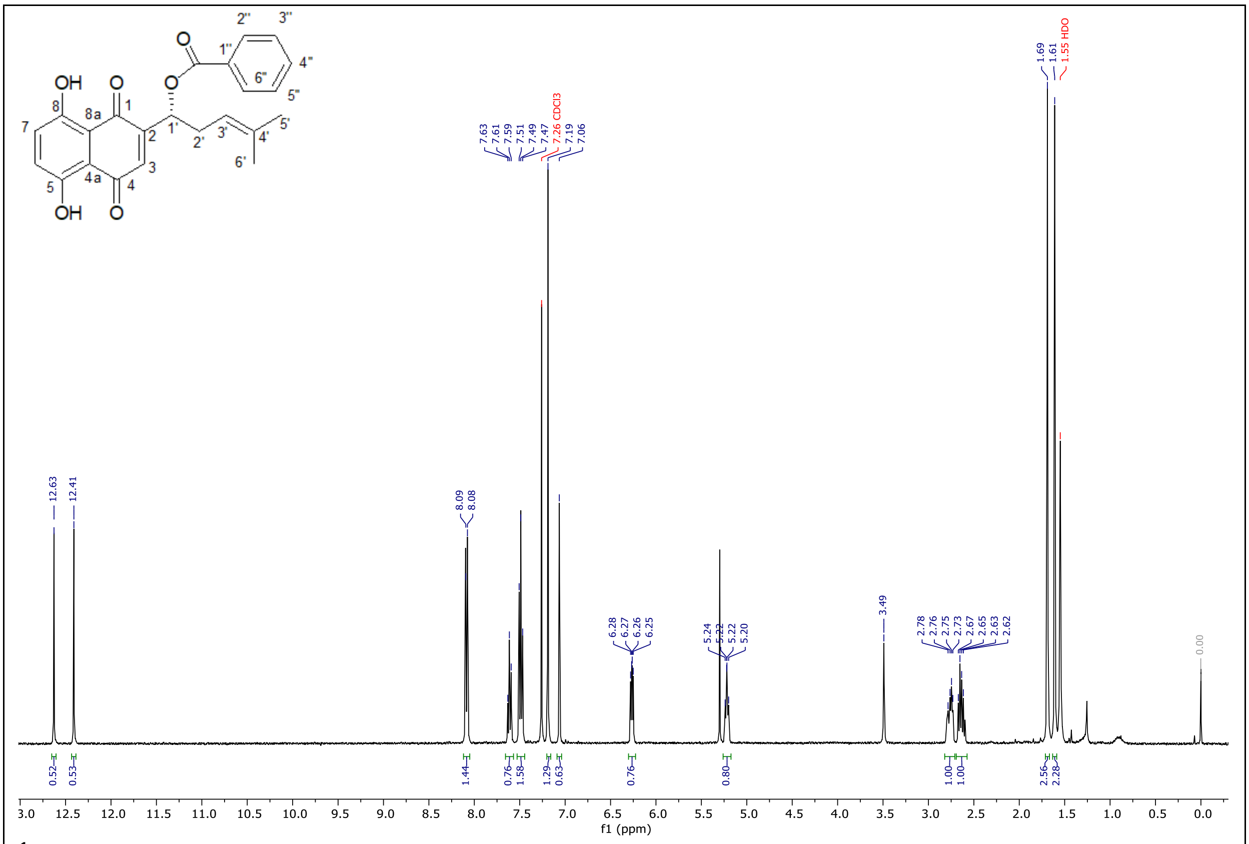

^{1}H-NMR spectrum (CDCl$_3$, 400 MHz) of **23**

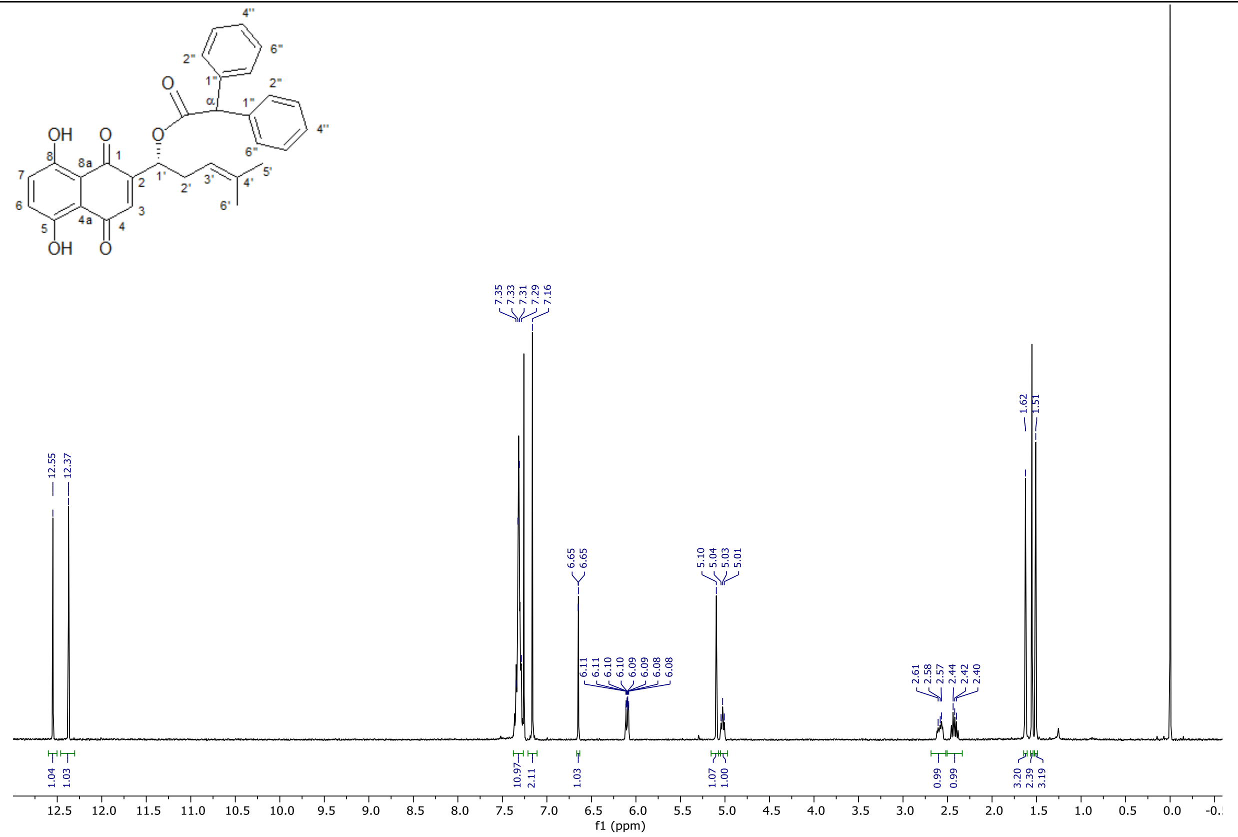

^{1}H-NMR spectrum ($CDCl_3$, 400 MHz) of **24**

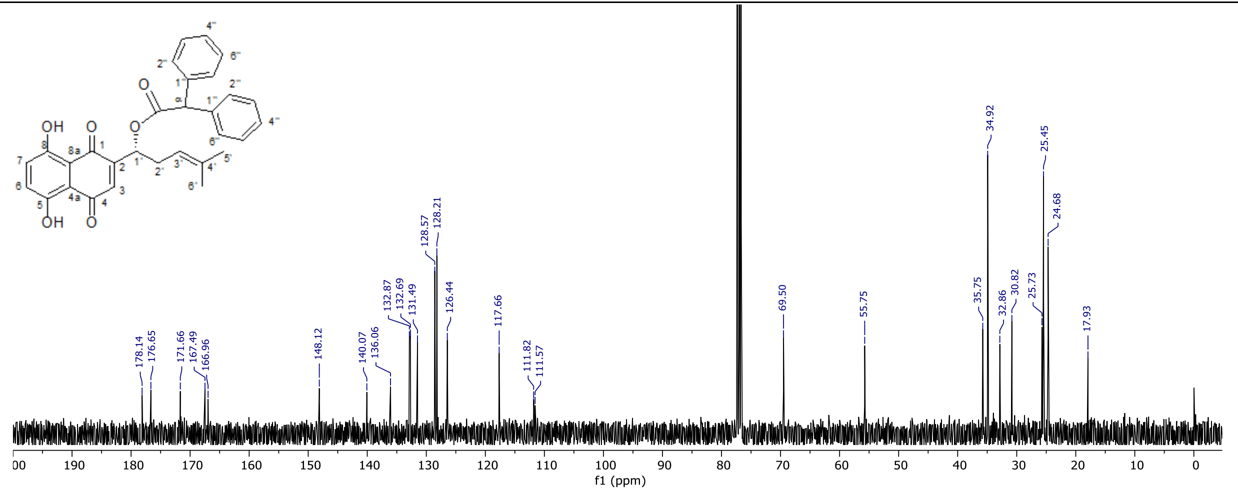

^{13}C-NMR spectrum ($CDCl_3$, 100 MHz) of **24**

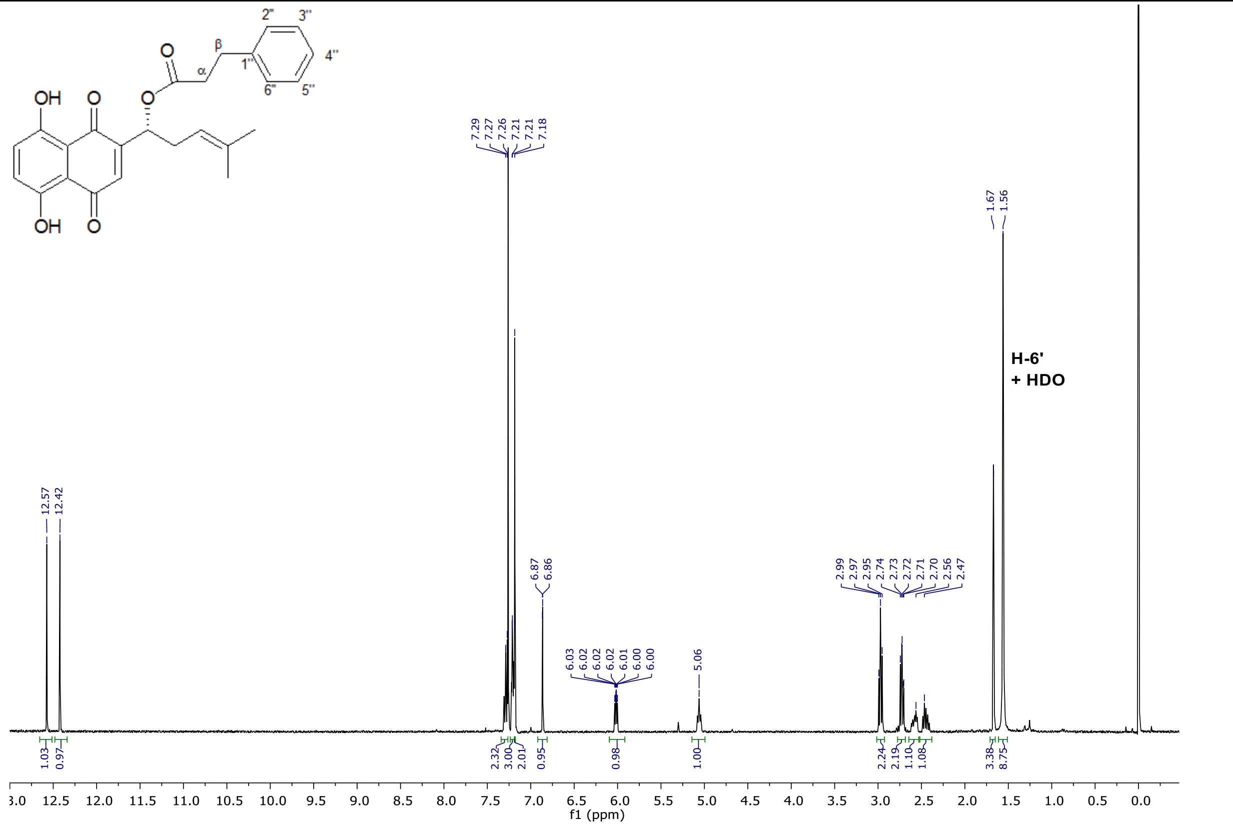

^{1}H-NMR spectrum ($CDCl_3$, 400 MHz) of **25**

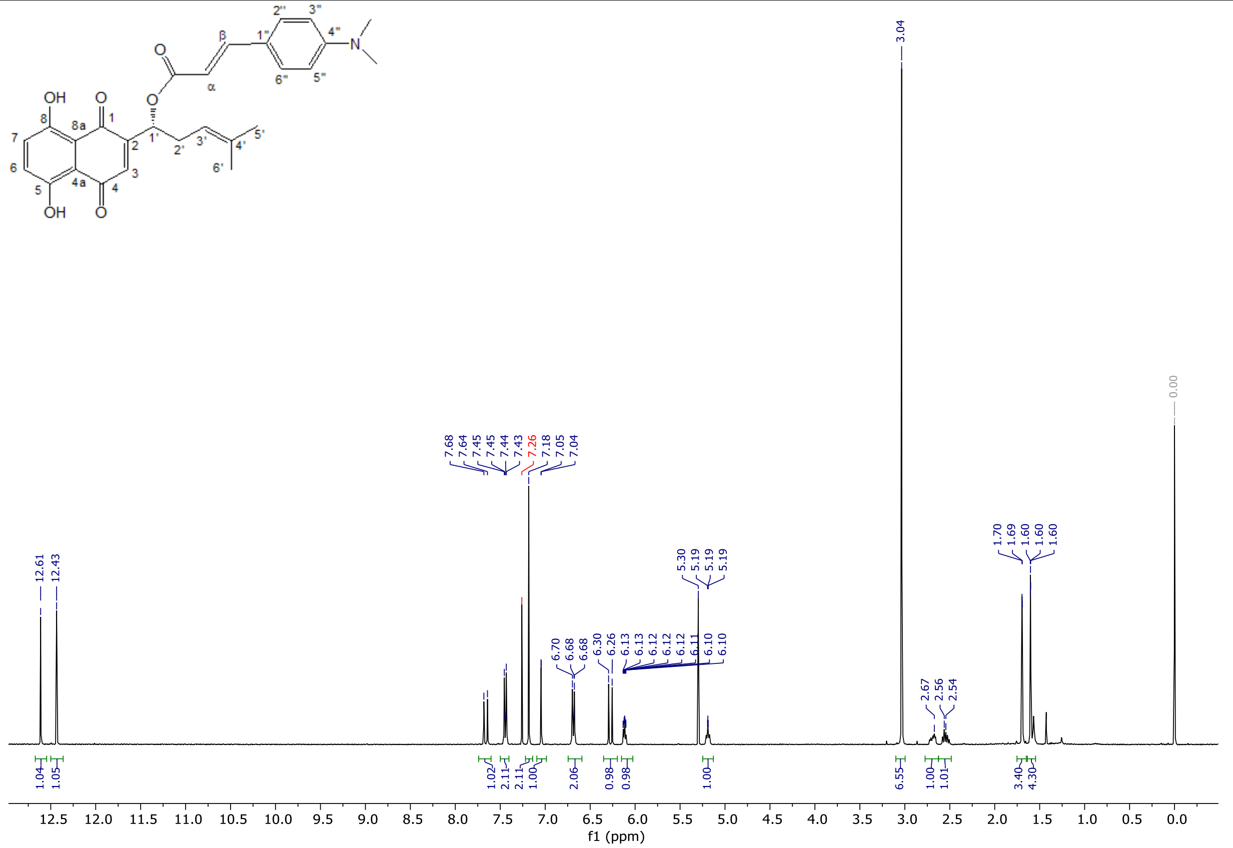

^{1}H-NMR spectrum ($CDCl_3$, 400 MHz) of **26**

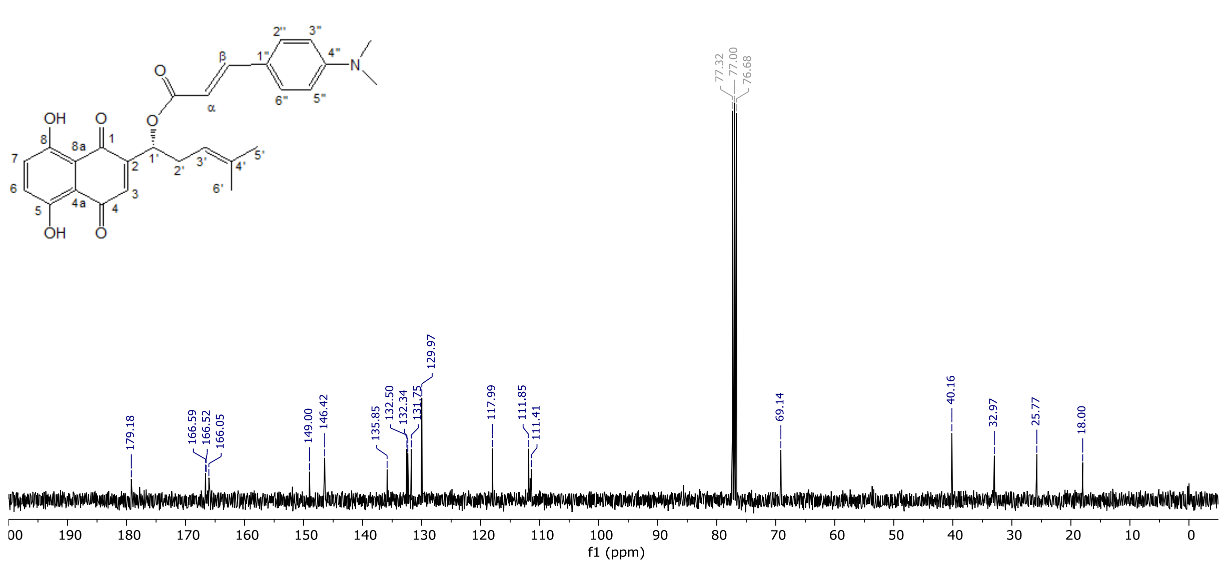

^{13}C-NMR spectrum ($CDCl_3$, 100 MHz) of **26**

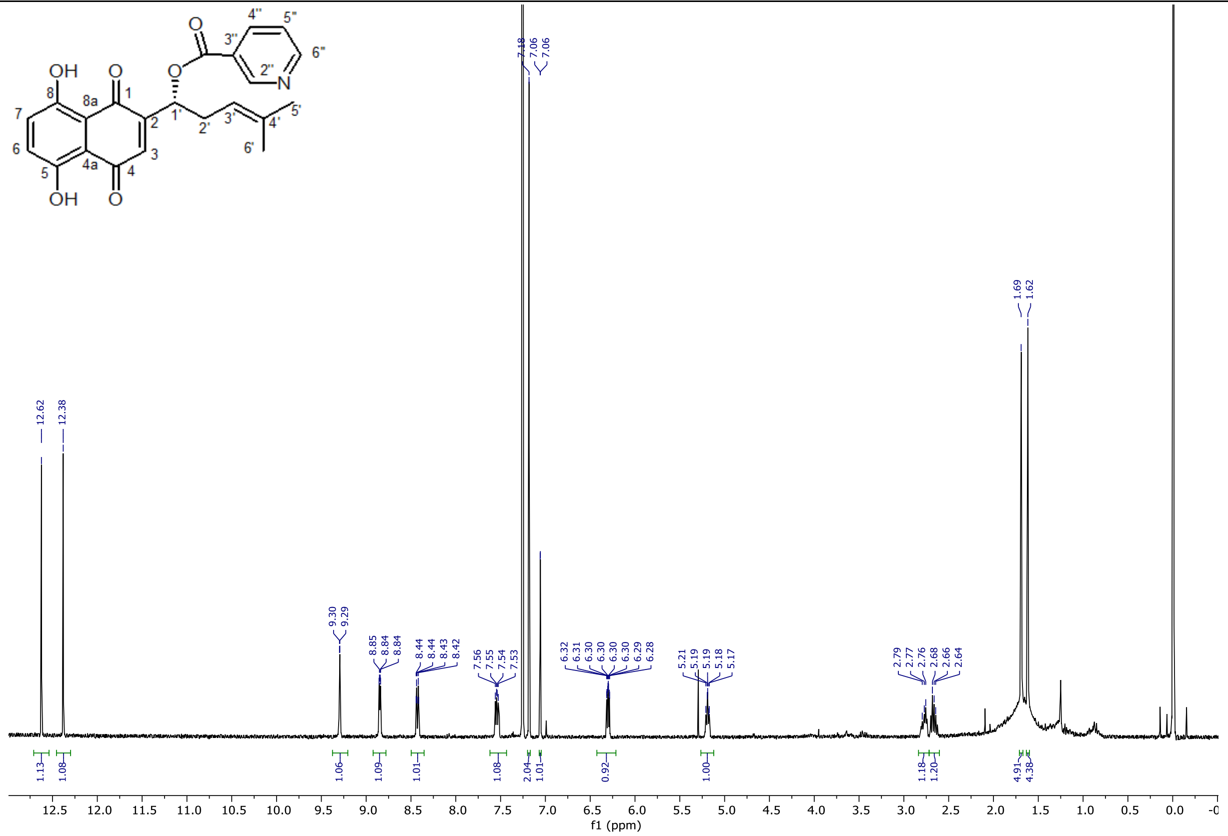

^{1}H-NMR spectrum ($CDCl_3$, 400 MHz) of **27**

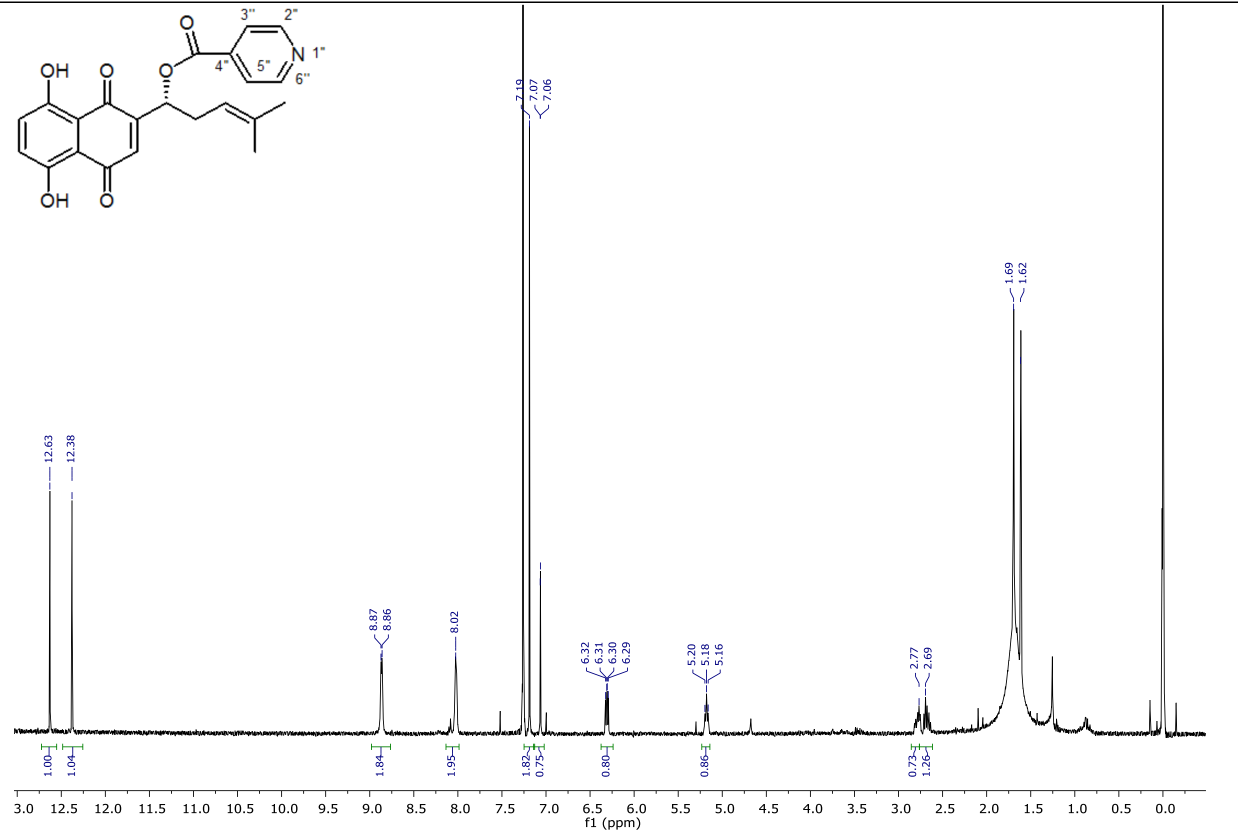

^{1}H-NMR spectrum ($CDCl_3$, 400 MHz) of **28**

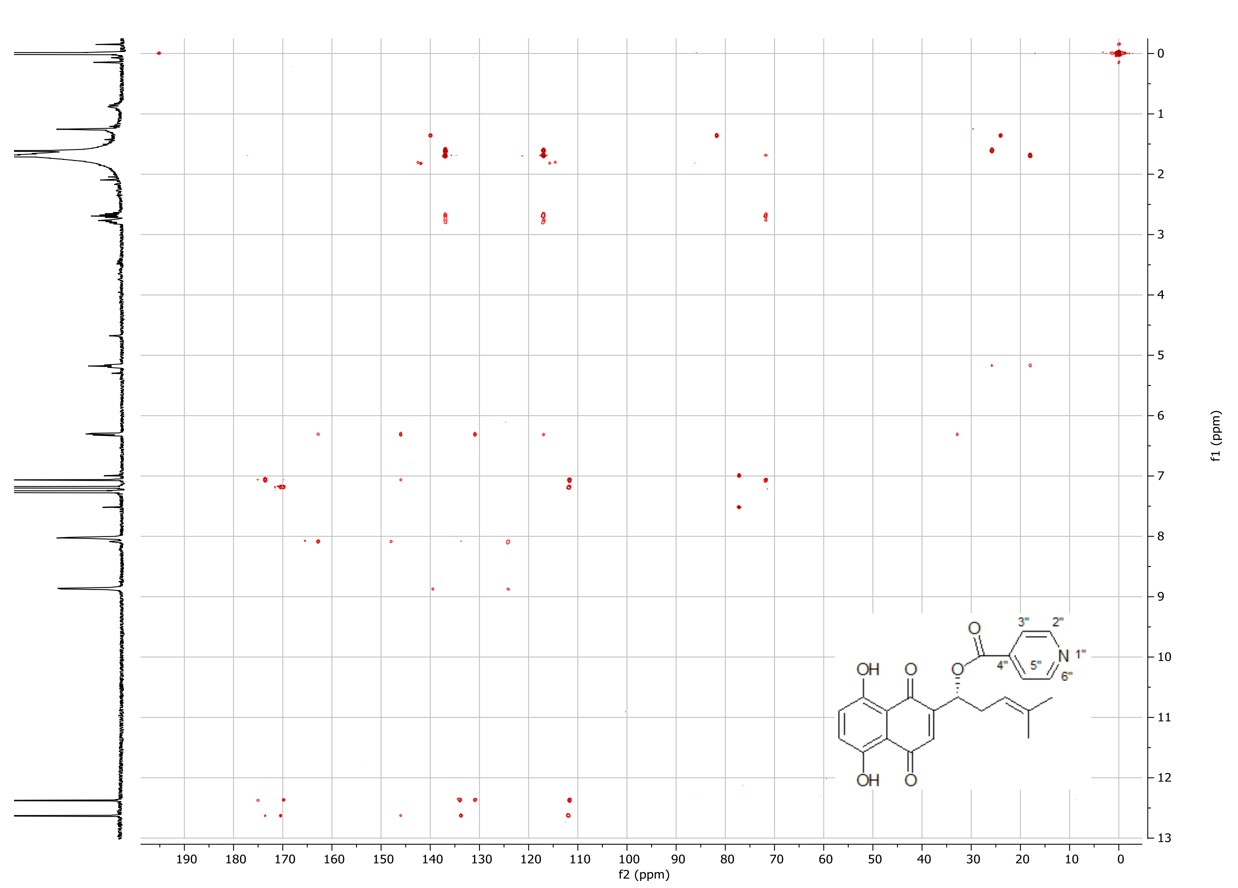

HMBC spectrum ($CDCl_3$, CD_3OD 100 MHz) of **28**

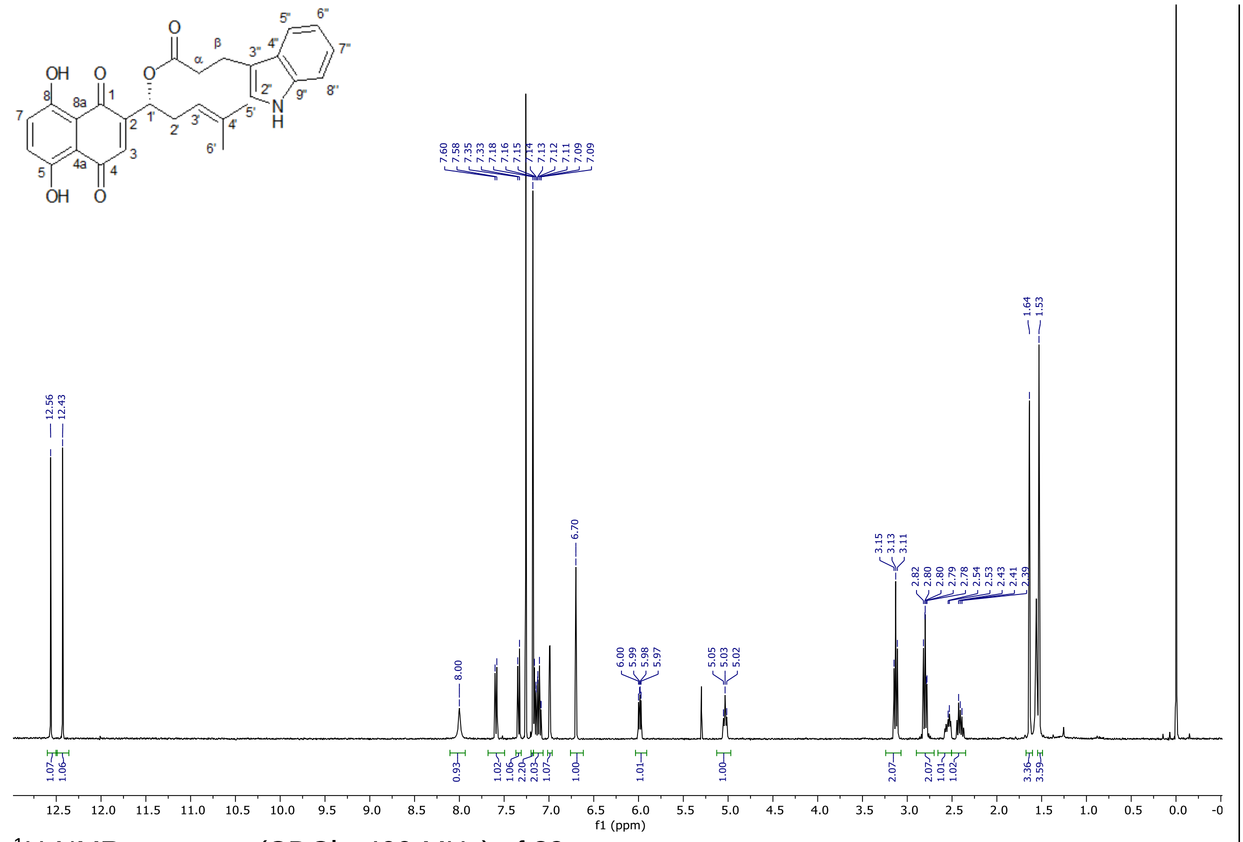

^{1}H-NMR spectrum ($CDCl_3$, 400 MHz) of **29**

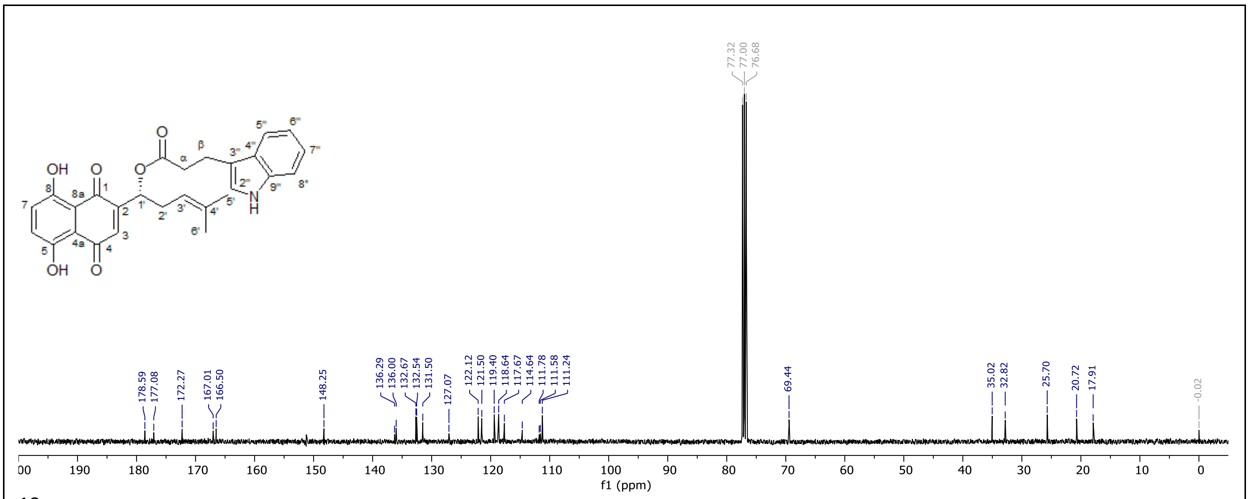

^{13}C-NMR spectrum ($CDCl_3$, 100 MHz) of **29**

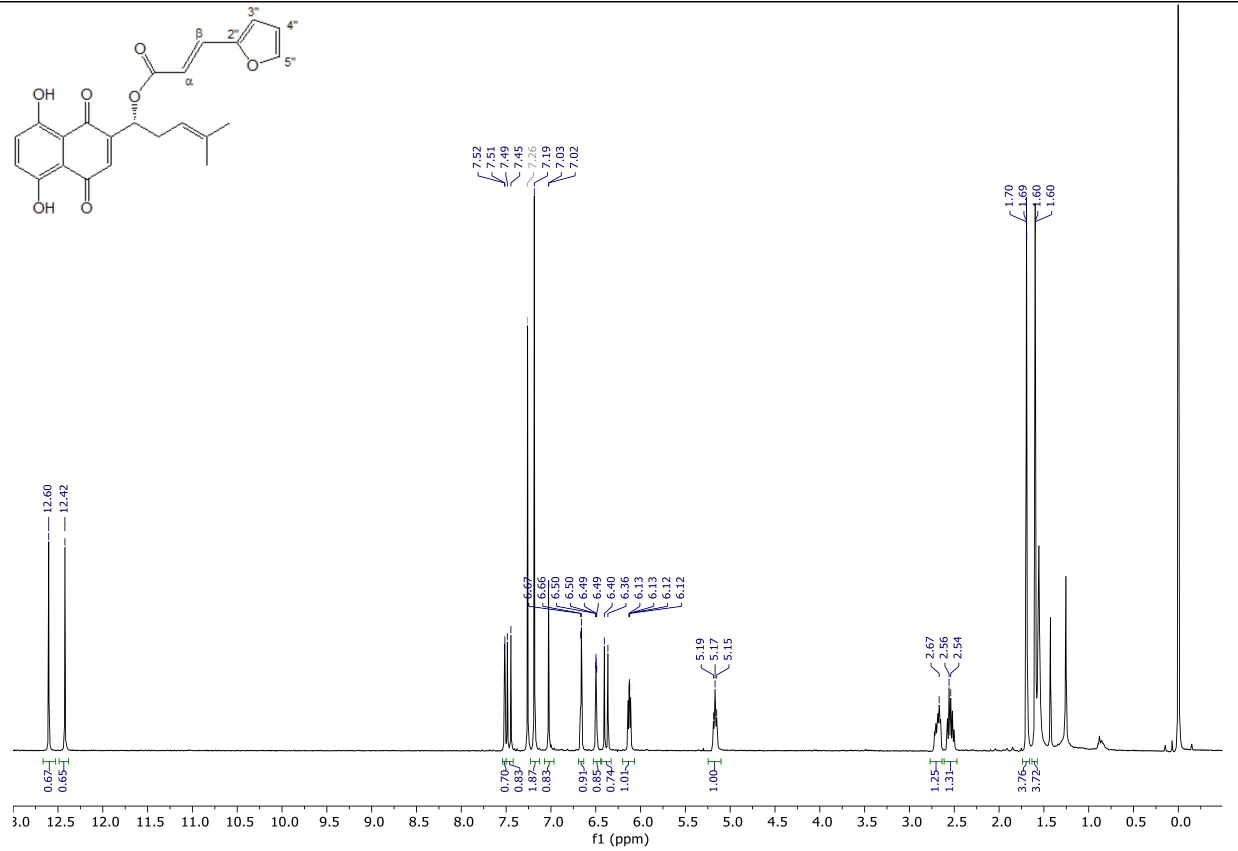

^{1}H-NMR spectrum ($CDCl_3$, 400 MHz) of **30**

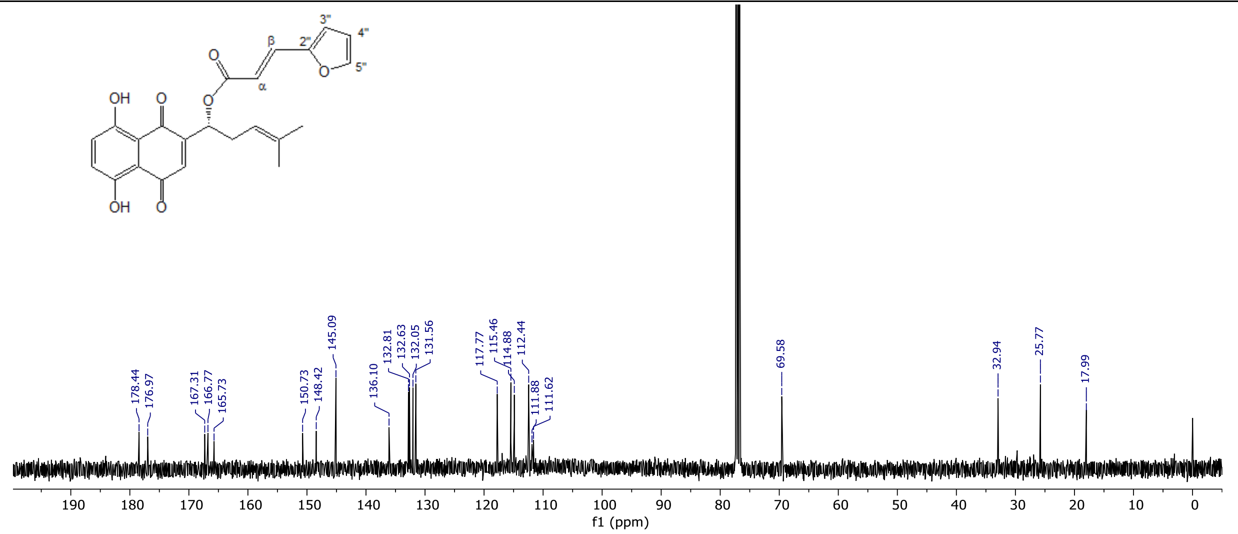

^{13}C-NMR spectrum ($CDCl_3$, 100 MHz) of **30**

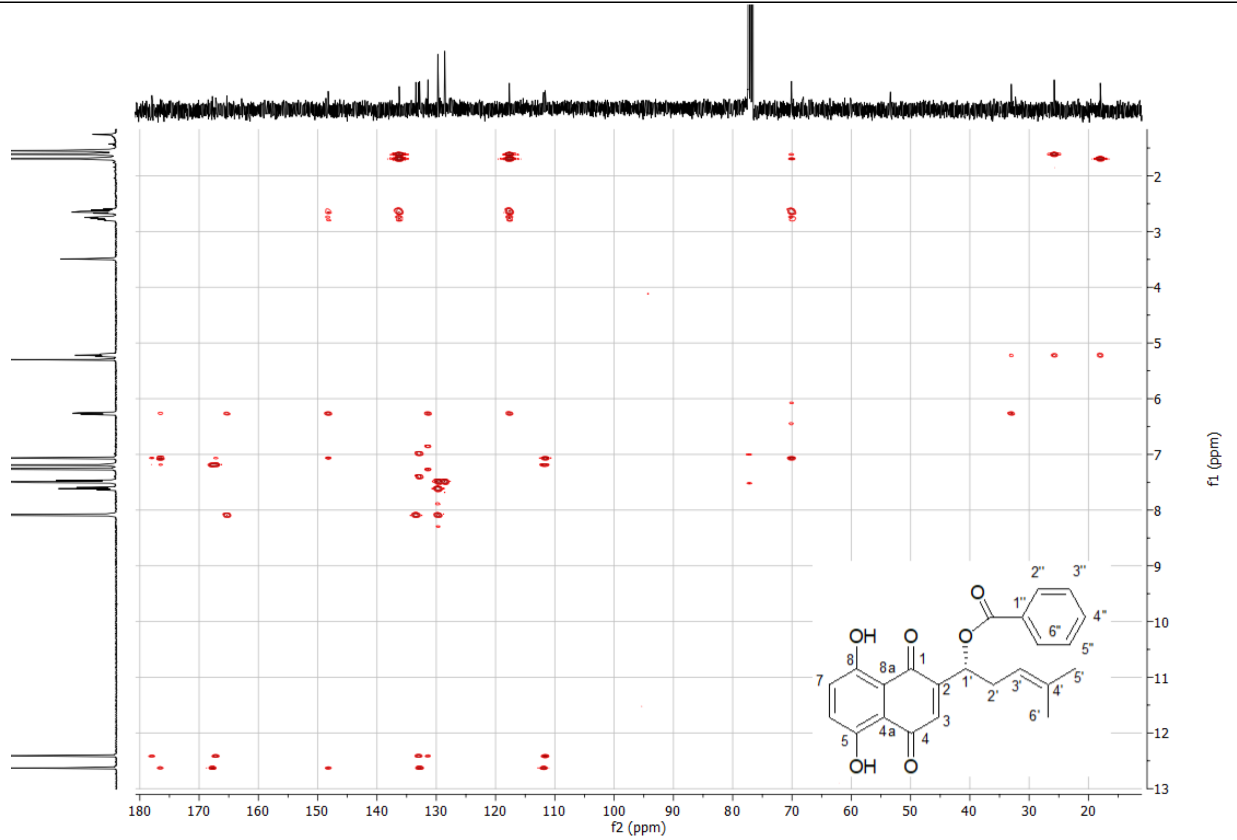

HMBC spectrum ($CDCl_3$, 100 MHz) of **31**

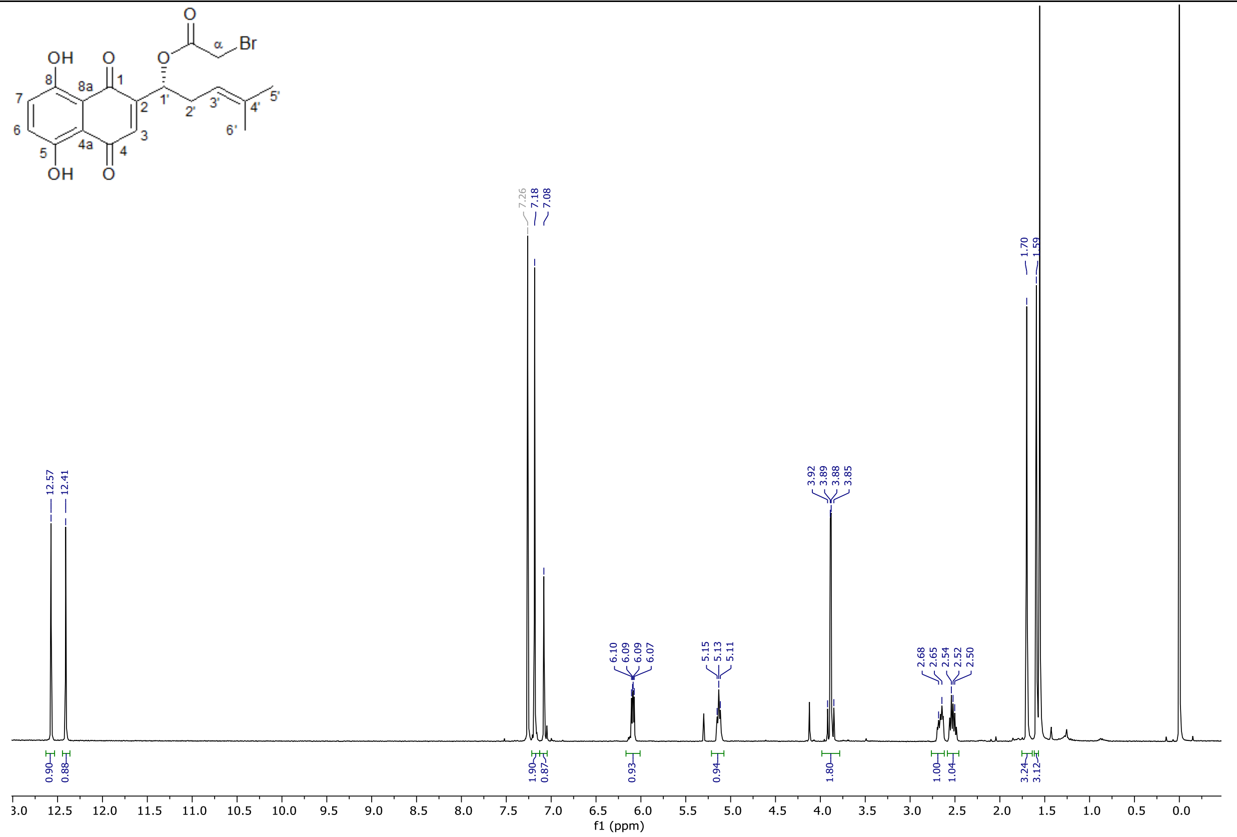

^{1}H-NMR spectrum ($CDCl_3$, 100 MHz) of **31**

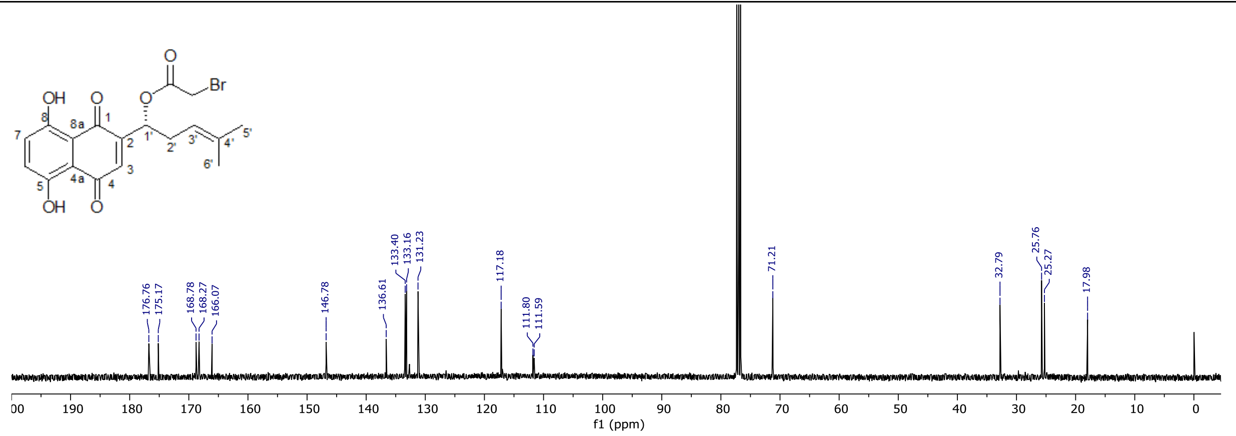

^{13}C-NMR spectrum ($CDCl_3$, 100 MHz) of **31**

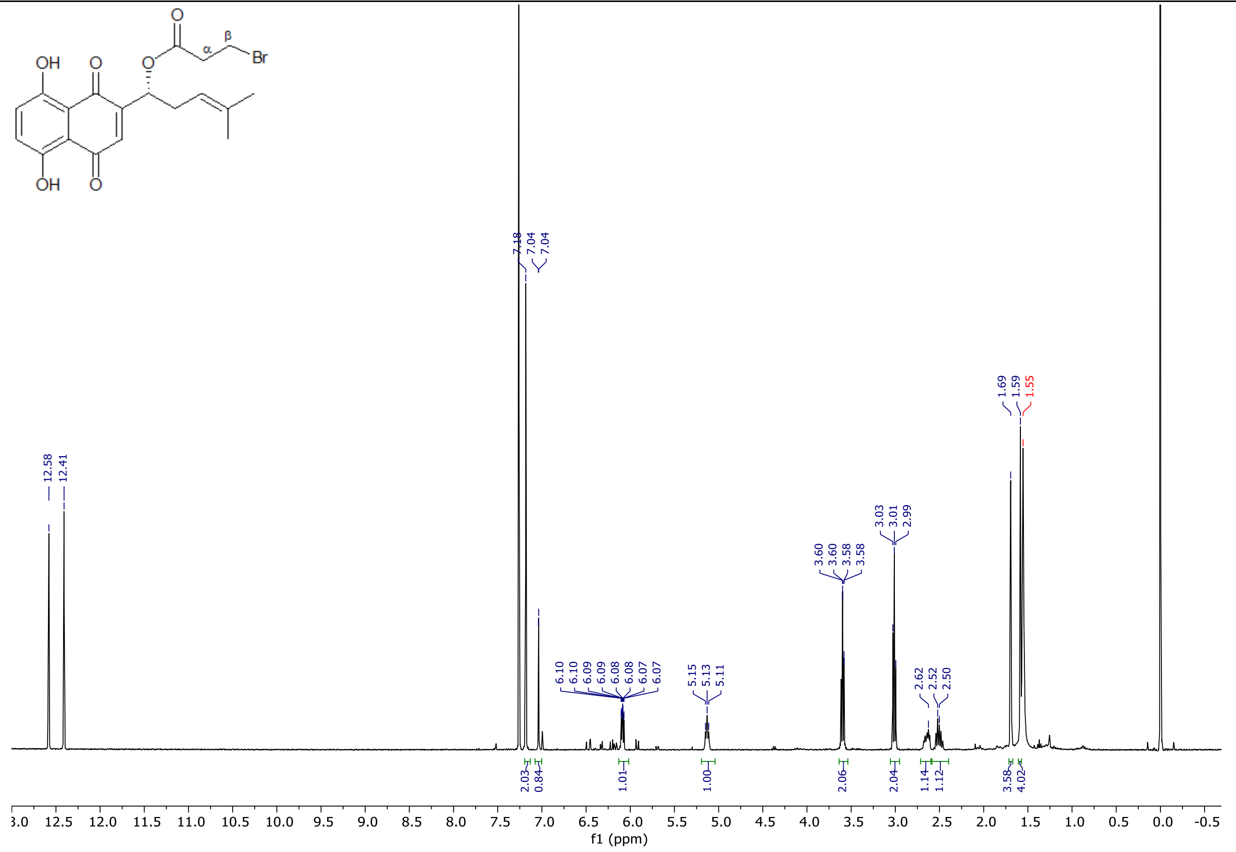

^{1}H-NMR spectrum ($CDCl_3$, 400 MHz) of **32**

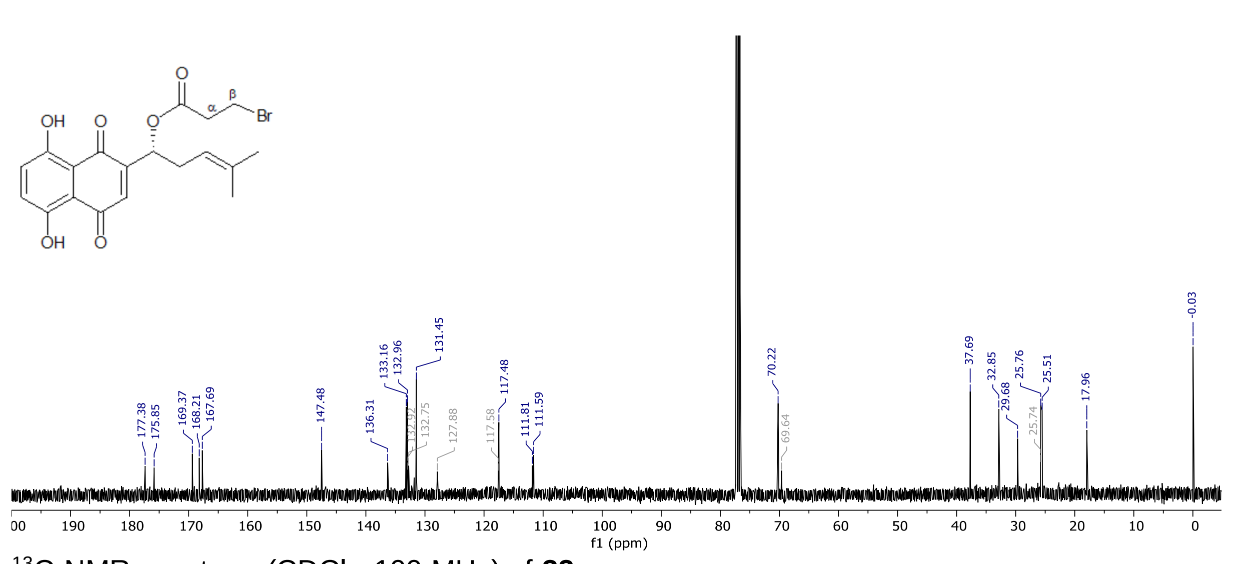

^{13}C-NMR spectrum ($CDCl_3$, 100 MHz) of **32**

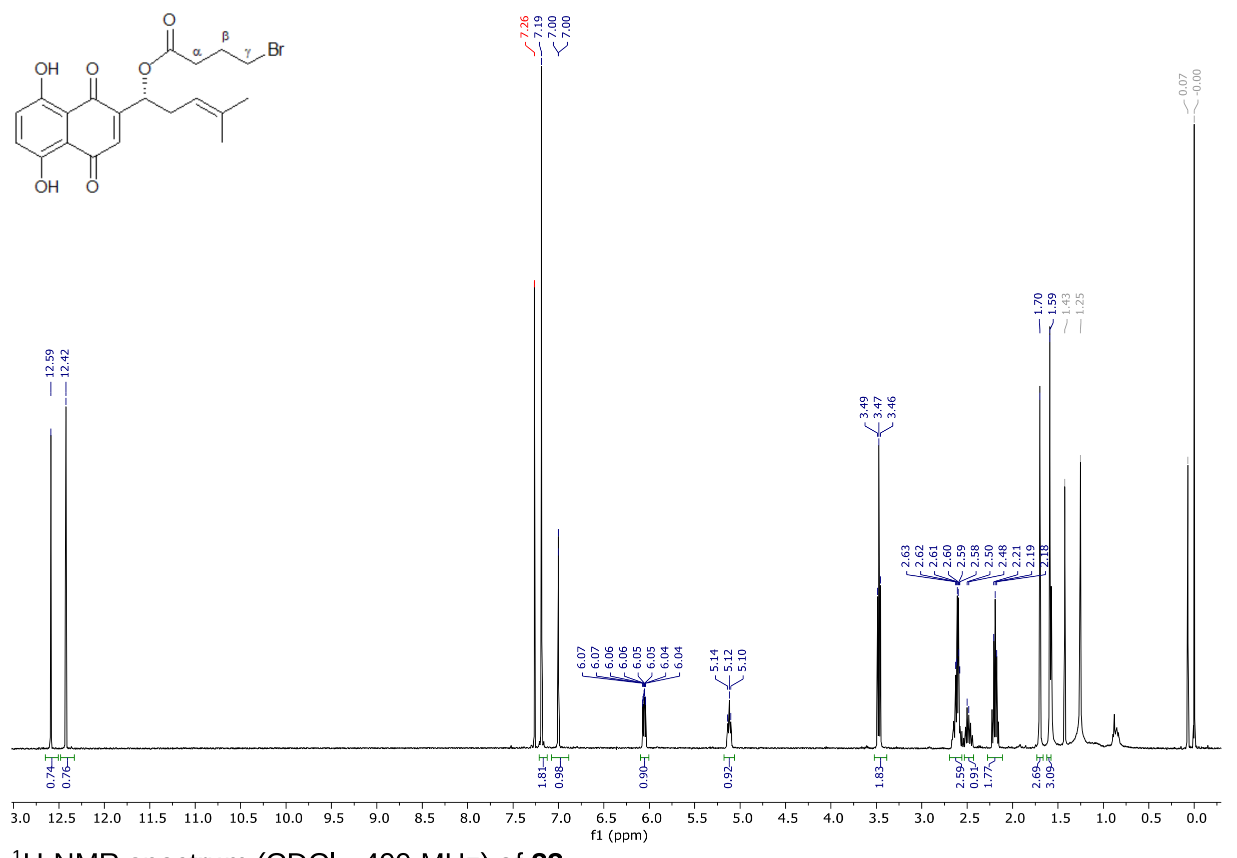

^{1}H-NMR spectrum ($CDCl_3$, 400 MHz) of **33**

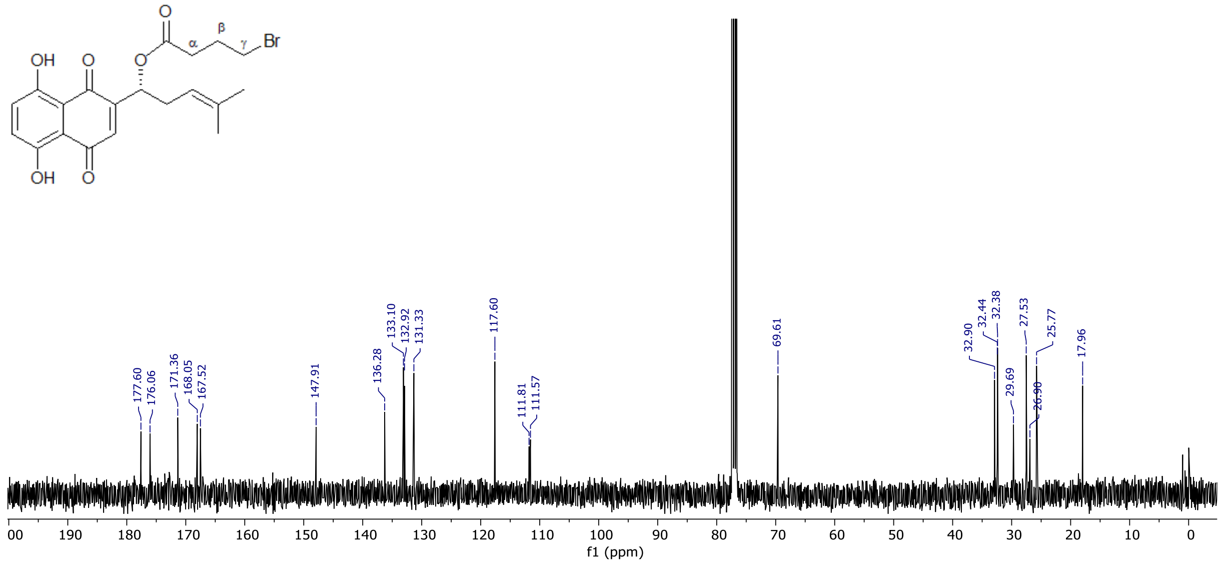

^{13}C-NMR spectrum ($CDCl_3$, 100 MHz) of **33**

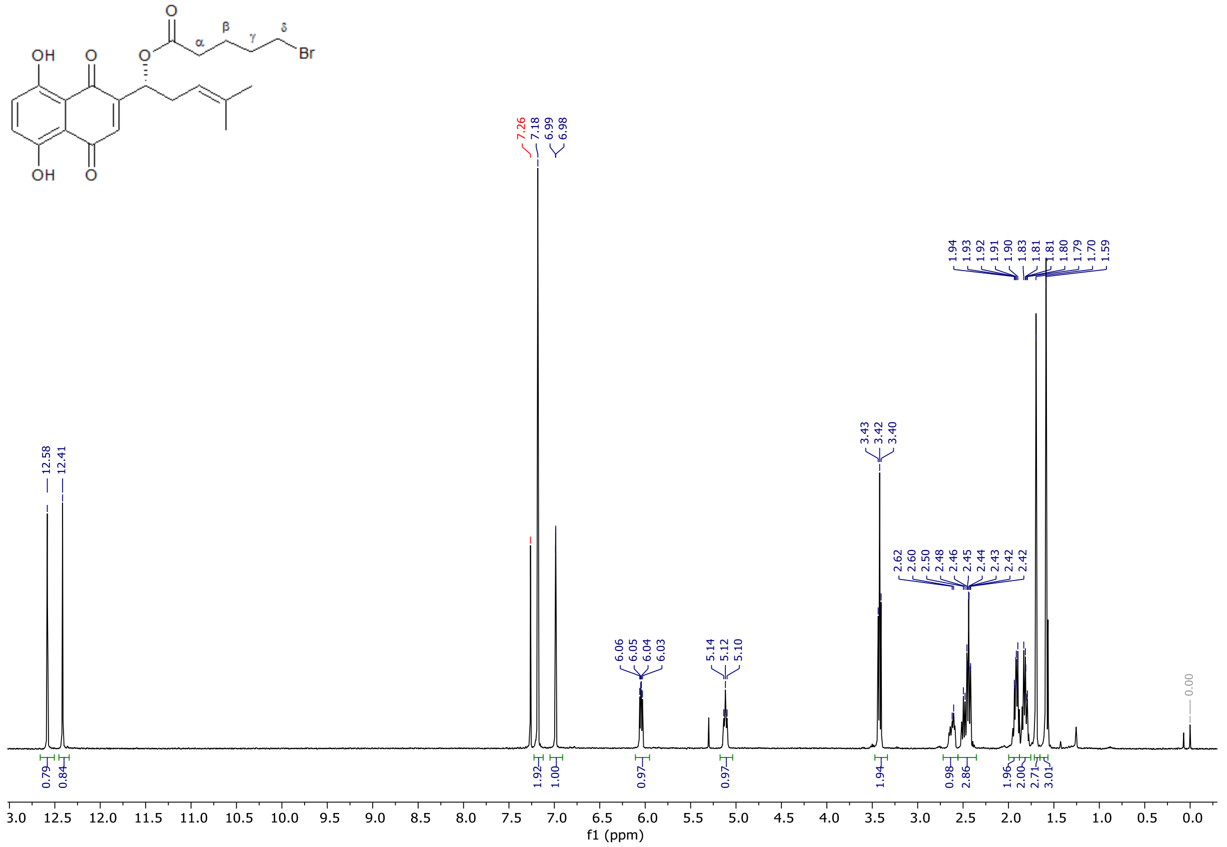

^{1}H-NMR spectrum ($CDCl_3$, 400 MHz) of **34**

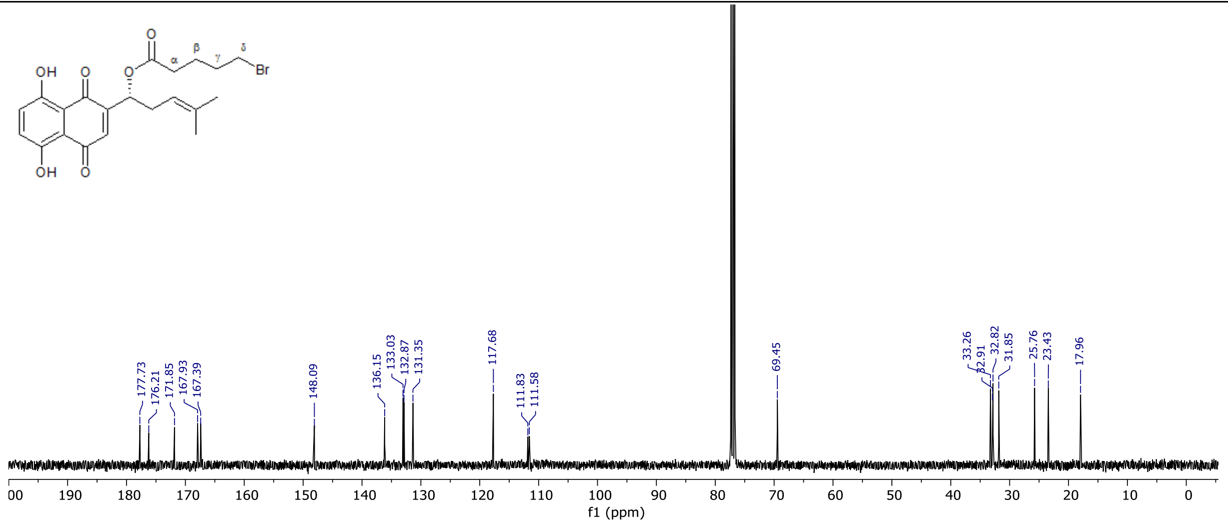

^{13}C-NMR spectrum ($CDCl_3$, 100 MHz) of **34**

3. ¹H NMR spectra of precursor acids

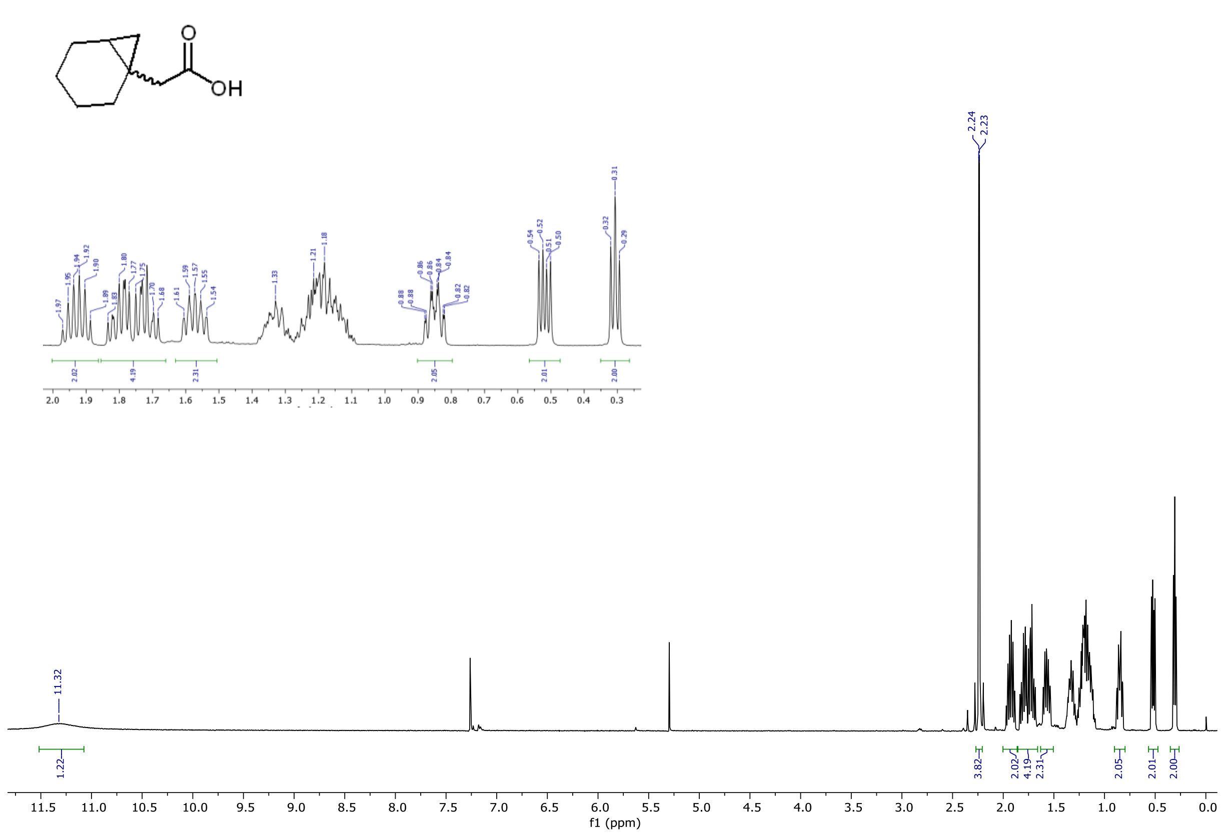

¹H-NMR spectrum ($CDCl_3$, 400 MHz) of **p1**

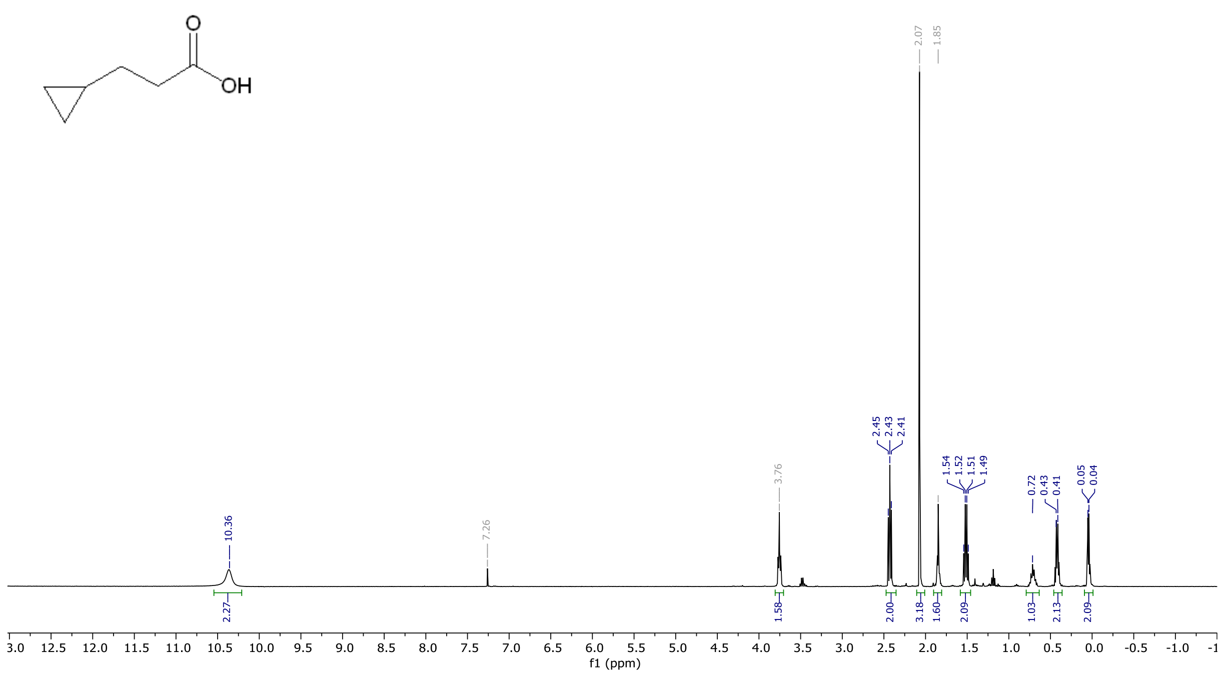

¹H-NMR spectrum ($CDCl_3$, 400 MHz) of **p2**

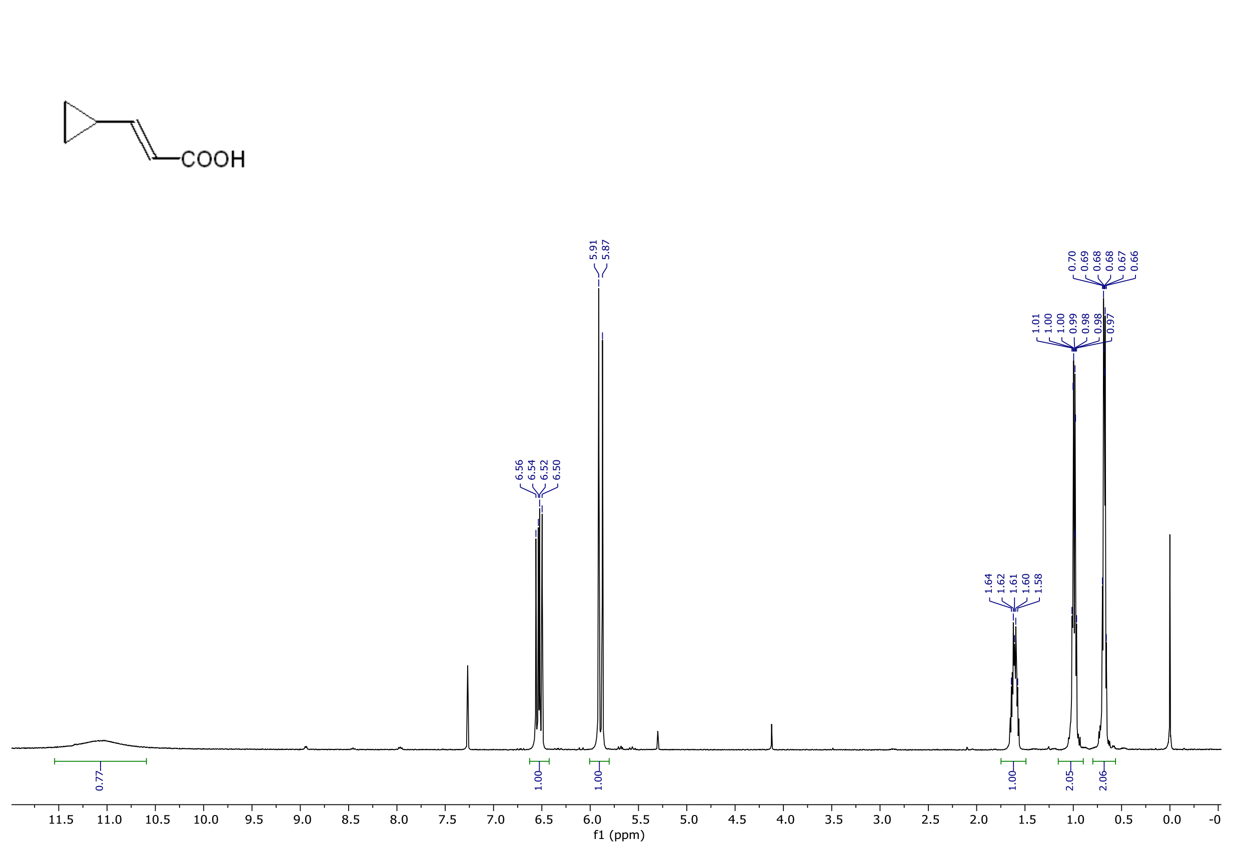

^{1}H-NMR spectrum ($CDCl_3$, 400 MHz) of **p3**

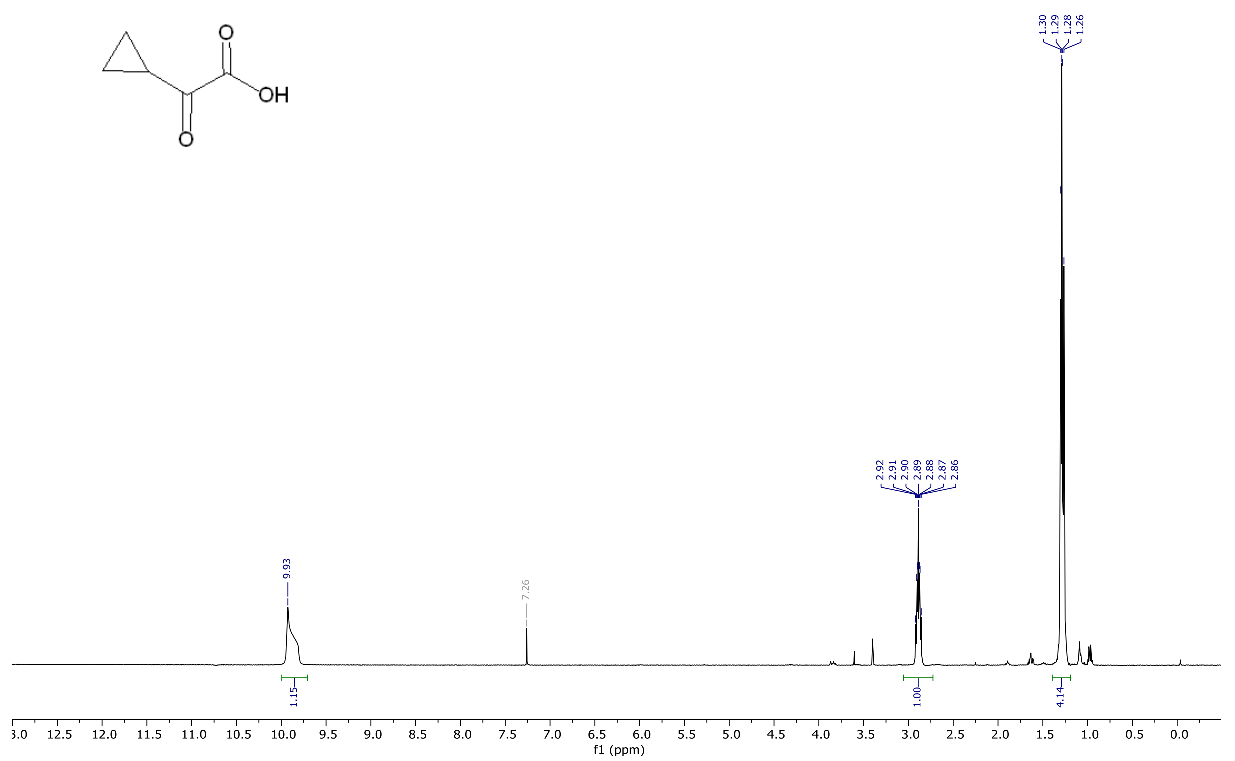

^{1}H-NMR spectrum ($CDCl_3$, 400 MHz) of **p4**
